# Genetics of coronary artery calcification among African Americans, a meta-analysis

**DOI:** 10.1186/1471-2350-14-75

**Published:** 2013-07-19

**Authors:** Mary K Wojczynski, Mingyao Li, Lawrence F Bielak, Kathleen F Kerr, Alex P Reiner, Nathan D Wong, Lisa R Yanek, Liming Qu, Charles C White, Leslie A Lange, Jane F Ferguson, Jing He, Taylor Young, Thomas H Mosley, Jennifer A Smith, Brian G Kral, Xiuqing Guo, Quenna Wong, Santhi K Ganesh, Susan R Heckbert, Michael E Griswold, Daniel H O’Leary, Matthew Budoff, J Jeffrey Carr, Herman A Taylor,, David A Bluemke, Serkalem Demissie, Shih-Jen Hwang, Dina N Paltoo, Joseph F Polak, Bruce M Psaty, Diane M Becker, Michael A Province, Wendy S Post, Christopher J O’Donnell, James G Wilson, Tamara B Harris, Maryam Kavousi, L Adrienne Cupples, Jerome I Rotter, Myriam Fornage, Lewis C Becker, Patricia A Peyser, Ingrid B Borecki, Muredach P Reilly

**Affiliations:** 1 Department of Genetics, Division of Statistical Genomics, Washington University School of Medicine, St. Louis, MO, USA; 2 Biostatistics and Epidemiology, University of Pennsylvania, Philadelphia, PA, USA; 3 Department of Epidemiology, University of Michigan School of Public Health, Ann Arbor, MI, USA; 4 Department of Biostatistics, University of Washington, Seattle, WA, USA; 5 Department of Epidemiology, University of Washington, Seattle, WA, USA; 6 Heart Disease Prevention Program, University of California, Irvine, CA, USA; 7 Department of Medicine, The Johns Hopkins University School of Medicine, Baltimore, MD, USA; 8 Department of Biostatistics, Boston University School of Public Health, Boston, MA, USA; 9 Department of Genetics, University of North Carolina, Chapel Hill, NC, USA; 10 The Cardiovascular Institute and Department of Medicine, Perelman School of Medicine, University of Pennsylvania, Philadelphia, PA, USA; 11 Program in Medical and Population Genetics, Broad Institute, Cambridge, MA, USA; 12 Department of Medicine, University of Mississippi Medical Center, Jackson, MS, USA; 13 Institute for Translational Genomics and Population Sciences, Los Angeles Biomedical Research Institute at Harbor-UCLA Medical Center, Torrance, CA, USA; 14 Division of Cardiovascular Medicine, Department of Internal Medicine, University of Michigan, Ann Arbor, MI, USA; 15 Center of Biostatistics and Bioinformatics, University of Mississippi Medical Center, Jackson, MS, USA; 16 Department of Radiology, Tufts University School of Medicine, Boston, MA, USA; 17 University of California Los Angeles School of Medicine, Los Angeles, CA, USA; 18 Department of Radiology, Wake Forest University School of Medicine, Winston-Salem, NC, USA; 19 Jackson State University, Tougaloo College, Jackson, MS, USA; 20 The University of Mississippi Medical Center, Jackson, MS, USA; 21 Radiology and Imaging Sciences, National Institutes of Health Clinical Center, Bethesda, MD, USA; 22 National Heart, Lung, and Blood Institute’s Framingham Heart Study and the Center for Population Studies, Framingham, MA, USA; 23 Advanced Technologies and Surgery Branch, Division of Cardiovascular Sciences, National Heart, Lung, and Blood Institute, National Institutes of Health, Bethesda, MD, USA; 24 Cardiovascular Health Research Unit, Departments of Medicine, Epidemiology, and Health Service, University of Washington, Seattle, WA, USA; 25 Group Health Research Institute, Group Health Cooperative, Seattle, WA, USA; 26 Departments of Epidemiology and Medicine, The Johns Hopkins School of Medicine and Public Health, Baltimore, MD, USA; 27 National Heart, Lung, and Blood Institute’s Framingham Heart Study, Framingham, MA, USA; 28 Cardiology Division, Department of Medicine, Massachusetts General Hospital, Harvard Medical School, Boston, MA, USA; 29 National Heart, Lung, and Blood Institute, Bethesda, MD, USA; 30 Department of Physiology and Biophysics, University of Mississippi, Jackson, MS, USA; 31 Laboratory of Epidemiology, Demography, and Biometry, Intramural Research Program, National Institute on Aging, National Institutes of Health, Bethesda, MD, USA; 32 Netherlands Genomics-Initiative-Sponsored Netherlands Consortium for Healthy Aging, Rotterdam, The Netherlands; 33 Department of Epidemiology, Erasmus University Medical Center, Rotterdam, The Netherlands; 34 Department of Biostatistics, Boston University School of Public Health, Boston, MA, USA; 35 Houston Institute of Molecular Medicine, University of Texas, Houston, TX, USA

**Keywords:** Atherosclerosis, Coronary artery calcium, Genetics, Meta-analysis, African-American

## Abstract

**Background:**

Coronary heart disease (CHD) is the major cause of death in the United States. Coronary artery calcification (CAC) scores are independent predictors of CHD. African Americans (AA) have higher rates of CHD but are less well-studied in genomic studies. We assembled the largest AA data resource currently available with measured CAC to identify associated genetic variants.

**Methods:**

We analyzed log transformed CAC quantity (ln(CAC + 1)), for association with ~2.5 million single nucleotide polymorphisms (SNPs) and performed an inverse-variance weighted meta-analysis on results for 5,823 AA from 8 studies. Heritability was calculated using family studies. The most significant SNPs among AAs were evaluated in European Ancestry (EA) CAC data; conversely, the significance of published SNPs for CAC/CHD in EA was queried within our AA meta-analysis.

**Results:**

Heritability of CAC was lower in AA (~30%) than previously reported for EA (~50%). No SNP reached genome wide significance (p < 5E-08). Of 67 SNPs with p < 1E-05 in AA there was no evidence of association in EA CAC data. Four SNPs in regions previously implicated in CAC/CHD (at 9p21 and *PHACTR1*) in EA reached nominal significance for CAC in AA, with concordant direction. Among AA, rs16905644 (p = 4.08E-05) had the strongest association in the 9p21 region.

**Conclusions:**

While we observed substantial heritability for CAC in AA, we failed to identify loci for CAC at genome-wide significant levels despite having adequate power to detect alleles with moderate to large effects. Although suggestive signals in AA were apparent at 9p21 and additional CAC and CAD EA loci, overall the data suggest that even larger samples and an ethnic specific focus will be required for GWAS discoveries for CAC in AA populations.

## Background

Atherosclerotic coronary heart disease (CHD) is a complex heritable condition and the major cause of death in the United States [1]. Recent meta-analyses of genome wide association studies (GWAS) in individuals of European Ancestry (EA) have identified single nucleotide polymorphisms (SNPs) at over 30 independent regions associated with coronary artery disease (CAD) and myocardial infarction (MI) [2-6]; however, these loci explain less than 10% of the heritability of the disease in EA. Although the burden of CHD is higher in African Americans (AA) than in EA [7-10], there are few contemporary genetic studies of CHD phenotypes in AA populations [11,12]. Moreover, adequately powered CHD GWAS in AA are lacking, with studies performed to date failing to identify any loci approaching genome wide significance [11]. The strongest loci for CAD/MI in EA GWAS [2-6], including the 9p21 locus, have shown inconsistent signals in small studies of AA [11] likely due to limited power and differences in linkage disequilibrium structure among the populations. Candidate gene studies of CHD in AA, however, have identified causal mutations that are private to AA populations [13].

One strategy for identifying genetic factors underlying susceptibility to CHD is to examine measures of subclinical atherosclerosis. Subclinical traits, such as coronary artery calcification (CAC), provide quantitative measures with reduced heterogeneity compared to presence or absence of clinical disease. CAC quantity is associated with traditional and novel CHD risk factors, is directly related to the burden of coronary atherosclerosis on angiography as well as autopsy, and also predicts incident CHD events after controlling for risk factors [14-17]. CAC is heritable in populations of EA [18-21] with estimates ranging from 40-60%. O’Donnell et al. [22] recently published the first large GWAS results (n = 9,992) of CAC in EAs which identified 49 SNPs in two distinct regions, 9p21 and the *PHACTR1* gene on chromosome 6, surpassing genome wide significance (p < 5E-08). Several of these SNPs were previously identified in EA GWAS of CAD/MI [2-6] providing support for CAC as a useful phenotype for discovery of novel CHD genes [22]. The lower prevalence of CAC in AA as compared to other ethnic groups, particularly persons of EA [23-25], might suggest, however, that their excess CHD rates may be attributed to differences in hypertension, diabetes, access to care, socioeconomic status or other CHD risk factors with limited influence on CAC [23,26].

Here, we present the largest AA GWAS of CAC, including 5,823 AA individuals. Using meta-analysis, we interrogated the largest AA CAC dataset available to date with genome wide SNP genotypes obtained as part of study-specific projects or through the National Heart Lung and Blood Institute (NHLBI) Candidate gene Association Resource (CARe) [27]. Our aims were to estimate the heritability of CAC in AA family samples, to perform a meta-analysis of GWAS results in an attempt to discover novel associations, and to assess the significance of genetic variants previously reported in subjects of EA.

## Methods

### Ethics statement

Each study obtained approval from their respective institutional review board and the ethics committee of each participating institution, including the University of Alabama at Birmingham, Washington University, University of Mississippi Medical Center, University of Minnesota, Northwestern University, Kaiser Permanente (Oakland, CA), University of Washington, Columbia University, Johns Hopkins School of Medicine, UCLA School of Medicine, Wake Forest University School of Medicine, University of Michigan Health Sciences and Behavioral Sciences, and the University of Pennsylvania. All participants gave written informed consent in accordance with institutional requirements and the principles expressed in the Declaration of Helsinki.

### Cohorts and CAC measurement

Eight cohorts (total N of 5,823) of AA participants (Additional file 1: Supplemental Methods) with measures of CAC participated in the meta-analysis (Table 1, Family Heart Study (FamHS), n = 596; Jackson Heart Study (JHS), n = 1,388 (comprised of JHS *de novo* recruited sample “JHS”, n = 1066 and a JHS sample previously enrolled in Atherosclerosis Risk in Communities (ARIC) study (“JHS-ARIC”), n = 322); Coronary Artery Risk Development In Young Adults (CARDIA), n = 671; Multi-Ethnic Study of Atherosclerosis (MESA), n = 1646; MESA Family/Air, n = 934; Genetic Study of Atherosclerosis Risk (GeneSTAR), n = 272; and Genetic Epidemiology Network of Arteriopathy (GENOA), n = 316;). Four of these (JHS, JHS-ARIC, CARDIA, and MESA) were genotyped through the NHLBI CARe [27], while FamHS, MESA Family/Air, GeneSTAR, and GENOA subjects were genotyped separately through funding from NHLBI. Given the low prevalence of CAC in younger individuals, participants were excluded if they were ≤ 35 years old. Participants were also excluded if they did not consent to genetic research or if genotype information did not meet cohort-specific quality-control standards (Additional file 1: Table S1 and Supplemental Methods). The definition of cardiovascular risk factors in each cohort is provided in the supplement. Participants provided written informed consent and protocols were approved by local institutional review boards.

**Table 1:** **Participant characteristics of eight participating African**-**American cohorts**

		**CARe cohorts**						
	**FamHS**	**JHS†**	CARDIA*	**JHS**-**ARIC†**	**MESA**	**MESA Family/Air**	**GeneSTAR**	**GENOA**
N analyzed	596	1066	671	322	1646	934	272	316
CAC score > 0, n (%)	330 (55.4%)	419 (39.3%)	108 (16.1%)	224 (69.6%)	726 (44.1%)	388 (41.8%)	111 (40.8%)	214 (67.7%)
CAC score mean	175.8	108.8	21.7	267.4	127.7	123.6	49.1	252.7
CAC score, median (Q1, Q3)	0.8 (0, 73.3)	0 (0, 37.8)	0 (0,0)	41.5 (0, 260.7)	0 (0, 53.2)	0 (0, 40.2)	0 (0, 16.0)	37.4 (0, 266.6)
CAC Heritability (SE)	0.33 (0.10)	0.47 (0.17)	-	-	-	0.31 (0.08)‡	0 (n/a)	0.26 (0.16)
Age, mean (range)	54.1 (36–83)	51.1 (36–90)	44.5 (37–54)	65.1 (57–80)	62.2 (45–84)	58.0 (39–91)	51.2 (36–64)	69.6 (56–85.5)
Sex, n (% male)	202 (33.9%)	408 (38.3%)	244 (36.4%)	87 (27%)	745(45.3%)	375 (40.4%)	92 (33.8%)	86 (27.2%)
Current Smoker, n (%)	144 (24.2%)	123 (11.5%)	157 (23.5%)	30 (9.3%)	297 (18%)	190 (20.5%)	75 (27.6%)	27 (8.5%)
Diabetic, n (%)	149 (25.4%)	145 (13.6%)	69 (10.3%)	77 (23.9%)	263 (16%)	137 (14.8%)	50 (18.4%)	105 (33.2%)
Hypertension, n (%)§	458 (76.9%)	607 (56.9%)	229 (34.1%)	243 (75.5%)	981 (59.6%)	561 (60.4%)	186 (68.4%)	263 (83.2%)
Statin Users, n (%)§	60 (10.1%)	95 (8.9%)	14 (2.1%)	53 (16.5%)	241 (14.6%)	193 (20.8%)	58 (21.3%)	-
Prevalent CHD, n (%)	67 (11.2%)	46 (4.3%)	13 (1.9%)	26 (8.1%)	0	46 (4.%)	0	7 (2.2%)

*CARDIA: Year 20 data; hypertension by self-report.

† JHS CAC data comprised of the JHS *de novo* recruited sample “JHS” (n = 1066) and the JHS sample previously enrolled in the ARIC study, denoted “JHS-ARIC” (n = 322). CAC data were collected in all JHS participants (JHS and JHS-ARIC) through JHS NHLBI funding. The JHS-de novo recruited sample was genotyped as a batch via the CARe study at the Broad Institute. Genotyping of all AA ARIC participants also was performed as a separate batch via the CARe study at the Broad Institute. The recommendation from the CARe study analysis committee was to analyze the” JHS” and “JHS-ARIC” individuals separately because QC of JHS and ARIC genotype data was not 100% identical.

‡MESA Family/Air: heritability based on n = 882 in families from participants of MESA Family.

§GeneSTAR: hypertension = average of 3 measures ≥ 140/90 mmHg and/or current use of antihypertensive medication; GENOA: Self-reported hypertension. Statin use data was not available; MESA Family/Air: hypertension if diastolic > =90 or systolic > =140; or self-reported high blood pressure and on meds for hypertension; FamHS, CARDIA, MESA, JHS: hypertension = measure ≥140/90 mm Hg and/or current use of antihypertensive medication.

All studies assessed CAC using computed tomography (CT, performed either by electron beam or multi-detector CT) imaging methods (Additional file 1: Supplemental Methods). Scans were interpreted at the corresponding sites of the independent studies but all investigators applied standardized methods using published software and reading algorithms [28-30]. Calcified plaque was quantified by the Agatston method [31] and the total calcium score, summing over the individual coronary arteries (i.e. left main, left anterior descending, circumflex, and right coronary arteries), was used in these analyses. Each study performed quality control in obtaining CAC measurements. In order to maintain comparability to published results for samples of European descent [22], we used the identical phenotypic transformation, ln(CAC + 1), in our primary analysis. In secondary analyses, we assessed CAC dichotomously (present/absent), ln(CAC) for those with CAC >0 and ln(CAC + 1) exclusively in the subset of older participants (men ≥ 50 and women ≥ 60). These secondary analyses produced similar results to the primary analysis and therefore these data are not presented.

### Power analysis

We estimated the power of our accumulated study sample using the software QUANTO [32,33]. We specified a quantitative outcome with an effective number of independent subjects (N_eff_ = 5,186, N_total_ = 5,823) accounting for the fact that several of the participating studies have family data, and gene only effect options, inputting sample size, estimated mean and standard deviation of ln(CAC + 1), and assuming an additive genetic model. This approach also assumes that meta-analysis is equivalent to pooled analysis. We also varied the allelic frequency from 0.01 to 0.4, and assessed three significance thresholds 0.05, 1E-05, and 5E-08 (two-sided). The effect size was characterized as r^2^, the proportion of phenotypic variance attributable to the SNP, which is a function of both allele frequency and the distance between genotype-specific means.

### Genotyping data and quality control

The CARe genotyping center at the Broad Institute (for JHS, JHS-ARIC, CARDIA, and MESA) or each individual study (for FamHS, MESA Family/Air, GeneSTAR, and GENOA,) was responsible for quality control for the genotypes and imputation (details in Additional file 1: Supplemental Methods and summarized in Additional file 1: Table S1). All studies used MaCH [34] (http://www.sph.umich.edu/csg/abecasis/MaCH/) for imputation except MESA Family/Air which used Impute [35] software (http://mathgen.stats.ox.ac.uk/impute/impute.html). We report results for SNPs with coded allele frequency (CAF) between 1-99%. More stringent CAF filters were used for MESA Family/Air (5% ≤ CAF ≤ 95%) and CARDIA (10% ≤ CAF ≤ 90%) due to small sample size, young mean age, and high prevalence of zero CAC which resulted in higher rates of Type I errors for SNPs with CAF less than 5 or 10% respectively; with these more stringent filters, the quantile-quantile (QQ) plots showed an acceptable fit (Additional file 2: Figure S1). SNPs with a Hardy-Weinberg equilibrium (HWE) test with p <1E-06 were excluded, as were SNPs with a call rate <0.95 or SNPs with an imputation quality metric (r^2^) of less than 0.50. In each study, hybrid datasets were created for analysis by substituting measured for imputed genotypes when available (Additional file 1: Supplemental Methods).

### Heritability calculation

Five of the participating studies have family data (FamHS, JHS, MESA Family/Air (family component), GeneSTAR, GENOA) and a variance components model was used to obtain maximum likelihood estimates of polygenic heritability for the age, age^2^, sex, and principal components (only those deemed necessary for each study to characterize population stratification, Additional file 1: Table S1, as estimated by EIGENSTRAT [36]) adjusted residuals of ln(CAC + 1) using the Sequential Oligogenic Linkage Analysis Routines (SOLAR) [37] software package.

### Cohort-specific analyses

For each measured or imputed SNP, each cohort provided estimated regression coefficients and standard errors (SE), the identity of the coded allele, its frequency (CAF), and p for a linear regression model of ln(CAC + 1) on allelic dosage for each SNP, using an additive genetic model. Each cohort adjusted the analysis for the effects of age, age^2^, sex, age*sex, age^2^*sex, CT scanner as needed, study site as needed, and the principal components deemed necessary for their study to characterize population stratification estimated by EIGENSTRAT [36] (Additional file 1: Table S1). A linear mixed effects model or Generalized Estimating Equation was used to account for correlation among participants in families.

### Meta-analyses

An inverse variance-weighted meta-analysis with fixed effects was used to estimate summary effects (METAL software, http://www.sph.umich.edu/csg/abecasis/metal/index.html) for the association of allelic dosage at each SNP with CAC (n = 5,823). Meta-analyses were performed independently at two sites (Washington University and University of Pennsylvania) for quality assurance and the results were concordant. Heterogeneity among studies was assessed using a χ^2^ test, and there was no significant heterogeneity for CAC quantity in our main results. We considered genome-wide significance as p < 5E-08, suggestive significance as p < 1E-05, and nominal significance as p ≤ 0.05. All tests were two-sided. SNPs reaching suggestive significance were assessed for their association with CAC in the EA CAC GWAS [22]. Conversely, we assessed the significance in our AA CAC meta-analysis data of EA CAC GWAS SNPs, including a close interrogation of both the genome wide significant loci for CAC in EA data [22]: the 9p21 region [11] and *PHACTR1* locus.

## Results

### Sample characteristics and heritability

A description of each cohort is provided in the Additional file 1: Supplemental Methods. Demographic and selected risk factor characteristics of the 5,823 study participants are described in Table 1 by cohort. Gender distribution was similar across studies. There was, however, variation in the age range across cohorts, e.g., CARDIA (37–54 yrs) vs. GENOA (56–86 yrs), as well as some risk factors such as a higher prevalence of diabetes and hypertension in GENOA and more current smokers in GeneSTAR. Additional file 1: Supplemental Methods and Table S1 summarizes the cohort-specific genotyping, imputation, and quality control procedures, including the number of SNPs used in cohort-specific analyses (ranging from 1.9 million to 2.7 million).

### Power analysis

**Figure 1:**
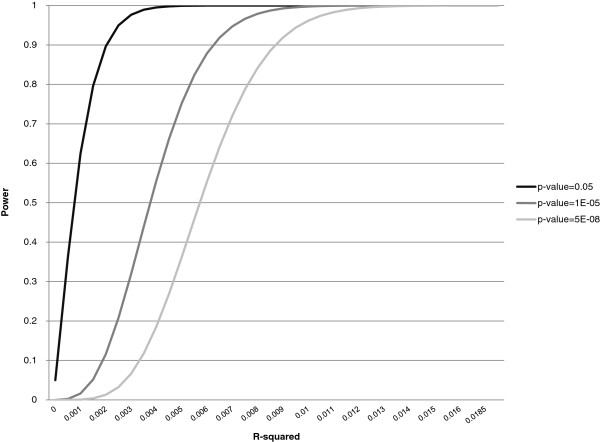
**Power curves.** Power curves calculated using QUANTO [32,33] software, as described in the text. In brief, we specified a quantitative outcome, assumed an additive genetic model and used an effective sample size of 5,186 with the estimated mean and standard deviation of ln(CAC + 1). Allelic frequency variation did not affect the power estimates. We characterized the effect size as r^2^.

We estimated that our sample size of 5,823 represented an effective sample size of 5,186 taking account of the non-independent observations in the family studies. With this sample size, we had 80% power to detect a genetic variant accounting for as little as 0.77% of the variance in CAC quantity with genome-wide significance and as little as 0.15% with nominal significance (p <0.05) (Figure 1). Our sample had >80% power to detect a variant with comparable effect size to that in 9p21 associated with CAC in EA (effect size = 0.009, or 0.9%; unpublished data, 2012). Thus, our AA CAC study was adequately powered to detect effect sizes comparable to those observed for the top associated SNPs in the EA GWAS of CAC. However lower allele frequencies in African descent samples could lead to a lower overall effect size, even if the effect of the allele is the same as in European samples.

### Heritability analysis

The heritability of CAC scores was estimated in each of the five family studies (Table 1). The range of heritability across these AA family samples (0-47%) tended to be lower than those reported among EA CAC studies (40-60%) [18-21]. The AA estimate in GeneSTAR (0%) may be sensitive to the small sample size and lower prevalence of CAC relative to EA families, but it is consistent with a lower heritability of CAC in AA compared to EA. Setting aside the GeneSTAR study, we estimate the heritability of CAC in AA to be ~30% which still suggests lower heritability in AA compared to EA.

### Meta-analysis findings

**Figure 2:**
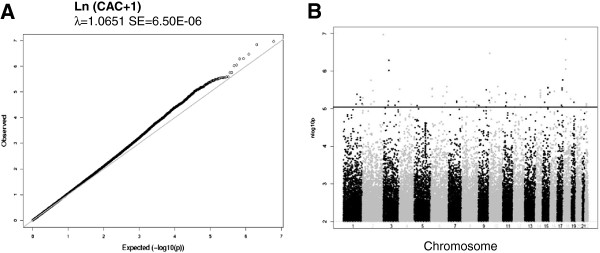
**Quantile-quantile and Manhattan plots of AA CAC GWAS results. A)** Quantile-quantile plot for the Meta-analysis of CAC. **B)** Manhattan plot for the meta-analysis of SNPs associated with CAC. No SNPs reach genome-wide significance, however SNPs above the blue line indicate the suggestive significance level of p < 1.0E-05.

The quantile-quantile (QQ) plot for the combined AA GWA meta-analysis is shown in Figure 2A. Principal components were used in each cohort-specific analysis and lambda values were between 0.96 and 1.1, thus no genomic control correction was applied to our results. QQ plots for cohort-specific GWA analyses are shown in Additional file 2: Figure S1. As summarized in Additional file 1: Table S2 and shown on the Manhattan plot in Figure 2B, our meta-analysis yielded no genome-wide significant results and there was limited evidence of clustering of top SNPs at a single chromosomal location. The SNP with the smallest p value was rs749924 on chromosome 2, p = 1.07E-07. We focused on SNPs with p < 1E-05, which identified 67 SNPs with suggestive statistical evidence of association with CAC (Additional file 1: Table S2). These 67 SNPs represent 45 potentially independent signals (using SimpleM [38]) and included none of the loci associated with CAC in EA [22]. The secondary analyses produced similar results to the primary analysis and therefore these results are not presented.

### Evidence for association of suggestive AA SNPs in data from individuals of European ancestry

We interrogated our 67 most significant SNPs (original or best proxy, using the Broad Institutes SNP Annotation and Proxy Search (SNAP) website; http://www.broadinstitute.org/mpg/snap/ldsearch.php) for their association with CAC in the CHARGE meta-analysis of individuals of EA [22]. Of these 67 SNPs with suggestive CAC association (p <1E-05) in AA, three (one each in *SOX9*, *PRKCA*, and *NRG1)* reached nominal significance (p ≤ 0.05) for association with ln(CAC + 1) in CHARGE EA data (Additional file 1: Table S2). However, 2 of the 3 SNPs (in *SOX9* and *PRKCA)* showed association in an inconsistent allelic direction of effect and while rs1462872 in *NRG1* demonstrated an association in the same direction, the p value in EA was 0.05. Due to multiple testing comparisons and given this single isolated hit in a relatively large gene, we suspect this association is a false positive result, suggesting that there was no meaningful evidence for replication of these AA SNP signals in the CHARGE EA sample.

### Interrogation of reported EA CHD GWAS signals in this AA CAC GWAS

We amassed EA CHD GWAS signals (for CAC and for CAD/MI e.g., with specific focus on the 9p21 region and *PHACTR1* locus) to assess their signals in the AA CAC GWAS results.

### EA CAC GWAS signals in African Americans

Recently, O’Donnell and colleagues [22] identified several SNPs that were genome-wide significant in two regions in a GWAS of CAC in EA. These regions, 9p21 and *PHACTR1* on 6p24.1, are also associated with CAD/MI [2-6]. We queried these SNPs (or their proxies, identified using SNAP) for CAC association in AAs. Of 49 SNPs reaching genome-wide significance for CAC in EAs, 44 (89.8%) had the same direction of effects on CAC in AAs. Six of these directionally consistent SNPs also had a p-value <0.1 in AA, which we consider nominally significant for a one-sided test suggesting modest enrichment for EA CAC alleles within our AA sample (EA CAC GWAS significant SNPs with replication p values <0.10 in AA CAC meta-analysis shown in Table 2, results for all previously reported suggestive SNPs (p < 1E-05) in EA CAC have AA CAC results reported in Additional file 1: Table S3).

**Table 2:** **CHARGe European ancestry CAC meta**-**analysis **[22]**SNP top hits with nominal significance in the African American CAC meta-analysis**

					**EA CHARGE meta-analysis results (n = 9,992)**						**AA CAC meta-analysis look-up (n = 5,823)**					
**SNP**	**Chrom**	**Position**	**Closest gene**	**Role†**	**Coded allele**	**Coded allele freq**	**Effect**	**SE‡**	**p‡**	**Direction of point estimate for the association §**	**Coded allele**	**Coded allele freq**	**Effect**	**SE**	**p**	**Direction of point estimate for the association §**
rs3218020	9	21997872	*CDKN2A*		A	0.34	0.19	0.03	2.53E-09	+ + + + +	A	0.15	0.15	0.05	0.002	+ + + - + + - -
rs1537375	9	22116071	*CDKN2B*		T	0.50	−0.24	0.03	5.06E-16	- - - - -	T	0.33	−0.08	0.04	0.03	+ - - - - - - +
rs9349379	6	12903957	*PHACTR1*	intron	A	0.59	−0.21	0.03	2.65E-11	- - - + -	A	0.90	−0.14	0.08	0.07	- + ? + - - + +
rs4977575	9	22124744	*CDKN2B*		C	0.52	−0.27	0.03	9.93E-19	- - - - -	C	0.12	−0.09	0.05	0.08	- - - + - - - +
rs1333042	9	22103813	*CDKN2B*		A	0.51	−0.24	0.03	4.54E-16	- - - - -	A	0.12	−0.09	0.05	0.09	+ - - - + - - -
rs10511701	9	22112599	*CDKN2B*		T	0.49	−0.24	0.03	4.48E-16	- - - - -	T	0.28	−0.07	0.04	0.09	+ - - - + - - +

†If no role indicated, then is outside known gene boundaries.

‡SE: Standard error; p: p-value.

§Order of studies: for EA CHARGE: Age, Gene/Environment Susceptibility Study—Reykjavik (AGES), Rotterdam Study-II, Framingham Heart Study, GENOA, Rotterdam Study-I; and for AA CAC: FamHS, JHS, CARDIA, JHS-ARIC, MESA, MESA Family/Air, GeneSTAR, GENOA. GWAS results from each study were completed independently, thus data availability varied by study depending on study specific imputation quality and genotyping quality control for each SNP. Therefore not all studies had results for all SNPs.

### EA CAD GWAS signals in African Americans

Since CAC is a strong indicator of risk for cardiovascular endpoints, we interrogated top genome-wide significant SNPs (or their proxies) from large GWAS meta-analyses in EA for CAD/MI [2-6] in our AA CAC results. Of 34 SNPs at previously identified loci for CAD/MI in EA [5], 25 (73.5%) had the same direction of effects on CAC in AAs but none, including the top EA 9p21 SNP, were nominally significant in our AA data (Additional file 1: Table S4).

### Signals from the 9p21 fine mapping regions defined for EA and AA

**Figure 3:**
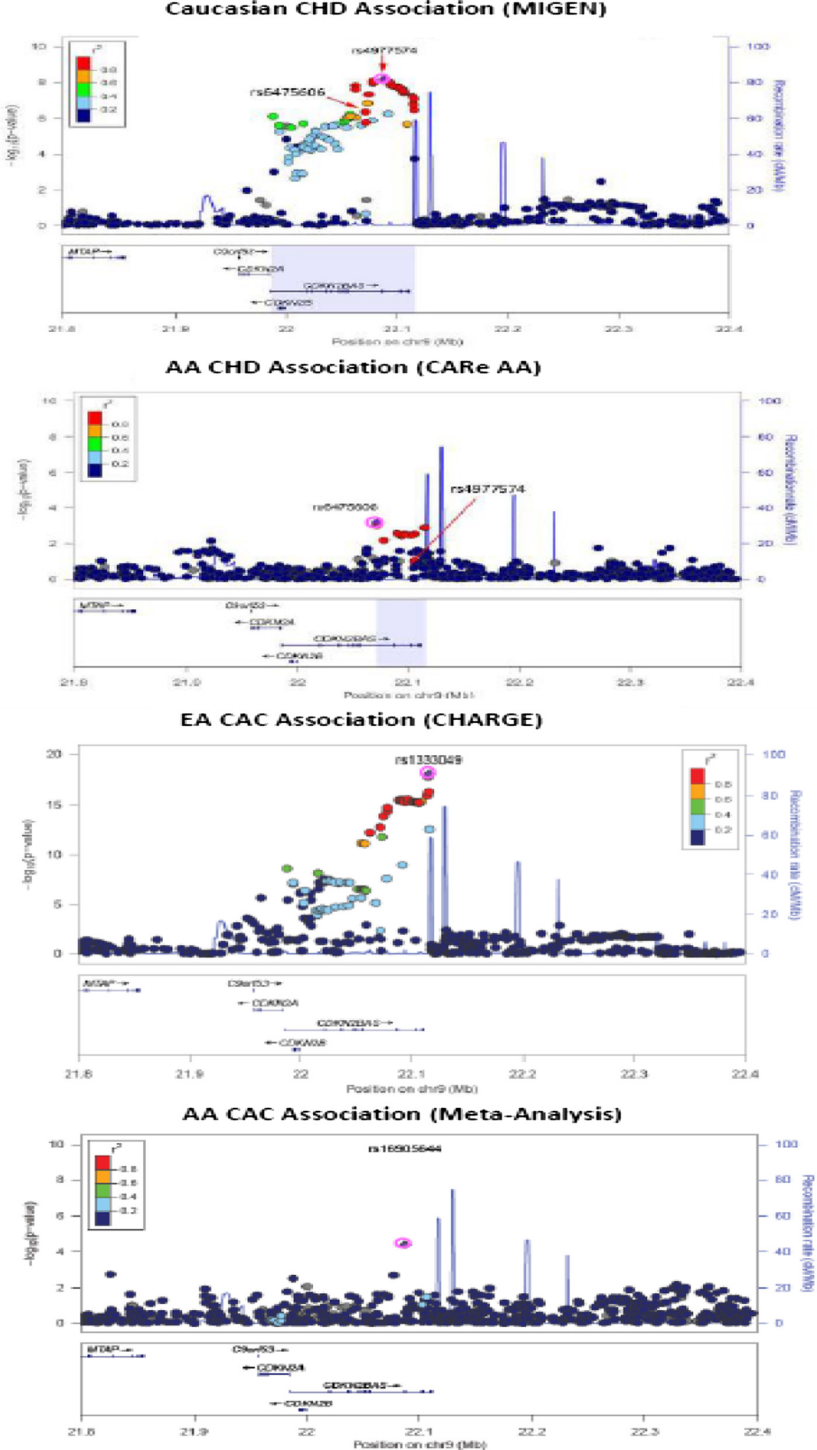
**LocusZoom plots of the 9p21 region.** Fine mapping of the 9p21 region using LOCUS ZOOM [39]. The top plot uses data from MIGen [3] for the Caucasian CHD associations (lead EA SNP is rs4977574 on right and lead AA SNP from CARe CHD is rs6475606 on left). The second plot demonstrates the AA CHD associations, highlighting the same two SNPs as the first graph [11]. The third plot depicts the EA CAC associations [22] and its lead SNP, rs1333049. The final plot depicts our AA CAC associations with lead SNP rs16905644 (AA CAC meta-analysis associations for all these SNPs are in Additional file 1: Supplemental Table S5). In all plots, the region associated with EA is broader than the region associated with AAs and the top results for AA CAC are in the same region as that associated with CHD in AA [11].

We queried the association in AA of 166 SNPs within the EA region for CAD and CAC in the 9p21 region, and we identified 24 SNPs with nominal evidence for association (p ≤ 0.05). The peak AA CAC association mapped to a different SNP than those reported in other populations and to a smaller linkage disequilibrium (LD) region reported in the CARe fine mapping effort for CHD in AA [11], with the strongest association at rs16905644 (CAF 0.11, p = 4.07E-05; Bonferroni correction for 166 SNPs tested, p = 0.0068; Figure 3). Overall, ten of these 24 nominally associated SNPs localized within this smaller AA region, but fourteen lay outside this AA region but still within the larger EA region (Additional file 1: Table S5). However, neither the strongest 9p21 EA SNPs for CAC (rs1333049) or CAD (rs4977574) in EA nor rs6475606 or rs3217989 at 9p21, recently reported to be associated with CHD in AA [40] were among these nominally significant signals (Figure 3 and Additional file 1: Table S5).

### Signals from the *PHACTR1* locus for CAC and CAD in EA

We queried the association in AA of approximately 20 SNPs within the EA region for CAD and CAC in *PHACTR1*[3,5,22] and we identified no SNP with even nominal evidence for association (p ≤ 0.05). However, of these 20 SNPs, the lead SNP from the EA CAC analysis, rs9349379, had a p-value of 0.09 in the AA CAC analysis with similar direction of effect, but the allele frequency was markedly different (CAF EA = 0.59; CAF AA = 0.90). We further examined all SNPs within *PHACTR1* (12.7-13.3 megabases) in the AA CAC GWAS, including a comparison of the LD structure of this region in EA and AA populations*.* The most significant *PHACTR1* association in our AA CAC GWAS was an intronic SNP, rs7768030 (p = 0.004, Additional file 1: Table S7 for all results), which is located 80.9 kb from the lead EA CAC SNP, and even further from the lead EA MI SNP, rs12526453 [3,5]. The rs7768030 SNP is not in long range LD with these two EA SNPs, as evidenced from data from HapMap (Additional file 1: Table S8). Indeed, based on HapMap, there are marked differences between AA and EA LD patterns in this region (Additional file 3: Figure S2); we present the regional plots in EA and AA for the FamHS CAC data around these top hits in *PHACTR1* (rs9349379 and rs7768030, respectively; Additional file 4: Figure S3). Overall, based on these analyses, there may be a signal for CAC in AA at *PHACTR1* but in a different region of this large gene than that observed for CAC and CAD/MI in EA.

## Discussion

Despite higher rates of CHD in AA [8], adequately powered genomic studies in this population are lacking. We leveraged data from almost all available US-based studies of CAC in AAs and performed a meta-analysis of their GWAS results with the goal of identifying novel loci using this validated marker of subclinical coronary atherosclerosis and predictor of CHD. Using this largest collection of CAC in AAs, we observed substantial heritability for CAC in AA, albeit lower than that reported for EA [18-21], and we failed to reveal loci for CAC at genome-wide significant levels despite having adequate power to detect common alleles with moderate to large effect sizes. We found no credible support for our top AA GWAS meta-analysis findings in the EA CHARGE CAC GWAS [22]. We note that we were not able to perform the ideal replication, which would be a separate analysis of CAC in an independent AA sample. The majority of genome wide significant loci identified for CAC in EA (49 SNPs in 2 gene regions) [22] and CAD/MI in EA (34 SNPs at 34 distinct regions) [2-6] had the same direction of allelic effect in our AA GWAS data but only four reached nominal significance (p ≤ 0.05) with similar direction of effect. Because inconsistent findings between AA and EA might represent genetic differences between the populations, we interrogated SNP associations across the 9p21 region [5,22,41] which has documented distinct LD structure among individuals of EA and individuals of AA [11] and SNP associations across *PHACTR1*[22]. Indeed, in the 9p21 region we detected a SNP with suggestive evidence of an association with CAC in AA (rs16905644, p = 4.07E-05) but within the smaller region of LD in AA. At the *PHACTR1* locus, there were distinct patterns of LD and allele frequencies in Africans compared to Europeans with a weak association of *PHACTR1* SNPs with CAC in AA at some distance from that for CAC and CAD/MI in EA, but still within the recognized gene boundaries. Although this is suggestive of a separate signal in *PHACTR1* among AA, further fine mapping and interrogation of rare variants is required to determine if *PHACTR1* is a *bona fide* locus for CAC in AA populations. Despite modest suggestive findings in AA at 9p21 and some other CAC and CAD EA loci, these data suggest that even larger samples than we analyzed with race specific fine mapping will be required for CAC GWAS discoveries in AA populations.

The lack of novel or strong confirmatory signals in our analysis of AA samples may be attributable to several factors. Although we had adequate power to detect effects similar to those observed for the strongest loci in EA, it is possible that the actual marginal genetic effects in AAs are smaller. It seems unlikely, however, that this is the whole explanation for the apparent lack of overlap between EA and AA signals for CAC. One factor that may attenuate signals in AAs is the smaller haplotype blocks (decreased levels and range of LD across the genome) as compared with EA populations [5,22,41]. As a consequence, it is possible that the imputed reference panel of SNPs used in this study are inadequate tags of the AA genome with the consequence that we may miss functional SNP signals due to inadequate coverage. Lower LD between measured tag variants and unmeasured functional variants will result in a net decrease in effect size and, thus, lower power for detection. This concern can be addressed with denser, race-specific gene maps or sequencing in AA populations, but until then, we cannot verify the relevance of EA variants in AAs.

Another factor that may affect our ability to detect trait loci relates to racial differences in the heritability of the CAC trait. We estimated the heritability of CAC in several participating AA family studies, and found up to 47% of the CAC variance to be influenced by the genetic variation, with a lower bound estimate of zero for one study. Setting aside this study with 0% heritability, the heritability of CAC in AA is ~30% which is lower than that reported in EA samples (~50%) [18-21]. Heritability estimates may reflect population differences between AA and EA samples in known risk factors for CAC, but comparison between race, within studies (e.g., in FamHS 33% for AA vs. 45% for EA, North et al. [21]; in GeneSTAR 0% for AA vs. 27% for EA) reinforces the possibility of differences between AA and EA in the impact of genetic variation on CAC. This apparent lower level of CAC heritability in AAs could reflect a relatively greater importance of non-genetic factors and gene by environment interactions as compared to additive genetic effects [42].

It is also possible that different biological pathways or different genetic variants within the same pathways are at play resulting in genetic heterogeneity between European and African ancestral populations in the mechanisms leading to atherogenesis and CAC. This is borne out by the observation that greater CAC burden is associated with higher levels of European admixture in AA populations [43], suggesting that genetic variants specific to EA play a more important role in the development of CAC than those of African origin. Indeed, there are several lines of evidence suggesting distinct pathophysiology of CAC in AAs, including lower CAC scores despite greater risk factor burden and higher rates of CHD in AA samples [7,9,10,23,24,26,44-46]. Therefore, while some EA variants may play a role in atherosclerosis in AA, other distinct pathways may be important. In this case, validation in EA populations, as we attempted, would not be expected to succeed. Finally, environmental factors, either by themselves or interacting with genetic background, may have a more prominent role in CAC and atherosclerosis in AA than genetic effects. CAC scores do, however, predict CHD events in AA [16] samples suggesting that larger studies pursuing genetic discoveries using CAC in AA should provide some insights into mechanisms and risk of CHD in this population.

Our study has several strengths. First, it is the largest GWAS of any sub-clinical atherosclerosis trait in AAs. Second, it leverages data from nearly all AA cardiovascular cohort studies and represents a cross-section of the US AA population. An attribute of our study was the *a priori* planning such that GWAS datasets were analyzed using raw data from the cohorts in a pre-specified manner rather than a post-hoc combination of results, followed by attempted validation of our top findings in EA GWAS CAC datasets. Third, we used multiple family datasets to obtain heritability estimates of CAC in AA. Finally, although the results of this work are largely negative, it highlights the need to pursue additional genetic epidemiological studies of CHD in AA populations.

Our study also has several limitations. Although our sample was the largest GWAS of a sub-clinical atherosclerosis trait in AAs and powered for loci with comparable effect sizes to the strongest loci identified in EA GWAS, this study was underpowered to discover SNPs with small effects. We lacked a positive control genotype that could support the power of our study to detect expected genetic effects for CAD; *PCSK9* and *LPA* genotypes associated with CAD in AA were not genotyped and lacked proxy SNPs in our data. However, we did interrogate the well-documented 9p21 and *PHACTR1* regions, including the known different LD structure in the 9p21 region [5,22,41] as a potential positive control; indeed, this 9p21 analysis, using an appropriate ethnic-specific LD focus, did provide suggestive/nominal evidence for 9p21 locus effects on CAC in AA; and weak evidence for *PHACTR1* locus effects on CAC in AA.

## Conclusion

In summary, our results for the largest AA CAC GWAS amassed to date are remarkable in two respects: first, in the lack of support in EA data for the top signals arising from AA data and second, in the weak support for association of EA CAC [22] and CAD and MI loci [2-6] in our AA sample. Substantial biological differences in the genomic architecture of CAC, atherosclerosis and clinical CHD between AA and EA populations are likely.

## Abbreviations

CHD: Coronary heart disease; CAC: Coronary artery calcification; AA: African Americans; SNP: Single nucleotide polymorphism; EA: European ancestry; PHACTR1: Phosphatase and actin regulator 1; GWAS: Genome-wide association study; CAD: Coronary artery disease; MI: Myocardial infarction; NHLBI: National heart, lung, and blood institute; CARe: Candidate gene association resource; FamHS: Family heart study; JHS: Jackson heart study; ARIC: Atherosclerosis risk in communities study; JHS-ARIC: Jackson heart study-atherosclerosis risk in communities study; CARDIA: Coronary artery risk development in young adults; MESA: Multi-ethnic study of atherosclerosis; GeneSTAR: Genetic study of atherosclerosis risk; GENOA: Genetic epidemiology network of arteriopathy; CT: Computed tomography; CAF: Coded allele frequency; QQ: Quantile-quantile plot; HWE: Hardy-Weinberg equilibrium; SOLAR: Sequential oligogenic linkage analysis routines; SE: Standard error; SNAP: SNP annotation and proxy search; P: p-value; NRG1: Neuregulin 1; SOX9: SRY (sex determining region Y)-box 9; PRKCA: Protein kinase C, alpha; LD: Linkage disequilibrium; PCSK9: Proprotein convertase subtilisin/kexin type 9; LPA: Lipoprotein, Lp(a); n: Sample size; Q1: 25^th^ percentile; Q3: 75^th^ percentile; Chrom: Chromosome.

## Competing interests

The authors do not have any conflicts of interest, financial or otherwise.

## Authors’ contributions

NDW, LAL, THM, XG, SKG, SRH, MEG, DHO, MB, JJC, HAT, DAB, SD, DNP, JFP, BMP, DMB, MAP, WSP, CJO, JGW, TBH, MK, LAC, JIR, MF, LCB, PAP, IBB, and MPR conceived and designed the study. MKW, ML, LFB, KFK, APR, LRY, LQ, LAL, JFF, JH, TY, JAS, BGK, XG, QW, S-JH, BMP, DMB, MAP, WSP, CJO, JGW, TBH, LAC, MF, JIR, LCB, PAP, IBB, and MPR acquired the data. MKW, ML, LFB, KFK, APR, LRY, LQ, CCW, LAL, JFF, JH, TY, JAS, BGK, XG, QW, MB, JJC, S-JH, BMP, DMB, MAP, WSP, CJO, JGW, TBH, LAC, JIR, MF, LCB, and PAP analyzed the data. MKW, ML, LFB, KFK, APR, NDW, LRY, LQ, LAL, JFF, JH, THM, JAS, BGK, XG, QW, SKG, SRH, MEG, DHO, MB, JJC, HAT, DAB, SD, S-JH, DNP, JFP, BMP, DMB, MAP, WSP, CJO, JGW, TBH, MK, LAC, JIR, MF, LCB, PAP, IBB, and MPR interpreted the data. MKW, IBB, and MPR drafted the manuscript. All authors revised the manuscript for important intellectual content, read, and approved the final manuscript.

## Authors’ information

Ingrid B. Borecki and Muredach P. Reilly are senior authors.

## Pre-publication history

The pre-publication history for this paper can be accessed here:

http://www.biomedcentral.com/1471-2350/14/75/prepub

## Supplementary Material

Additional file 1 **Supplemental methods and Table S1.** Cohort-specific genotyping, imputation, and quality control procedures/criteria. **Table S2.** AA CAC meta-analysis SNP ‘top hits’ and their assessment in the CHARGe EA CAC meta-analysis [12]. **Table S3.** Assessment in African-Americans of SNPs previously associated with CAC in the CHARGe EA CAC Meta-Analysis [12]. **Table S4.** Assessment in African-Americans of loci previously associated with coronary artery disease. **Table S5.** SNP signals within EA and AA LD blocks at the 9p21 region as defined by CARe AA CHD GWAS*. **Table S6.** Participant characteristics of the CHARGE EA CAC sample [12]. **Table S7.** Assessment of SNP associations in PHACTR1 region in the AA CAC Meta-Analysis. **Table S8.** Attributes of top *PHACTR1* SNPs from O’Donnell^†^, CARDIoGRAM^¥^, MIGEN^§^, and AA CAC^‡^ obtained from HapMap. Click here for file

Additional file 2: Figure S1 Quantile-quantile plots of AA CAC GWAS results from each study. Click here for file

Additional file 3: Figure S2 Linkage disequilibrium plots from HapMap. A) For the CEPH population and B) For the YRI population, both for the region of *PHACTR1* from 12800 kb-13100 kb. Blue arrow points to lead EA CAC SNP from O’Donnell et al, rs9349379; purple arrow points to AA CAC meta-analysis lead SNP in *PHACTR1*, rs7768030; green arrow is rs2026458 from O’Donnell et al; orange arrow is rs12526453 from MIGEN and CardioGRAM. As depicted, there is vastly different LD structure between these populations and these SNPs are in different LD blocks. Click here for file

Additional file 4: Figure S3 Regional plots of association results for the region from 12.7 Mb – 13.3 Mb in *PHACTR1.* This uses A) EA CAC data from FamHS (in house data) and B) AA CAC meta-analysis results. There is little LD between the two top hits, rs9349379 in EA and rs7768030 in AA (purple diamonds in figure); however they may be tagging some common underlying functional variant that is not genotyped. Click here for file
