# Supplementary material for: Genetics of coronary artery calcification among African Americans, a meta-analysis
Source: BMC Med Genet. 2013 Jul 19;14:75. doi: 10.1186/1471-2350-14-75 (PMC3733595; doi:10.1186/1471-2350-14-75)
Supplement: Additional file 1 — Supplemental methods and Table S1. Cohort-specific genotyping, imputation, and quality control procedures/criteria. Table S2. AA CAC meta-analysis SNP ‘top hits’ and their assessment in the CHARGe EA CAC meta-analysis [12]. Table S3. Assessment in African-Americans of SNPs previously associated with CAC in the CHARGe EA CAC Meta-Analysis [12]. Table S4. Assessment in African-Americans of loci previously associated with coronary artery disease. Table S5. SNP signals within EA and AA LD blocks at the 9p21 region as defined by CARe AA CHD GWAS*. Table S6. Participant characteristics of the CHARGE EA CAC sample [12]. Table S7. Assessment of SNP associations in PHACTR1 region in the AA CAC Meta-Analysis. Table S8. Attributes of top PHACTR1 SNPs from O’Donnell†, CARDIoGRAM¥, MIGEN§, and AA CAC‡ obtained from HapMap. [file 1471-2350-14-75-S1.docx]

**Genetics of Coronary Artery Calcification among African Americans, a Meta-Analysis**

**First Author Surname:** Wojczynski

**Short Title:** AA CAC GWAS, supplement

Mary K. Wojczynski^1*^

*Corresponding Author

Email: mwojczynski@wustl.edu

Mingyao Li^2^

Email: [mingyao@mail.med.upenn.edu](mailto:mingyao@mail.med.upenn.edu)

Lawrence F. Bielak^3^

Email: [lfbielak@umich.edu](mailto:lfbielak@umich.edu)

Kathleen F. Kerr^4^

Email: [katiek@u.washington.edu](mailto:katiek@u.washington.edu)

Alex P. Reiner^5^

Email: [apreiner@u.washington.edu](mailto:apreiner@u.washington.edu)

Nathan D. Wong^6^

Email: [ndwong@uci.edu](mailto:ndwong@uci.edu)

Lisa R. Yanek^7^

Email: [lryanek@jhmi.edu](mailto:lryanek@jhmi.edu)

Liming Qu^2^

Email: [qul@mail.med.upenn.edu](mailto:qul@mail.med.upenn.edu)

Charles C. White^8^

Email: [ccwhite@bu.edu](mailto:ccwhite@bu.edu)

Leslie A. Lange^9^

Email: [leslie_lange@med.unc.edu](mailto:leslie_lange@med.unc.edu)

Jane F. Ferguson^10^

Email: [JFER@mail.med.upenn.edu](mailto:JFER@mail.med.upenn.edu)

Jing He^2^

Email: [jinghe@mail.med.upenn.edu](mailto:jinghe@mail.med.upenn.edu)

Taylor Young^11^

Email: [tyoung@broadinstitute.org](mailto:tyoung@broadinstitute.org)

Thomas H. Mosley^12^

Email: [tmosley@umc.edu](mailto:tmosley@umc.edu)

Jennifer A. Smith^3^

Email: [smjenn@umich.edu](mailto:smjenn@umich.edu)

Brian G. Kral^7^

Email: [bkral@jhmi.edu](mailto:bkral@jhmi.edu)

Xiuqing Guo^13^

Email: [Xguo@labiomed.org](mailto:Xguo@labiomed.org)

Quenna Wong^4^

Email: [qwong@u.washington.edu](mailto:qwong@u.washington.edu)

Santhi K. Ganesh^14^

Email: [sganesh@umich.edu](mailto:sganesh@umich.edu)

Susan R. Heckbert^5^

Email: [heckbert@u.washington.edu](mailto:heckbert@u.washington.edu)

Michael E. Griswold^15^

Email: [mgriswol@jhsph.edu](mailto:mgriswol@jhsph.edu)

Daniel H. O’Leary^16^

Email: [doleary@tuftsmedicalcenter.org](mailto:doleary@tuftsmedicalcenter.org)

Matthew Budoff^17^

Email: [mbudoff@labiomed.org](mailto:mbudoff@labiomed.org)

J. Jeffrey Carr^18^

Email: [Jcarr@wakehealth.edu](mailto:Jcarr@wakehealth.edu)

Herman A. Taylor, Jr.^19,20^

Email: [htaylor@umc.edu](mailto:htaylor@umc.edu)

David A. Bluemke^21^

Email: [bluemked@mail.nih.gov](mailto:bluemked@mail.nih.gov)

Serkalem Demissie^8^

Email: [demissie@bu.edu](mailto:demissie@bu.edu)

Shih-Jen Hwang^22^

Email: [hwangs2@nhlbi.nih.gov](mailto:hwangs2@nhlbi.nih.gov)

Dina N. Paltoo^23^

Email: [dina.paltoo@nih.gov](mailto:dina.paltoo@nih.gov)

Joseph F. Polak^16^

Email: [jpolak@tuftsmedicalcenter.org](mailto:jpolak@tuftsmedicalcenter.org)

Bruce M. Psaty^24,25^

Email: [psaty@u.washington.edu](mailto:psaty@u.washington.edu)

Diane M. Becker^7^

Email: [DBecker607@aol.com](mailto:DBecker607@aol.com)

Michael A. Province^1^

Email: [mprovince@wustl.edu](mailto:mprovince@wustl.edu)

Wendy S. Post^26^

Email: [wpost@jhmi.edu](mailto:wpost@jhmi.edu)

Christopher J. O’Donnell^27,28,29^

Email: [codonnell@nih.gov](mailto:codonnell@nih.gov)

James G. Wilson^30^

Email: [jgwilson2@umc.edu](mailto:jgwilson2@umc.edu)

Tamara B. Harris^31^

Email: [harrista@nia.nih.gov](mailto:harrista@nia.nih.gov)

Maryam Kavousi^32,33^

Email: [m.kavousi@erasmusmc.nl](mailto:m.kavousi@erasmusmc.nl)

L. Adrienne Cupples^27,34^

Email: [Adrienne@bu.edu](mailto:Adrienne@bu.edu)

Jerome I. Rotter^13^

Email: [Jrotter@labiomed.org](mailto:Jrotter@labiomed.org)

Myriam Fornage^35^

Email: [Myriam.Fornage@uth.tmc.edu](mailto:Myriam.Fornage@uth.tmc.edu)

Lewis C. Becker^7^

Email: [lbecker@jhmi.edu](mailto:lbecker@jhmi.edu)

Patricia A. Peyser^3^

Email: [ppeyser@umich.edu](mailto:ppeyser@umich.edu)

Ingrid B. Borecki^1*†^

*Co-Corresponding Author

Email: [iborecki@wustl.edu](mailto:iborecki@wustl.edu)

Muredach P. Reilly^10*†^

*Co-Corresponding Author

Email: Muredach@mail.med.upenn.edu

^1^ Department of Genetics, Division of Statistical Genomics, Washington University School of Medicine, St. Louis, MO, USA.

^2^ Biostatistics and Epidemiology, University of Pennsylvania, Philadelphia, PA, USA.

^3^ Department of Epidemiology, University of Michigan School of Public Health, Ann Arbor, MI, USA.

^4^ Department of Biostatistics, University of Washington, Seattle, WA, USA.

^5^ Department of Epidemiology, University of Washington, Seattle, WA, USA.

^6^ Heart Disease Prevention Program, University of California, Irvine, CA, USA.

^7^ Department of Medicine, The Johns Hopkins University School of Medicine, Baltimore, MD, USA.

^8^ Department of Biostatistics, Boston University School of Public Health, Boston, MA, USA.

^9^ Department of Genetics, University of North Carolina, Chapel Hill, NC, USA.

^10^ The Cardiovascular Institute and Department of Medicine, Perelman School of Medicine, University of Pennsylvania, Philadelphia, PA, USA.

^11^ Program in Medical and Population Genetics, Broad Institute, Cambridge, MA, USA.

^12^ Department of Medicine, University of Mississippi Medical Center, Jackson, MS, USA.

^13^ Institute for Translational Genomics and Population Sciences, Los Angeles Biomedical Research Institute at Harbor-UCLA Medical Center, Torrance, CA, USA.

^14^ Division of Cardiovascular Medicine, Department of Internal Medicine, University of Michigan, Ann Arbor, MI, USA.

^15^ Center of Biostatistics and Bioinformatics, University of Mississippi Medical Center, Jackson, MS, USA.

^16^ Department of Radiology, Tufts University School of Medicine, Boston, MA, USA.

^17^ University of California Los Angeles School of Medicine, Los Angeles, CA, USA.

^18^ Department of Radiology, Wake Forest University School of Medicine, Winston-Salem, NC, USA.

^19^ Jackson State University, Tougaloo College, Jackson, MS, USA.

^20^ University of Mississippi Medical Center, Jackson, MS, USA.

^21^ Radiology and Imaging Sciences, National Institutes of Health Clinical Center, Bethesda, MD, USA.

^22^ National Heart, Lung, and Blood Institute's Framingham Heart Study and the Center for Population Studies, Framingham, MA, USA.

^23^ Advanced Technologies and Surgery Branch, Division of Cardiovascular Sciences, National Heart, Lung, and Blood Institute, National Institutes of Health, Bethesda, MD, USA.

^24^ Cardiovascular Health Research Unit, Departments of Medicine, Epidemiology, and Health Service, University of Washington, Seattle, WA, USA.

^25^ Group Health Research Institute, Group Health Cooperative, Seattle, WA, USA.

^26^ Departments of Epidemiology and Medicine, The Johns Hopkins School of Medicine and Public Health, Baltimore, MD, USA.

^27^ National Heart, Lung, and Blood Institute’s Framingham Heart Study, Framingham, MA, USA.

^28^ Cardiology Division, Department of Medicine, Massachusetts General Hospital, Harvard Medical School, Boston, MA, USA.

^29^ National Heart, Lung, and Blood Institute, Bethesda, MD, USA.

^30^ Department of Physiology and Biophysics, University of Mississippi, Jackson, MS, USA.

^31^ Laboratory of Epidemiology, Demography, and Biometry, Intramural Research Program, National Institute on Aging, National Institutes of Health, Bethesda, MD, USA.

^32^ Netherlands Genomics-Initiative-Sponsored Netherlands Consortium for Healthy Aging, Rotterdam, the Netherlands.

^33^ Department of Epidemiology, Erasmus University Medical Center, Rotterdam, the Netherlands.

^34^ Department of Biostatistics, Boston University School of Public Health, Boston, MA, USA.

^35^ Houston Institute of Molecular Medicine, University of Texas, Houston, TX, USA.

* Corresponding authors.

† Equal Contributos.

**Correspondent author:**

Mary K. Wojczynski, Ph. D., M.P.H.

Department of Genetics, Division of Statistical Genomics, Washington University School of Medicine

Campus Box 8506; 4444 Forest Park Blvd; St. Louis, MO, 63108-2212, USA

Phone: (314) 362.5627; Fax: (314) 362.4227

E-mail: mwojczynski@wustl.edu

Ingrid B. Borecki, Ph. D.

Department of Genetics, Division of Statistical Genomics, Washington University School of Medicine

Campus Box 8506; 4444 Forest Park Blvd; St. Louis, MO, 63108-2212, USA

Phone: (314) 362.3690; Fax: (314) 362.4227

E-mail: iborecki@wustl.edu

Muredach Reilly, M.B., M.S.

Cardiovascular Institute, Perelman School of Medicine at the University of Pennsylvania

11-136 Smilow Center, Building 421, 3400 Civic Center Blvd., Philadelphia, PA, 19104, USA.

Phone: (215) 573-1214

E-mail: muredach@mail.med.upenn.edu

**Subject Codes:** Genetics of Cardiovascular Disease

**Key Words:** atherosclerosis, coronary disease, genetics, meta-analysis

**Table of Contents**

1. PARTICIPATING STUDY DESCRIPTIONS…………………………………………………………………………………… 7
   - - 1. National Heart Lung and Blood Institute (NHLBI) Family Heart Study (FamHS)………………… 7
       2. Candidate Gene Association Resource (CARe) Cohorts …………………………………………………… 8
2. Jackson Heart Study (JHS) and JHS-ARIC……………………………………………………………………. 8
3. Coronary Artery Risk Development In Young Adults (CARDIA)…………………………………… 9
4. Multi-Ethnic Study of Atherosclerosis (MESA)…………………………………………………………… 9
   - - 1. Multi-Ethic Study of Atherosclerosis (MESA) Family/Air…………………………………………………… 10
       2. The Johns Hopkins Genetic Study of Atherosclerosis Risk (GeneSTAR)…………………………….. 11
       3. Genetic Epidemiology Network of Arteriopathy (GENOA)……………………………………………….. 12

B. ASCERTAINMENT AND DESCRIPTION OF FAMILY DATA USED FOR HERITABILITY ESTIMATES…… 13

1. FamHS……………………………………………………………………………………………………………………………… 13

2. JHS (Family Component)………………………………………………………………………………………………….. 13

3. MESA Family/Air (MESA Family subset)…………………………………………………………………………… 14

4. GeneSTAR………………………………………………………………………………………………………………………… 14

5. GENOA…………………………………………………………………………………………………………………………….. 14

1. CAC MEASUREMENT AND ANALYSIS DEFINITION ………………………………………………………………….. 14
   - - 1. FamHS……………………………………………………………………………………………………………………………… 15
       2. Candidate Gene Association Resource (CARe) Cohorts……………………………………………………. 16
          1. JHS and JHS-ARIC……………………………………………………………………………………………………. 16
          2. CARDIA…………………………………………………………………………………………………………………… 16
          3. MESA……………………………………………………………………………………………………………………… 17
       3. MESA Family/Air……………………………………………………………………………………………………………… 17
       4. GeneSTAR………………………………………………………………………………………………………………………… 18
       5. GENOA…………………………………………………………………………………………………………………………….. 18
2. ADDITIONAL CVD RISK FACTOR DEFINITIONS …………………………………………………………………………18

E. COHORT-SPECIFIC GENOTYPING AND QUALITY CONTROL …………………………………………………….. 19

1. FamHS………………………………………………………………………………………………………………………………… 19

2. CARe Cohorts (JHS, JHS-ARIC, CARDIA, and MESA)……………………………………………………………… 20

3. MESA Family/Air………………………………………………………………………………………………………………… 21

4. GeneSTAR………………………………………………………………………………………………………………………….. 21

5. GENOA……………………………………………………………………………………………………………………………….. 22

F. GENOTYPE IMPUTATION………………………………………………………………………………………………………… 22

1. FamHS………………………………………………………………………………………………………………………………… 22

2. CARe Cohorts (JHS, JHS-ARIC, CARDIA, and MESA)……………………………………………………………… 23

3. MESA Family/Air………………………………………………………………………………………………………………… 23

4. GeneSTAR………………………………………………………………………………………………………………………..… 23

5. GENOA…………………………………………………………………………………………………………………………..…… 24

G. PRINICIPAL COMPONENT ANALYSIS (PCA) AND ADJUSTMENT FOR POPULATION STRATIFICATION……………………………………………………………………………........................................... 24

1. FamHS………………………………………………………………………………………………………………………………… 24

2. CARe Cohorts (JHS, JHS-ARIC, CARDIA, and MESA)……………………………………………………………… 25

*CARDIA*

3. MESA Family/Air………………………………………………………………………………………………………………… 25

4. GeneSTAR………………………………………………………………………………………………………………………….. 26

5. GENOA………………………………………………………………………………………………………………………………. 26

H. CHARGE Consortium European Americans CAC Validation Cohort…………………………………………. 27

LIST OF ABBREVIATIONS………………………………………………………………………………………………………………. 28

COMPETING INTERESTS……………………………………………………………………………………………………………….. 30

AUTHOR’S CONTRIBUTIONS…………………………………………………………………………………………………………. 30

ACKNOWLEDGEMENTS……………………………………………………………………………………………………..…………. 30

FUNDING SOURCES…………………………………………………………………………………………………………….………… 30

SUPPLEMENTAL TABLES……………………………………………………………………………………………………..………… 32

Table S1. Cohort-specific genotyping, imputation, and quality control procedures/criteria…… 33

Table S2. AA CAC meta-analysis SNP ‘top hits’ and their assessment in the CHARGe EA CAC meta-analysis………………………………………………………………………………………………………..……………. 35

Table S3. Assessment in African-Americans of SNPs previously associated with CAC in the CHARGe EA CAC Meta-Analysis…………………………………………………………………………………………… 38

Table S4. Assessment in African-Americans of loci previously associated with coronary artery disease……………………………………………………………………………………………………………………… 44

Table S5. SNP signals within EA and AA LD blocks at the 9p21 region as defined by CARe AA CHD GWAS………………………………………………………………………………………………………..……………….. 46

Table S6. Participant characteristics of the CHARGE EA CAC sample………………….…………………… 53

Table S7. Assessment of SNP associations in *PHACTR1* region in the AA CAC Meta-Analysis…. 54

Table S8. Attributes of top *PHACTR1* SNPs from O’Donnell, CardioGRAM, MIGEN, and AA CAC obtained from HapMap………………………………………………………………………………………………. 58

SUPPLEMENTAL FIGURE LEGENDS………………………………………………………………….……………………………..59

Figure S1. Quantile-quantile plots of AA CAC GWAS results from each study……………………….. 60

Figure S2. Linkage disequilibrium plots from HapMap………………………………………………………….. 60

Figure S3. Regional plots of association results for the region from 12.7 Mb – 13.3 Mb in *PHACTR1……………………………………………………………………………………………………………………………..* 60

SUPPLEMENTAL REFERENCES………………………………………………………………………………………………………. 61

**SUPPLEMENTAL METHODS**

1. **PARTICIPATING STUDY DESCRIPTIONS**
   - - 1. **National Heart Lung and Blood Institute (NHLBI) Family Heart Study (FamHS)**

The FamHS is a large, multicenter, population-based, geographically diverse, genetic epidemiologic family study designed to identify genetic and environmental factors, and their interactions, that influence the risk of atherosclerosis and CHD[[1](#_ENREF_1)](https://dsgweb.wustl.edu/PROJECTS/MP1.html). It was begun in 1992 with the ascertainment of 1,200 families, half randomly sampled and half selected based on excess coronary heart disease (CHD) or risk factor abnormalities compared to age- and sex-specific population rates[[1](#_ENREF_1)]. The four population-based parent studies were the Framingham Heart Study, the Utah Family Tree Study, and two ARIC centers (Minneapolis, MN, and Forsyth County, NC). During the clinic visit, consenting participants were examined for a broad range of phenotypes in general domains of CHD, atherosclerosis, cardiac and vascular function, inflammation and hemostasis, lipids and lipoproteins, blood pressure, diabetes and insulin resistance, pulmonary function, diet, habitual activity, medical history, medications, psychosocial factors, anthropometry, and DNA. Although there were no explicit ethnicity exclusions, approximately 92% of the subjects were Caucasian.

Approximately 8 years later, participants belonging to the largest pedigrees were invited back for a second clinical exam (visit 2). An additional field center at University of Alabama - Birmingham was added explicitly to recruit African-American families from the FamHS ancillary study HyperGEN (Hypertension Genetic Epidemiology Network). A total of 633 of the 2,010 HyperGEN African-American subjects were enrolled and examined using a standard clinic protocol for all field centers[[2](#_ENREF_2)]. Thirty four percent of the subjects are male, and the mean age of all subjects is 53 years (SEM 0.43; range 30-83). The primary assessment was a CT scan to assess coronary and aortic artery calcification (CAC and AAC, respectively). Target phenotypes were coronary artery disease (CAD), preclinical atherosclerosis and cardiovascular risk factors, including anthropometrics, lipids, inflammation biomarkers (sICAM1, MCP1, CRP), lifestyle (alcohol, exercise, income, smoking), and medical history[[1](#_ENREF_1), [2](#_ENREF_2)].

- - - 1. **Candidate Gene Association Resource (CARe) Cohorts**
         1. **Jackson Heart Study (JHS) and JHS-ARIC**

The Jackson Heart Study (JHS) is a prospective population-based study to seek the causes of the high prevalence of common complex diseases among African Americans in the Jackson, Mississippi metropolitan area, including cardiovascular disease, type-2 diabetes, obesity, chronic kidney disease, and stroke[[3](#_ENREF_3)]. During the baseline examination period (2000-2004) 5,301 self-identified African Americans were recruited from four sources, including (1) randomly sampled households from a commercial listing; (2) Atherosclerosis Risk in Communities (ARIC) Study participants; (3) a structured volunteer sample that was designed to mirror the eligible population; and (4) a nested family cohort. Unrelated participants were between 35 and 84 years old, and members of the family cohort were ≥ 21 years old when consent for genetic testing was obtained and blood was drawn for DNA extraction. Based on DNA availability, appropriate informed consent, and genotyping results that met quality control procedures, genotype data were available for 3,030 individuals, including 885 who are also ARIC participants. CAC was measured on a subset of JHS participants (n=1,414) during the second JHS clinic examination. Thus in the current study, JHS CAC data comprised the JHS *de novo* recruited sample “JHS” (n=1066) and the JHS sample previously enrolled in ARIC sample “JHS-ARIC” (n=322). The JHS-de novo recruited sample was genotyped as a batch via the CARe study at the Broad Institute. Genotyping of all AA ARIC participants also was performed as a separate batch via the CARe study at the Broad Institute. The recommendation from the CARe study analysis committee was to analyze the” JHS” and “JHS-ARIC” individuals separately because QC of JHS and ARIC genotype data was not 100% identical.

- 1. **Coronary Artery Risk Development In Young Adults (CARDIA)**

The CARDIA study is a prospective, multi-center investigation of the natural history and etiology of cardiovascular disease in African Americans and whites 18-30 years of age at the time of initial examination[[4](#_ENREF_4)]. The CARDIA sample was recruited at random during 1985-86 primarily from geographically based populations in Birmingham AL, Chicago IL, and Minneapolis, MN, and in Oakland, CA, from the membership of the Kaiser-Permanente Health Plan. The initial examination included 5,115 participants selectively recruited to represent proportionate racial, gender, age, and education groups from each of the four communities. Each participant’s age, race, and sex were self-reported during the recruitment phase and verified during the baseline clinic visit. Details of the study design and procedures for data collection have been published. From the time of initiation of the study in 1985-1986 (baseline examination), six follow-up examinations have been conducted at years 2, 5, 7, 10, 15, and 20. DNA extraction for genetic studies was performed at the Y10 examination. After taking into account availability of adequate amounts of high quality DNA, appropriate informed consent and genotyping quality control and assurance procedures, genotype data were available on 671 African-American individuals.

- 1. **Multi-Ethnic Study of Atherosclerosis (MESA)**

The Multi-Ethnic Study of Atherosclerosis (MESA) is a National Heart, Lung and Blood Institute-sponsored, population-based investigation of subclinical cardiovascular disease and its progression. A full description of the design and recruitment process has been reported previously[[5](#_ENREF_5)]. In brief, a total of 6,814 individuals, aged 45 to 84 years, were recruited from six US communities (Baltimore City and County, MD; Chicago, IL; Forsyth County, NC; Los Angeles County, CA; New York, NY; and St. Paul, MN) between July 2000 and August 2002. Participants were excluded if they had physician-diagnosed cardiovascular disease prior to enrollment, including angina, myocardial infarction, heart failure, stroke or transient ischemic attack (TIA), resuscitated cardiac arrest or a cardiovascular intervention (e.g., CABG, angioplasty, valve replacement, or pacemaker/defibrillator placement). Pre-specified recruitment plans identified four racial/ethnic groups (White European-American, African-American, Hispanic-American, and Chinese-American) for enrollment, with targeted oversampling of minority groups to enhance statistical power. The comprehensive baseline MESA examination included a clinic visit, serum analyses, and computed tomography (CT) examination of the chest and heart. Information regarding the participants’ demographic data and medical history, including medication use, was obtained by questionnaire. Ethnicity was self-reported. The institutional review boards at each participating institution approved MESA and each individual participant provided informed written consent prior to enrollment.

1. **Multi-Ethic Study of Atherosclerosis (MESA) Family/Air**

The MESA Family/Air effort for this study included two ancillary studies to the Multi-Ethnic Study of Atherosclerosis (MESA). Most participants came from the MESA Family study. The goal of MESA Family is to apply modern genetic analysis and genotyping methodologies to delineate the genetic determinants of early atherosclerosis, utilizing the established organizational structures of MESA and Genetic Centers at Cedars Sinai Medical Center and the University of Virginia. In order to identify and locate genetic variation contributing to the genetic risk for cardiovascular disease (CVD), MESA Family looks at the early changes of atherosclerosis within families (mainly siblings). The study recruited 2128 individuals from 594 families, yielding 3,026 sibpairs divided between African Americans and Hispanic-Americans. MESA Family studied siblings of index subjects from the MESA study as well as from new sib-pair families (with the same demographic characteristics). The MESA Family cohort was recruited from the six MESA Field Centers. MESA Family participants underwent the same examination as MESA participants, with CT scans during April 2004 – January 2007. Of the 2128 MESA Family participants, 1681 (940 African-Americans) were not previously enrolled in the MESA parent study.

The MESA Family/Air effort also included participants from another MESA ancillary study, MESA Air. The goal of MESA Air is to prospectively examine the relationships between long-term ambient air pollution exposures and the progression of subclinical cardiovascular disease in a multi-city, multi-ethnic cohort. Like MESA Family, MESA Air is built on the foundation of the ongoing MESA study. The cohort for the MESA Air study currently includes 6226 subjects. However, most of these participants were enrolled in the parent MESA study (5479) or MESA Family (490). Only 257 were specifically enrolled for MESA Air, and only a fraction of these were African-American. MESA Air participants underwent the same examination as MESA participants, during February 2006 – May 2007.

Only newly recruited members of MESA Family and MESA Air were analyzed by MESA Family/Air for this effort because subjects in the MESA cohort (CARe MESA) were analyzed by the Candidate Gene Association Resource (CARe) project. Genotyping for MESA Family and MESA Air participants was performed on the Affymetrix Genome-Wide Human SNP Array 6.0.

1. **The Johns Hopkins Genetic Study of Atherosclerosis Risk (GeneSTAR)**

GeneSTAR is a longitudinal family based epidemiologic study examining inflammatory, platelet, lipoprotein, blood pressure, metabolic, and vascular property determinants of incident atherosclerotic coronary heart disease and attendant co-morbidity (stroke and peripheral vascular disease) among apparently healthy first degree adult relatives of hospitalized probands with documented premature coronary disease events prior to 60 years of age.  Probands were hospitalized at one of 10 Baltimore area hospitals. Between 1990 and 2006, GeneSTAR enrolled 1342 asymptomatic, apparently healthy young African American siblings (<60 years of age) and offspring (21-59 years of age) of both the proband and his/her siblings, as well as co-parents of the offspring; of these, 272 had both genotype data which passed strict quality control measures and coronary artery calcium (CAC) phenotype data. African American participants represented 140 different families. Additionally 1884 whites were enrolled in the same period. The current study is limited to African Americans.

1. **Genetic Epidemiology Network of Arteriopathy (GENOA)**

GENOA is one of four networks in the NHLBI Family-Blood Pressure Program (FBPP)[[6](#_ENREF_6)]. GENOA's long-term objective is to elucidate the genetics of target organ complications of hypertension, including both atherosclerotic and arteriosclerotic complications involving the heart, brain, kidneys, and peripheral arteries. The longitudinal GENOA Study recruited European-American and African-American sibships with at least 2 individuals with clinically diagnosed essential hypertension before age 60 years. All other members of the sibship were invited to participate regardless of their hypertension status. The current study is limited to self-identified African Americans recruited at the Jackson, Mississippi field center. Between 2009 and 2011, 657 GENOA participants received computed tomography (CT) scans for CAC. Because probands for GENOA were recruited through the Atherosclerosis Risk in Communities (ARIC) Jackson field center participants, we excluded ARIC participants from analyses. Our study group included a total of 316 participants ages 56-86 years.

**B. ASCERTAINMENT AND DESCRIPTION OF FAMILY DATA USED FOR HERITABILITY ESTIMATES**

**1. FamHS**

Nuclear African-American families were recruited from the field center at University of Alabama - Birmingham during the second Family Heart Study visit. These families were participants of the FamHS ancillary study HyperGEN (Hypertension Genetic Epidemiology Network). HyperGEN participants were recruited from multiply-affected hypertensive sibships ascertained from population-based cohorts or the community. The study later included siblings and offspring of the original sibpair. Probands were identified by the onset of hypertension before age 60 and the presence of at least one additional hypertensive sibling who was willing to participate[[7](#_ENREF_7)]. A total of 633 of the 2,010 HyperGEN African-American subjects were enrolled and examined using a standard clinic protocol for all field centers[[2](#_ENREF_2)].

**2. JHS (Family Component)**

A nested Family Study was recruited from among the relatives of participants in the overall JHS cohort. Participants having at least two full siblings and four other first-degree relatives ≥ 21 years old and living in one of the three counties (Hind, Madison, Rankin) surrounding Jackson, MS, were eligible to be index participants for the Family Study. Names, contact information, family structure data, and permission to contact relatives were collected from these index participants, and these data were extended in the field as additional family members were contacted and recruited. In addition, because the overall JHS recruitment strategy was household-based, participants in the overall cohort who shared an address were queried regarding their relatedness, and these relationships were recorded. The final JHS Family Study includes 1,486 members of 264 families, nested within an overall cohort of 5,301 participants.

**3. MESA Family/Air (MESA Family subset)**

Using the existing framework of MESA, MESA Family enrolled 2128 individuals from 594 nuclear families from six MESA Field Centers. Some MESA Family participants are siblings of index subjects from MESA; other participants are new sib-pair families with the same demographic characteristics. MESA Family participants underwent the same examination as MESA participants, between May, 2004 and May, 2007.

**4. GeneSTAR**

Siblings were identified from a proband with documented coronary artery disease prior to age 60, enrolled from any of 10 Baltimore area hospitals between 1983 and 2006. This study included only African Americans with genotyping and CAC measurement, which resulted in 150 sibling pairs and 90 half sibling pairs.

**5. GENOA**

GENOA included African-American sibships with at least 2 individuals with clinically diagnosed essential hypertension before age 60 years. All other members of the sibship were invited to participate regardless of their hypertension status. Between 2009 and 2011, 657 GENOA-Jackson participants received CT scans for CAC. Because this was the fourth examination for this cohort, some participants no longer had siblings in the study. Heritability was estimated using 503 participants (196 singletons, 87 sibships of size 2, 25 sibships of size 3, 7 sibships of size 4 and 6 sibships of size 5) ages 50 to 75 at time of the CT scan.

**C. CAC MEASUREMENT AND ANALYSIS DEFINITION**

All studies assessed CAC using standard computed tomography (CT, performed either by electron beam or multi-detector CT) imaging methods. Scans were interpreted at the corresponding sites of the independent studies but all investigators applied standardized methods using published software and reading algorithms[[8-10](#_ENREF_8)]. Calcified plaque was quantified by the Agatston method [[11](#_ENREF_11)] and the total calcium score, summing over the individual coronary arteries (i.e. left main, left anterior descending, circumflex, and right coronary arteries), was used in these analyses. Each study performed quality control in obtaining CAC measurements. In order to maintain comparability to published results for samples of European descent[[12](#_ENREF_12)], we used the identical phenotypic measure, ln(CAC+1) for all participants (even when CAC=0), in our primary analysis. In secondary analyses, we assessed CAC dichotomously (present/absent), ln(CAC) for those with CAC >0, ; and ln(CAC+1) in older participants (men≥50 and women≥60). These secondary analyses produced similar results to the primary analysis and therefore these data are not presented.

**Cohort-specific CAC Measurement**

- - - 1. **FamHS**

Participants underwent a cardiac multi-detector CT exam using a standardized protocol as described previously[[8](#_ENREF_8)]. CT images from all field centers were sent to the central reading center at Wake Forest University to compute CAC scores. Images were analyzed on a dedicated image processing workstations (GE Healthcare Advantage Windows, SmartScore Application, Waukesha, WI) providing an Agatston score modified to account for slice thickness was calculated. The sum of the individual coronary arteries (i.e. left main, left anterior descending, circumflex, and right coronary arteries) was reported as the total calcium score, averaged over the first and second CT scan series. More details about CAC measurement and reading can be found elsewhere[[13](#_ENREF_13), [14](#_ENREF_14)].

- - - 1. **Candidate Gene Association Resource (CARe) Cohorts**
         1. **JHS and JHS-ARIC**

CT was performed in JHS Exam 2 by a protocol that included the heart and lower abdomen, using a Lightspeed 16 Pro, 16-channel multidetector system equipped with cardiac gating (GE Healthcare, Milwaukee, WI). Quality control and image analysis were performed at the JHS core reading center at Wake Forest University School of Medicine. CT scans of the coronary arteries were based on standard protocols developed for the MESA and CARDIA studies[[15](#_ENREF_15)]. CAC was measured in CT images by trained and experienced technologists. The Agatston score, modified to account for slice thickness, was used to quantify calcified artery plaque, computed by multiplying the area of each lesion by a weighted attenuation score (in Hounsfield units) on a TeraRecon Aquarius Workstation. The sum of the individual coronary arteries (i.e. left main, left anterior descending, circumflex, and right coronary arteries) was reported as the total calcium score. Reproducibility of CAC was 0.99.

- - - - 1. **CARDIA**

CAC was measured by computed tomography (CT) of the chest at the Y20 examination. Electron beam CT (Chicago and Oakland field centers) and multidetector CT (Birmingham and Minneapolis filed centers) scanners were used to obtain two sequential scans from the root of the aorta to the apex of the heart. Detailed methods of CT scanning and calcium measurement in CARDIA have been previously described[[8](#_ENREF_8)]. A calcium score was generated for every calcified lesion with an artery and summed up for all lesions in the artery. Scores for all arteries (i.e. left main, left anterior descending, circumflex, and right coronary arteries) were summed up to generate a total calcium score for each participant[[11](#_ENREF_11)]. Presence of CAC was defined as a total calcium score >0. There was a high rate of agreement (96.5%) between sequential scans for presence of CAC. For concordant scans, the mean of the two scores was used as the calcium score. Discordant scans were re-examined and categorized as: definitely negative, probably negative, cannot tell, probably positive, and definitely positive. The calcium score was set to 0 for the first three categories and the score of the positive scan for the last two categories. The CAC score was set to missing for participants who had stents or coronary artery bypass grafting (CABG) surgery.

- - - - 1. **MESA**

All MESA participants underwent baseline CT scans. Three institutions used an electron beam computed tomography (EBCT) Imatron C150 scanner (GE Medical Systems, Milwaukee, WI), while three institutions used 4-slice multidetector CT (MDCT) scanners. Full details concerning the equipment, scanning methods, and CT quality control in MESA, including image calibration, phantom adjustment and inter-scanner reproducibility, have been reported previously[[9](#_ENREF_9), [16](#_ENREF_16)]. All scans were sent to a central MESA CT reading center (Harbor-UCLA Research and Education Institute, Los Angeles, CA) where they were analyzed by a single reader using proprietary offline software that utilizes the Agatston methodology. Total calcium score was the sum of the following main arteries: left main, left anterior descending, circumflex, and right coronary arteries.

- - - 1. **MESA Family/Air**

All MESA participants underwent baseline CT scans. Three institutions used an electron beam computed tomography (EBCT) Imatron C150 scanner (GE Medical Systems, Milwaukee, WI), while three institutions used 4-slice multidetector CT (MDCT) scanners. Full details concerning the equipment, scanning methods, and CT quality control in MESA, including image calibration, phantom adjustment and inter-scanner reproducibility, have been reported previously[[9](#_ENREF_9), [16](#_ENREF_16)]. All scans were sent to a central MESA CT reading center (Harbor-UCLA Research and Education Institute, Los Angeles, CA) where they were analyzed by a single reader using proprietary offline software that utilizes the Agatston methodology[[11](#_ENREF_11)]. Total calcium score was the sum of the main arteries (i.e. left main, left anterior descending, circumflex, and right coronary arteries).

- - - 1. **GeneSTAR**

A Siemens Volume Zoom Multirow Detector Computed Tomography (CT) Scanner was used to obtain 30 to 40 adjacent 3 mm-thick axial slices. Coronary calcium was quantified using the standard Agatston scoring system on a 3D Virtuoso workstation (Siemens Medical Solutions, Iselin, NJ) using standard coronary artery scoring software [[11](#_ENREF_11)]. Total calcium score was the sum of the main arteries (i.e. left main, left anterior descending, circumflex, and right coronary arteries).

- - - 1. **GENOA**

CAC was measured with the scanning procedure for cardiac gated CT scans of the coronary arteries based on the standard protocols developed as part of the NHLBI’s MESA and CARDIA studies[[8](#_ENREF_8)]. Participants were scanned on a GE LightSpeed Pro 16 multidetector scanner. All CT scan sequences were performed with suspended respiration and a single breath hold. CT images were transmitted to the reading center at Wake Forest University to compute CAC scores. A modified CAC Agatston score for loci having CT numbers > 130 Hounsfield units and a minimum lesion size of 1.0 mm^2^ was used. The CAC score was calculated as the sum of the CAC scores in the four main coronary arteries (i.e. left main, left anterior descending, circumflex, and right coronary arteries).

1. **ADDITIONAL CVD RISK FACTOR DEFINITIONS**

All studies gathered participant information on CVD risk factors. While not used in the analysis of the data, smoking status, hypertension, diabetes, and statin use are of interest to describe our study population. All studies report smoking status as current versus former/never smokers. Hypertension was defined by each study. Most studies defined hypertension as a blood pressure ≥140/90 mm Hg or currently taking antihypertensive medications or self-reported hypertension; however GENOA only had information on self-reported hypertension. Diabetes is defined using the standard definition of fasting glucose ≥126 mg/dl or currently taking medication for diabetes. Statin use is calculated based on what current medications participants were taking. Current medications were then coded into medication classes, one of which is statins, to determine the percentage of participants from each study currently using statins.

**E. COHORT-SPECIFIC GENOTYPING AND QUALITY CONTROL**

**1. FamHS**

FamHS used the Illumina Human 1M-DuoV3 array for all subjects. Genotypes were called using Genome Studio software (BeadStudio algorithm). Quality control (QC) was performed before imputation. To assess Mendelian errors, we ran LOKI[[17](#_ENREF_17)] on our family data and removed 15,948 single nucleotide polymorphisms (SNPs) with call rate < 0.99 or with enough Mendelian errors to be considered outlier SNPs. We also removed 1 individual that had an unacceptable number of Mendelian errors (n=1,446), thus making this individual an outlier compared to the rest of the population. As a final familial QC check, we used GRR software[[18](#_ENREF_18)] to check familial relationships based on identity by state (IBS); corrections to the family relationships were made as warranted by the data, including the exclusion of one individual. Quality control procedures for SNPs included eliminating: SNPs with minor allele frequency (MAF) <1% (n=85,370), SNPs with deviations from Hardy-Weinberg equilibrium (HWE, p<1E-06, n=783), and SNPs that were not in HapMap (n=264,407). This allowed for 745,148 genotyped SNPs passing quality control and used for the imputation.

**2. CARe Cohorts (JHS, JHS-ARIC, CARDIA, and MESA)**

JHS, JHS-ARIC, CARDIA, and MESA samples were genotyped at the Broad Institute using the Affymetrix Genome-Wide Human SNP Array 6.0 (Affy6.0) according to the manufacturer’s recommendations, as part of the NHLBI Candidate Gene Association Resource (CARe) project. Genotyping and quality control procedures have been described in detail[[19](#_ENREF_19)]. The Affymetrix 6.0 genotyping platform interrogates simultaneously 1.8 million markers for genetic variation (906,600 SNPs and 946,000 copy number variation probes). Several quality control (QC) procedures were performed on the genotype data, separately for each cohort. Quantity of double stranded DNA was assessed using PicoGreen® (Molecular Probes, Oregon, USA). To confirm sample identity, genotype concordance was evaluated for 24 SNPs genotyped in the same DNA samples using both Sequenom iPLEX and Affymetrix 6.0. Genome-wide genotype data were used to estimate identity-by-descent (IBD) between all pairwise combinations of samples in order to identify sample duplicates, contaminated samples, and cryptic relationships. We also used IBS/IBD measures to confirm known pedigree data for JHS. SNPs and samples with an unusually high number of Mendel errors were excluded. Heterozygosity rates (in the form of inbreeding coefficients) were estimated to identify problematic DNA samples (poor DNA quality or contaminations). DNA samples with a genome-wide genotyping success rate <95%, duplicate discordance or sex mismatch, SNPs with genotyping success rate <95%, monomorphic SNPs, SNPs with minor allele frequency (MAF) <1%, and SNPs that map to several genomic locations were removed from the analyses. The Hardy-Weinberg equilibrium (HWE) test was performed for all SNPs, but SNPs were not excluded based uniquely on this criterion given the admixed nature of the cohorts genotyped. After applying all quality control filters, the following numbers of African-American participants were available for analysis: JHS=1,066, JHS-ARIC=322, CARDIA=671, and MESA=1,646. In the current study, JHS CAC data comprised of the JHS *de novo* recruited sample “JHS” (n=1066) and the JHS sample previously enrolled in ARIC study “JHS-ARIC” (n=322). The JHS-de novo recruited sample was genotyped as a batch via the CARe study at the Broad Institute. Genotyping of all AA ARIC participants also was performed as a separate batch via the CARe study at the Broad Institute. The recommendation from the CARe study analysis committee was to analyze the” JHS” and “JHS-ARIC” individuals separately because QC of JHS and ARIC genotype data was not 100% identical.

**3. MESA Family/Air**

Genotyping for MESA Family and MESA Air participants was performed on the Affymetrix Genome-Wide Human SNP Array 6.0, and completed with the CARe MESA cohort by the NHLBI CARe project at the Broad Institute (see prior section on CARe Cohorts).

**4. GeneSTAR**

In GeneSTAR, SNP genotyping was performed at deCODE Genetics, Inc. using the Human 1Mv1_C array from Illumina, Inc. where 1,044,094 markers were released with an average call rate per sample of 99.65% and an overall missing data rate of 0.35%.  PLINK v1.06[[20](#_ENREF_20)] was used to detect and remove Mendelian errors. Hardy-Weinberg equilibrium (HWE) and minor allele frequency (MAF) for each SNP was tested in a defined set of independent subjects (n=326) representing the founders of the pedigrees. SNPs missing chromosomal location, monomorphic SNPs, SNPs with HWE p<1E-08, and SNPs with call rate < 90% were excluded from analysis. Participants (n=13) were excluded due to: 1) gender discrepancies, 2) ancestry outliers from any of the first 10 principal components from EIGENSTRAT, or 3) Mendelian inconsistency rate > 5%.

**5. GENOA**

A total of 1,263 African American participants from GENOA were genotyped on the Affymetrix Genome-Wide Human SNP Array 6.0 using the protocol outlined by Affymetrix (Affymetrix, 2007) at the Mayo Clinic in Rochester, Minnesota. Samples and SNPs with a call rate <95% were removed. Samples demonstrating sex mismatch, duplicate samples, and samples with low identity-by-state with all other samples were also removed. The SNPs used for imputation included 550,325 SNPs genotyped on the Affymetrix 6.0 platform.

**F. GENOTYPE IMPUTATION**

**1. FamHS**

To create an imputation model, we used phased haplotypes from CEU+YRI from HapMap Phase 2 (release 22, build 36) as a reference. We created a subset of 200 unrelated subjects, with highest average genome-wide genotyping call rates and evenly distributed between sexes, to create a framework imputation map based on the Illumina Human 1M-DuoV3 array using Markov Chain Haplotyper (MaCH) 1.0.16[[21](#_ENREF_21)]. SNPs used for the imputation model had a call rate >0.99, MAF >0.01, and no deviation from HWE (p>1E-06), leaving a total of 745,148 SNPs for the imputation. The parameters estimated from this imputation were applied to the remaining subjects to estimate imputed genotype dosages. This process resulted in imputed SNP dosages for 2,199,259 SNPs among subjects of African descent. To create the hybrid dataset, the original genotypes were converted to dosages. Genotyped SNPs were excluded from the hybrid dataset if a genotyped SNP was not in the mlinfo file. If the genotyped SNPs alleles were inconsistent with the corresponding mlinfo alleles (indicative or a potential genotyping error) the genotyped SNP was excluded and its imputed values were used in the hybrid dataset. Imputed SNP dosages were used in the hybrid dataset for any genotyped SNP with a call rate <0.99, MAF <0.01 (or >0.99), and HWE p-value <1E-06. For the remaining genotyped SNPs, genotyped dosage data was then merged over imputed data in the mldose file whenever the measured genotype was available, with missing genotyped data kept as missing in the hybrid dataset. This process led to a hybrid dataset with 2,199,259 SNPs.

**2. CARe Cohorts (JHS, JHS-ARIC, CARDIA, and MESA)**

Imputation in African-Americans was performed using MaCH 1.0.16, which requires phased reference haplotypes[[21](#_ENREF_21)]. Individuals with pedigree relatedness or cryptic relatedness (pi_hat > 0.05) were filtered prior to imputation. SNPs with MAF ≥1%, call rate ≥95% and HWE *P* ≥1E-06 were used for imputation. A combined CEU+YRI reference panel from HapMap phase 2 (release 22, build 36) was used[[22](#_ENREF_22)]. A randomly selected subset of individuals from each cohort sample was used to generate recombination and error rate estimates. These rates were then used to estimate genotype dosages in all sampled individuals across the entire reference panel for over 2 million SNPs.

**3. MESA Family/Air**

Prior to imputation, 11,643 of the 909,622 genotyped SNPs on the Affymetrix 6.0 array, were dropped because they were monomorphic, had observed heterozygosity > 53%, or had a missing rate > 5%, across all samples. These dropped SNPs were imputed if they were reference panel SNPs. Duplicate samples, samples involved in unresolved gender mismatches, cryptic duplicates, and having call rate < 95% were dropped prior to imputation. Genotypes were imputed in African Americans separately from other MESA ethnic group using the program IMPUTE2. For African-Americans the HapMap I + II CEU+YRI+CHB+JPT (rel#22, NCBI Build 36, dbSNP b126) was used as the reference population.

**4. GeneSTAR**

GeneSTAR used MaCH (version 1.0.16)[[21](#_ENREF_21)] to impute all autosomal SNPs on HapMap (2,199,259 SNPs), using combined CEU+YRI haplotypes (HapMap II, release 22, build 36) as a reference panel. From a total of 1,005,342 genotyped SNPs, we used 687,132 SNPs in the imputation after filtering out 1289 SNPs with HWE p<1E-06, 65,760 SNPs with MAF < 1%, and 316,955 SNPs that were not present on HapMap. We selected 200 African American individuals (pre-screened to be unrelated; 104 males and 96 females) by prioritizing those individuals with low missing genotyping. None of these 200 individuals were identified as outliers by EIGENSTRAT[[23](#_ENREF_23)] (default parameters). We used the 200 pre-selected individuals to infer model parameters first, and subsequently applied the model on all African American GeneSTAR individuals. MaCH output imputed dosages for all 2,199,259 SNPs on the combined CEU+YRI panel. The final imputations were created by keeping only the best quality of imputed data. Where available, genotyped SNP data was used in all analyses; for SNPs that were not genotyped, imputed dosage data was used in all analyses.

**5. GENOA**

Imputation in African-Americans was performed using the single-step approach implemented in MaCH 1.0.16, which requires phased reference haplotypes[[21](https://mail.google.com/mail/u/0/?shva=1#13ddc0b46da33e71__ENREF_21)]. Samples demonstrating sex mismatch, duplicate samples, and samples with low identity-by-state with all other samples were removed prior to imputation. SNPs with MAF ≥1% and call rate ≥95% (550,325 SNPs genotyped on the Affymetrix 6.0 platform) were used for imputation.  A combined CEU+YRI reference panel from HapMap phase 2 (release 22, build 36) was used[[22](https://mail.google.com/mail/u/0/?shva=1#13ddc0b46da33e71__ENREF_22)].  We estimated genotype dosages in all sampled individuals across the entire reference panel for over 2 million SNPs.

**G. PRINICIPAL COMPONENT ANALYSIS (PCA) AND ADJUSTMENT FOR POPULATION STRATIFICATION**

**1. FamHS**

Cryptic stratification was accounted for by estimating the first 10 principal components (PCs) using EIGENSTRAT[[23](#_ENREF_23)] in genotype data of all available independent subjects (N=231). The principal component model was then applied to the remaining (non-independent) family members. All 10 principal components were included as covariates in the analysis for CAC.

**2. CARe Cohorts (JHS, JHS-ARIC, CARDIA, and MESA)**

We used PCA as implemented in EIGENSTRAT[[23](#_ENREF_23)] on the cleaned CARe African-American Affy6.0 genotype data. PCA was also used as a screening tool to detect extreme sample outliers before quality control checks, and no significant sample outliers were observed at this step for all studies but CARDIA (see below). The first 10 principal components, derived in EIGENSTRAT[[23](#_ENREF_23)], were used as covariates in the CAC regression analysis.

***CARDIA***

PCs estimation was performed as part of the CARe study and follows the same methodologies. PCA was used as a screening tool to detect extreme sample outliers before quality control checks. For all CARe cohorts except CARDIA, we did not observe significant sample outliers at this step. For CARDIA, however, the second principal component separated 210 samples from the rest of the samples (the first PC still captured global ancestry). These 210 samples were characterized by low genotyping success rate (<98%), low heterozygosity (inbreeding coefficient F <-0.15), and belonged to four different chemistry plates (the CARDIA DNA collection was genotyped on 16 plates). These 210 CARDIA samples were determined to have poor genotyping characteristics and removed from subsequent QC analyses.

The first 10 principal components were used as covariates in the analyses.

**3. MESA Family/Air**

For the computation of principal components, 6,849 SNPs in genomic regions that have been shown to harbor long range linkage disequilibrium (LD) were removed. These regions have been shown to influence the choice of principal components (PCs)[[23](#_ENREF_23), [24](#_ENREF_24)]. The PCs were computed on 2,590 self-reported African-Americans separately from all other MESA ethnic groups. Chromosome specific principal components were initially computed to reduce the computational burden; these PCs were later combined to provide the final set of eigenvalues, eigenvectors and projected data. Regression models for the association analysis adjusted for the first three PCs.

**4. GeneSTAR**

EIGENSTRAT[[23](#_ENREF_23)] was run using genotype data on 326 independent subjects, and applied to the remaining family members. The first 10 principal components (PCs) generated from EIGENSTRAT were used as covariates in all CAC regression analyses.

**5. GENOA**

Since GENOA is composed of sibships, we calculated PCs to control for population stratification in an unrelated sample of participants. First, we removed SNPS that had poor imputation quality as measured by the estimated r^2^ between imputed and true genotypes (r^2^<0.8) from MaCH output. Next, we obtained the maximum number of unrelated individuals in our total sample by selecting one sibling randomly from each sibship. In this sub-sample, we calculated the first ten PCs and then used the loading matrix for these PCs to calculate the PC values in the full sample. Next, outliers of more than 6 standard deviations on any of the ten PCs were removed to ensure that the PCs were not capturing variation due to poor quality genotyping or single individuals with a dramatically different admixture profile than the remainder of the sample. A total of 35 participants were removed from the full sample. Next, we again selected an unrelated sub-sample of participants by randomly selecting one participant from each sibship and recalculated the first ten PCs in this sample. Finally, we used the loading matrix to calculate the first ten PCs in the final sample.

**H. CHARGE Consortium European Americans CAC Validation Cohort**

The European-American genome wide association study (GWAS) validation sample comprised 9,992 subjects from 5 CHARGE cohorts (the Age, Gene/Environment Susceptibility-Reykjavik Study (AGES-Reykjavik), the Framingham Heart Study (FHS), the Rotterdam Study-I (RS-I), Rotterdam Study-II (RS-II), and the Genetic Epidemiology Network of Arteriopathy Study (GENOA)). Details of the CHARGE consortium including subject details and study designs, are described elsewhere[[25](#_ENREF_25)] and are reported in an accompanying manuscript[[12](#_ENREF_12)]. For the current analysis, CAC was derived from data provided by CT scan commonly employed in clinical and epidemiological studies. Each study excluded all participants with any CAC measure outside of +/- 2 standard deviations from the mean value. The CHARGE cohort participant characteristics for the current CAC replication analysis are summarized in **Supplemental Table S6**.

**LIST OF ABBREVIATIONS**

NHLBI National Heart, Lung, and Blood Institute

FamHS Family Heart Study

CHD coronary heart disease

HyperGEN Hypertension Genetic Epidemiology Network Study

SEM Standard error of mean

CAC coronary artery calcification

AAC aortic artery calcification

CAD Coronary artery disease

sICAM1 soluble intercellular adhesion molecule 1

MCP1 monocyte chemoattractant protein 1

CRP C-reactive protein

CARe Candidate gene Association Resource

JHS Jackson Heart Study

ARIC Atherosclerosis Risk in Communities Study

JHS-ARIC Jackson Heart Study-Atherosclerosis Risk in Communities Study

CARDIA Coronary Artery Risk Development in Young Adults

MESA Multi-Ethnic Study of Atherosclerosis

TIA transient ischemic attach

CT Computed tomography

CVD cardiovascular disease

GeneSTAR Genetic Study of Atherosclerosis Risk

GENOA Genetic Epidemiology Network of Arteriopathy

FBPP Family Blood Pressure Program

CABG coronary artery bypass grafting

EBCT Electron beam computed tomography

MDCT Multidetector computed tomography

QC quality control

SNP single nucleotide polymorphism

IBS Identity by state

MAF minor allele frequency

HWE Hardy-Weinberg equilibrium

IBD Identity by descent

CEU+YRI European and Yoruban combined HapMap reference population

MaCH Markov Chain Haplotyper

CEU U.S. residents with northern and western European ancestry used in HapMap reference

YRI Yoruban population sample from Nigeria used in HapMap reference sample

CEU+YRI+CHB+JPT European, Yoruban, Chinese, and Japanese combined HapMap reference populatoin

PCA Principal components analysis

PC principal component

LD linkage disequilibrium

GWAS genome-wide association study

CHARGE Cohorts for Heart and Aging Research in Genomic Epidemiology

AGES-Reykjavik Age, Gene/Environment Susceptibility-Reykjavik Study

FHS Framingham Heart Study

RS-I Rotterdam Study-I

RS-II Rotterdam Study-II

GEE Generalized estimating equations

LME linear mixed-effects

AA African Americans

EA European Ancestry

QQ quantile-quantile plot

SE standard error

P p-value

n sample size

Chrom Chromosome

**COMPETING INTERESTS**

The authors do not have any conflicts of interest, financial or otherwise.

**AUTHOR’S CONTRIBUTIONS**

NDW, LAL, THM, XG, SKG, SRH, MEG, DHO, MB, JJC, HAT, DAB, SD, DNP, JFP, BMP, DMB, MAP, WSP, CJO, JGW, TBH, MK, LAC, JIR, MF, LCB, PAP, IBB, and MPR conceived and designed the study. MKW, ML, LFB, KFK, APR, LRY, LQ, LAL, JFF, JH, TY, JAS, BGK, XG, QW, S-JH, BMP, DMB, MAP, WSP, CJO, JGW, TBH, LAC, MF, JIR, LCB, PAP, IBB, and MPR acquired the data. MKW, ML, LFB, KFK, APR, LRY, LQ, CCW, LAL, JFF, JH, TY, JAS, BGK, XG, QW, MB, JJC, S-JH, BMP, DMB, MAP, WSP, CJO, JGW, TBH, LAC, JIR, MF, LCB, and PAP analyzed the data. MKW, ML, LFB, KFK, APR, NDW, LRY, LQ, LAL, JFF, JH, THM, JAS, BGK, XG, QW, SKG, SRH, MEG, DHO, MB, JJC, HAT, DAB, SD, S-JH, DNP, JFP, BMP, DMB, MAP, WSP, CJO, JGW, TBH, MK, LAC, JIR, MF, LCB, PAP, IBB, and MPR interpreted the data. MKW, IBB, and MPR drafted the manuscript. All authors revised the manuscript for important intellectual content, read, and approved the final manuscript.

**ACKNOWLEDGEMENTS**

The authors acknowledge the essential role of all the participating cohorts: NHLBI Family Heart Study, all cohorts (JHS, JHS-ARIC, CARDIA, and MESA) involved in the NHLBI Candidate gene Association Resource (CARe) Consortium studies, MESA Family/Air, GeneSTAR, and GENOA. The collaboration of the CHARGE EA CAC consortia also played a vital role.

**FUNDING SOURCES**

The **National Heart, Lung, and Blood Institute's Family Heart Study (FamHS)** was supported by NIH grants R01-HL-087700 and R01-HL-088215 (Michael A. Province, PI) from NHLBI; and R01-DK-8925601 and R01-DK-075681 (Ingrid B. Borecki, PI) from NIDDK.

The authors from the **CARe** Consortium wish to acknowledge the support of the National Heart, Lung, and Blood Institute and the contributions of the research institutions, study investigators, field staff and study participants in creating this resource for biomedical research. The following studies have contributed parent study data, ancillary study data, and DNA samples through the Broad Institute (N01-HC-65226):

**Coronary Artery Risk in Young Adults (CARDIA)**: University of Alabama at Birmingham (N01-HC-48047), University of Minnesota (N01-HC-48048), Northwestern University (N01-HC-48049), Kaiser Foundation Research Institute (N01-HC-48050), University of Alabama at Birmingham (N01-HC-95095), Tufts-New England Medical Center (N01-HC-45204), Wake Forest University (N01-HC-45205), Harbor-UCLA Research and Education Institute (N01-HC-05187), University of California, Irvine (N01-HC-45134, N01-HC-95100); **Jackson Heart Study (JHS)**: Jackson State University (N01-HC-95170), University of Mississippi (N01-HC-95171), Tougaloo College (N01-HC-95172); **Multi-Ethnic Study of Atherosclerosis (MESA):** University of Washington (N01-HC-95159),Regents of the University of California (N01-HC-95160), Columbia University (N01-HC-95161), Johns Hopkins University (N01-HC-95162, N01-HC-95168), University of Minnesota (N01-HC-95163), Northwestern University (N01-HC-95164), Wake Forest University (N01-HC-95165), University of Vermont (N01-HC-95166), New England Medical Center (N01-HC-95167), Harbor-UCLA Research and Education Institute (N01-HC-95169), Cedars-Sinai Medical Center (R01-HL-071205), University of Virginia (subcontract to R01-HL-071205).

The **Coronary Artery Risk Development in Young Adults (CARDIA)** study is funded by contracts N01-HC-95095, N01-HC-48047, N01-HC-48048, N01-HC-48049, N01-HC-48050, N01-HC-45134, N01-HC-05187, N01-HC-45205, and N01-HC-45204 from the National Heart, Lung, and Blood Institute to the CARDIA investigators. GWAS genotyping and quality control for the CARDIA African-Americans was supported by the NHLBI’s Candidate-gene Association REsource (CARe) Study. Statistical analysis of CARDIA data was supported by grants R01-HL084099 and U01-HG004729 to MF. This manuscript has been reviewed by CARDIA for scientific content and consistency of data interpretation with previous CARDIA publications.

The **MESA Family/Air** Studies were conducted and supported by the National Heart, Lung, and Blood Institute (NHLBI) and the United States Environmental Protection Agency (EPA) in collaboration with MESA Family and MESA Air investigators, respectively. Support for MESA Family is provided by grants and contracts R01HL071051, R01HL071205, R01HL071250, R01HL071251, R01HL071252, R01HL071258, and R01HL071259. Support for MESA Air is provided by grant RD83169701. Funding for genotyping was provided by NHLBI Contract N02-HL-6-4278. Genotyping was performed at the Broad Institute of Harvard and MIT (Boston, Massachusetts, USA) and at Affymetrix (Santa Clara, California, USA) using the Affymetrix Genome-Wide Human SNP Array 6.0.

The **GeneSTAR** Study was supported by the National Heart, Lung, and Blood Institute (NHLBI) through the STAMPEED (R01 HL087698-01) consortium as well as grants HL58625-01A1, HL59684, and HL071025-01A1, and a grant from the NIH/National Institute of Nursing Research (NR008153-01). Additional support was provided by a grant from the NIH/National Center for Research Resources (M01-RR000052) to the Johns Hopkins General Clinical Research Center.

The **Genetic Epidemiology Network of Arteriopathy (GENOA)** is supported by the National Institutes of Health, grant numbers HL085571, HL087660, and HL100245 from National Heart, Lung, Blood Institute. We thank Eric Boerwinkle, PhD from the Human Genetics Center and Institute of Molecular Medicine and Division of Epidemiology, University of Texas Health Science Center, Houston, Texas, USA and Julie Cunningham, PhD from the Department of Health Sciences Research, Mayo Clinic College of Medicine, Rochester, MN, USA for their help with genotyping.

MPR is supported by R01-DK071224, R01-DK-090505, U01-HL108636, K24-HL107643 and R01-HL113147.

MK is supported by the AXA Research Fund.

**SUPPLEMENTAL TABLES**

**Supplemental Table S1. Cohort-specific genotyping, imputation, and quality control procedures/criteria.**

|  |  | **CARe COhorts** | | | |  |  |  |
| --- | --- | --- | --- | --- | --- | --- | --- | --- |
|  | **FamHS** | **JHS*** | **CARDIA** | **JHS-ARIC*** | **MESA** | **MESA Family/Air** | **GeneSTAR** | **GENOA** |
| **N analyzed and uploaded** | 596 | 1066 | 671 | 322 | 1646 | 934 | 272 | 316 |
| **Genotyping Platform** | Illumina Human 1M-Duov3 | Affymetrix 6.0 | Affymetrix 6.0 | Affymetrix 6.0 | Affymetrix 6.0 | Affymetrix 6.0 | Illumina Human 1Mv1_c | Affymetrix 6.0 |
| **Calling Algorithm** | BeadStudio | Birdseed v1.33 | Birdseed v1.33 | Birdseed v1.33 | Birdseed v1.33 | Birdseed v2 | BeadStudio | Birdseed v2 |
| **SNP Filters Prior to Imputation:** |  |  |  |  |  |  |  |  |
| **Minimum Allele Frequency (MAF)** | >0.01 | >0.01 | none | >0.01 | >0.01 | none | >0.01 | >0.01 |
| **Call rate** | >0.95 | >0.95 | >0.95 | >0.95 | >0.95 | >0.95 | >0.90 | >0.95 |
| **Hardy-Weinberg Equilibrium (HWE)** | >1E-06 | none | none | none | none | none | >1E-06 | None |
| **Number SNPs used for Imputation** | 745,148 | 868,969 | 839,912 | 796,384 | 881,666 | 861,568 | 687,132 | 550,325 |
| **Imputation Program** | MaCH (version 1.0.16) | MaCH (version 1.0.16) | MaCH (version 1.0.16) | MaCH (version 1.0.16) | MaCH (version 1.0.16) | Impute(version 2.1.0) | MaCH | MaCH (version 1.0.16) |
| **HapMap build used for imputation** | release 22, build 36 | release 22, build 36 | release 22, build 36 | release 22, build 36 | release 22, build 36 | release 22, build 36 | release 22, build 36 | release 22, build 36 |
| **HapMap Reference population used for imputation** | CEU + YRI | CEU + YRI | CEU + YRI | CEU + YRI | CEU + YRI | CEU + YRI + CHB + JPT | CEU + YRI | CEU + YRI |
| **Uploaded SNPs** | 2,199,259 | 2,785,760 | 2,657,131 | 2,768,171 | 2,795,792 | 3,156,527 | 2,507,625 | 2,391,096 |
| **SNPs in Meta-analysis** | 2,180,777 | 2,727,966 | 1,940,353 | 2,663,021 | 2,709,011 | 2,517,622 | 2,346,841 | 2,323,768 |
| **Program used to obtain PCs** | EigenSTRAT | EigenSTRAT | EigenSTRAT | EigenSTRAT | EigenSTRAT | custom code | EigenSTRAT | EigenSTRAT |
| **Ten PCs forced into model?** | Yes | Yes | Yes | Yes | Yes | No | Yes | Yes |
| **Used hybrid dataset?** | Yes | Yes | Yes | Yes | Yes | Yes | Yes | Yes |
| **Were filters used on results prior to upload?** | Yes | No | No | No | No | Yes | No | No |
| **If Used Filters, define them:** |  |  |  |  |  |  |  |  |
| **HWE** | no | no | No | no | no | no | no | no |
| **MAF** | <1% | no | No | no | no | <1% | no | no |
| **r2hat/imputation quality** | no | no | No | no | no | no | no | no |
| **call rate** | no | no | No | no | no | no | no | no |
| **GWAS analysis** |  |  |  |  |  |  |  |  |
| **Statistical program** | SAS/R | Plink/SNPTest | ProABEL | Plink/SNPTest | Plink/SNPTest | R | R | R |
| **Procedure/ analytic model** | Mixed model with kinship matrix | LME/GEE | Linear/logistic model | Linear/Logistic model | Linear/Logistic model | GEE | mixed model with kinship matrix | Linear mixed effects model adjusted for sibship |
| ***Other information*** | Forced 10 PC's into model | Forced 10 PCs into model | Forced 10 PC’s into model | Forced 10 PCs into model | Forced 10 PCs into model | No | forced 10 PCs into model | Forced 10 PC’s |

*JHS CAC data comprised of the JHS *de novo* recruited sample “JHS” (n=1066) and the JHS sample previously enrolled in ARIC study, denoted “JHS-ARIC” (n=322). The JHS-de novo recruited sample was genotyped as a batch via the CARe study at the Broad Institute. Genotyping of all AA ARIC participants also was performed as a separate batch via the CARe study at the Broad Institute. The recommendation from the CARe study analysis committee was to analyze the” JHS” and “JHS-ARIC” individuals separately because QC of JHS and ARIC genotype data was not 100% identical.

**Supplemental Table S2. AA CAC meta-analysis SNP ‘top hits’ and their assessment in the CHARGe EA CAC meta-analysis[**[**12**](#_ENREF_12)**].**

|  |  |  |  |  | **AA Meta-Analysis Results (n=5,823)** | | | | | | **EA CHARGE Look-Up (n=9,992)** | | | | | | |
| --- | --- | --- | --- | --- | --- | --- | --- | --- | --- | --- | --- | --- | --- | --- | --- | --- | --- |
| **SNP** | **Chrom** | **Position** | **Closest Gene** | **Role†** | **Coded Allele** | **Coded Allele Freq** | **Effect** | **SE‡** | **p‡** | **Direction of point estimate for the association§** | **Proxy** | **Coded Allele** | **Coded Allele Freq** | **Effect** | **SE** | **p** | **Direction of point estimate for the association§** |
| rs749924 | 2 | 243026495 | C2orf85 |  | T | 0.50 | -0.19 | 0.04 | 1.072E-07 | +------- | N/A | T | 0.12 | -0.03 | 0.05 | 0.57 | --+-- |
| rs616082 | 18 | 29367640 | MCART2 |  | T | 0.12 | -0.28 | 0.06 | 5.012E-07 | ------?- | N/A | T | 0.02 | -0.05 | 0.20 | 0.79 | +---+ |
| rs1937579 | 6 | 154077193 | C6orf10 |  | T | 0.85 | 0.19 | 0.04 | 2.57E-06 | +-+-+++- | N/A | T | 0.67 | 0.03 | 0.03 | 0.39 | +++-+ |
| rs6929568 | 6 | 8228942 | EEF1E1 |  | T | 0.49 | -0.17 | 0.04 | 2.884E-06 | -------+ | N/A | T | 0.67 | -0.01 | 0.03 | 0.86 | ----+ |
| rs9328448 | 6 | 8001117 | MUTED |  | A | 0.75 | 0.17 | 0.04 | 3.02E-06 | +-+++++- | N/A | A | 0.83 | 0.01 | 0.04 | 0.80 | -+++- |
| rs12552818 | 9 | 104623359 | GRIN3A |  | A | 0.23 | -0.19 | 0.04 | 3.162E-06 | +-----+- | N/A | A | 0.19 | -0.04 | 0.05 | 0.36 | +--++ |
| rs17772222 | 14 | 88826482 | SPATA7 |  | A | 0.81 | -0.21 | 0.04 | 3.236E-06 | -------- | N/A | A | 0.74 | -0.01 | 0.03 | 0.84 | -++-- |
| rs16976171 | 18 | 40059621 | RIT2 |  | T | 0.11 | -0.20 | 0.04 | 3.631E-06 | ++-+---- | N/A | T | 0.00 | -0.02 | 0.30 | 0.95 | -+-++ |
| rs4869804 | 6 | 154062658 | C6orf10 |  | T | 0.85 | 0.19 | 0.04 | 3.631E-06 | +-+-+++- | N/A | T | 0.67 | 0.03 | 0.03 | 0.42 | +++-+ |
| rs11825259 | 11 | 33806822 | FBXO3 |  | A | 0.88 | 0.21 | 0.05 | 3.89E-06 | ?+++++?? | no proxy |  |  |  |  |  |  |
| rs17124700 | 14 | 88905629 | SPATA7 |  | T | 0.19 | 0.21 | 0.04 | 3.89E-06 | ++++++++ | N/A | T | 0.26 | 0.01 | 0.03 | 0.86 | +--++ |
| rs7550636 | 1 | 192806241 | RGS2 |  | T | 0.88 | -0.25 | 0.06 | 4.169E-06 | -------- | N/A | T | 0.63 | -0.03 | 0.03 | 0.40 | -+--- |
| rs11046430 | 12 | 22566429 | SLC2A13 |  | T | 0.12 | -0.21 | 0.04 | 4.266E-06 | ------+- | N/A | T | 0.09 | -0.05 | 0.05 | 0.39 | ++-+- |
| rs2327037 | 6 | 8228490 | EEF1E1 |  | A | 0.51 | 0.16 | 0.04 | 4.786E-06 | +++++++- | N/A | A | 0.33 | 0.01 | 0.03 | 0.74 | +++-- |
| rs9506514 | 13 | 21131211 | IFT88 |  | A | 0.53 | -0.17 | 0.04 | 4.898E-06 | ---+-+-- | N/A | A | 0.66 | -0.01 | 0.03 | 0.88 | +--++ |
| rs12589480 | 14 | 88881270 | SPATA7 | intron | T | 0.19 | 0.20 | 0.04 | 5.012E-06 | ++++++++ | N/A | T | 0.26 | 0.00 | 0.03 | 0.94 | +--++ |
| rs17088339 | 18 | 71476133 | FBXO15 |  | A | 0.02 | -0.68 | 0.15 | 5.495E-06 | --?--?-- | N/A | A | 0.14 | -0.02 | 0.06 | 0.71 | --+-+ |
| rs11054731 | 12 | 12376465 | LRP6 | intron | A | 0.89 | 0.21 | 0.05 | 5.623E-06 | ++++++-- | N/A | A | 0.52 | 0.00 | 0.03 | 0.94 | -++-+ |
| rs10266254 | 7 | 89763864 | STEAP1 |  | A | 0.96 | -0.61 | 0.14 | 6.31E-06 | ?-?--??? | no proxy |  |  |  |  |  |  |
| rs17015535 | 2 | 128502585 | WDR33 | intron | A | 0.91 | -0.38 | 0.09 | 6.457E-06 | ?-?---?? | no proxy |  |  |  |  |  |  |
| rs2622633 | 8 | 106509975 | ZFPM2 | intron | A | 0.21 | 0.20 | 0.05 | 6.457E-06 | +++-++-+ | N/A | A | 0.35 | 0.04 | 0.04 | 0.32 | ++--+ |
| rs7070038 | 10 | 59724388 | IPMK |  | A | 0.11 | -0.21 | 0.05 | 6.607E-06 | -----+-- | rs7905373 | A | 0.08 | -0.40 | 0.27 | 0.13 | ----+ |
| rs10256141 | 7 | 89802053 | STEAP1 |  | T | 0.04 | 0.61 | 0.14 | 6.761E-06 | ?+?++??? | no proxy |  |  |  |  |  |  |
| rs7246657 | 19 | 37747108 | ZNF383 |  | T | 0.53 | -0.16 | 0.04 | 6.761E-06 | -----+-- | N/A | T | 0.81 | -0.04 | 0.04 | 0.37 | -++-+ |
| rs8028579 | 15 | 48005470 | SEMA6D |  | T | 0.11 | 0.30 | 0.07 | 6.761E-06 | ++++++++ | N/A | T | 0.02 | 0.24 | 0.14 | 0.08 | +-+++ |
| rs11760067 | 6 | 135094272 | ALDH8A1 |  | T | 0.02 | 0.80 | 0.18 | 7.244E-06 | ++?++??+ | N/A | T | 0.12 | 0.08 | 0.05 | 0.10 | -+++- |
| rs10803016 | 1 | 242274908 | PLD5 | intron | T | 0.52 | -0.16 | 0.04 | 7.413E-06 | +------- | N/A | T | 0.46 | -0.01 | 0.03 | 0.74 | +---+ |
| rs5754891 | 22 | 34602188 | LARGE |  | A | 0.70 | 0.16 | 0.04 | 7.413E-06 | ++++++++ | N/A | A | 0.53 | 0.01 | 0.03 | 0.85 | ++-++ |
| rs7561462 | 2 | 84686757 | SUCLG1 | near-gene-5 | A | 0.86 | 0.18 | 0.04 | 7.586E-06 | -++++++- | N/A | A | 0.59 | 0.00 | 0.03 | 0.92 | +-++- |
| rs11777747 | 8 | 142466821 | FLJ43860 | intron | T | 0.03 | -0.68 | 0.15 | 7.943E-06 | ?-?--??- | N/A | T | 0.18 | -0.03 | 0.04 | 0.46 | ----+ |
| rs12318506 | 12 | 75718423 | CAPS2 | intron | T | 0.18 | 0.21 | 0.05 | 7.943E-06 | +++++--+ | N/A | T | 0.06 | 0.02 | 0.07 | 0.75 | +-+-+ |
| rs978152 | 8 | 133369094 | KCNQ3 | intron | C | 0.25 | 0.19 | 0.04 | 7.943E-06 | -+++++-+ | N/A | C | 0.67 | 0.00 | 0.03 | 0.89 | --+-- |
| rs11921014 | 3 | 141376140 | LOC646730 |  | A | 0.02 | 1.07 | 0.24 | 8.128E-06 | ?+??+??+ | N/A | A | 0.13 | 0.06 | 0.08 | 0.42 | +++-- |
| rs738956 | 22 | 34601332 | LARGE |  | A | 0.70 | 0.16 | 0.04 | 8.128E-06 | ++++++++ | N/A | A | 0.53 | 0.01 | 0.03 | 0.84 | ++-++ |
| rs12291756 | 11 | 33797814 | FBXO3 | near-gene-5 | C | 0.11 | -0.20 | 0.05 | 8.511E-06 | ?-----?- | no proxy |  |  |  |  |  |  |
| rs1462872 | 8 | 31794109 | NRG1 | intron | A | 0.56 | -0.16 | 0.04 | 8.913E-06 | -------- | N/A | A | 0.17 | -0.10 | 0.05 | 0.05 | ++--- |
| rs1022749 | 9 | 104595776 | GRIN3A |  | T | 0.66 | 0.15 | 0.03 | 9.12E-06 | +++-++-+ | N/A | T | 0.83 | 0.03 | 0.05 | 0.45 | -+++- |
| rs12588287 | 14 | 92536959 | ATXN3 | intron | T | 0.83 | 0.19 | 0.04 | 9.333E-06 | ++++++++ | N/A | T | 0.70 | 0.06 | 0.03 | 0.10 | ++-++ |
| rs10086706 | 8 | 121948408 | SNTB1 |  | T | 0.48 | 0.15 | 0.03 | 9.55E-06 | ++++++++ | N/A | T | 0.66 | 0.02 | 0.03 | 0.50 | ++-++ |
| rs10502575 | 18 | 29336591 | MCART2 |  | A | 0.83 | 0.22 | 0.04 | 1.445E-07 | ++++++-+ | N/A | A | 0.98 | -0.11 | 0.11 | 0.33 | --+-- |
| rs7092929 | 10 | 3538794 | KLF6 |  | A | 0.26 | 0.21 | 0.04 | 3.388E-07 | +++-++++ | N/A | A | 0.21 | -0.03 | 0.04 | 0.40 | +---+ |
| rs741013 | 3 | 64292637 | PRICKLE2 |  | A | 0.98 | -0.97 | 0.19 | 5.129E-07 | ?-?--??? | N/A | A | 0.92 | 0.04 | 0.06 | 0.51 | +++-+ |
| rs8089491 | 18 | 29343254 | MCART2 |  | A | 0.12 | -0.23 | 0.05 | 8.913E-07 | ---+--+? | N/A | A | 0.14 | 0.08 | 0.12 | 0.46 | ++-++ |
| rs17404667 | 3 | 64274552 | PRICKEL2 |  | C | 0.97 | -0.88 | 0.18 | 9.55E-07 | ?-?--??? | N/A | C | 0.92 | 0.04 | 0.06 | 0.49 | -++-+ |
| rs9907236 | 17 | 69490073 | SOX9 |  | A | 0.06 | 0.51 | 0.11 | 1.738E-06 | ++??+++? | N/A | A | 0.05 | -0.30 | 0.14 | 0.03 | --+-- |
| rs7581224 | 2 | 84709215 | SUCLG1 |  | T | 0.14 | -0.20 | 0.04 | 1.778E-06 | +------- | N/A | T | 0.41 | 0.00 | 0.03 | 1.00 | -+--+ |
| rs11651708 | 17 | 64517313 | PRKCA | intron | A | 0.33 | -0.16 | 0.03 | 2.818E-06 | ------+- | N/A | A | 0.36 | 0.05 | 0.03 | 0.12 | -+++- |
| rs3894944 | 4 | 7159702 | SORCS2 |  | A | 0.77 | 0.18 | 0.04 | 3.02E-06 | ++++++++ | N/A | A | 0.77 | -0.01 | 0.04 | 0.84 | +--++ |
| rs11653643 | 17 | 64515731 | PRKCA | intron | A | 0.35 | -0.16 | 0.03 | 3.162E-06 | ------+- | N/A | A | 0.37 | 0.06 | 0.03 | 0.05 | ++++- |
| rs959509 | 10 | 3536296 | KLF6 |  | T | 0.71 | -0.18 | 0.04 | 3.631E-06 | ---+---- | N/A | T | 0.71 | 0.05 | 0.04 | 0.18 | -+++- |
| rs1993293 | 15 | 100297663 | LYSMD4 |  | A | 0.73 | 0.17 | 0.04 | 3.715E-06 | ++++++++ | N/A | A | 0.61 | -0.06 | 0.03 | 0.06 | ---+- |
| rs2679073 | 15 | 87977476 | AGBL1 |  | A | 0.99 | -1.50 | 0.33 | 4.169E-06 | ????-??- | N/A | A | 0.96 | 0.02 | 0.08 | 0.79 | -++++ |
| rs34014631 | 10 | 103698705 | C10orf76 | intron | A | 0.06 | -0.47 | 0.10 | 4.266E-06 | --?--?-- | rs17697908 | T | 0.89 | 0.02 | 0.07 | 0.79 | +++-- |
| rs4900022 | 14 | 90615513 | KCNK13 | intron | A | 0.93 | 0.45 | 0.10 | 4.467E-06 | ++?++++? | N/A | A | 0.99 | -0.31 | 0.17 | 0.07 | ----- |
| rs7765175 | 6 | 113665327 | LOC100652953 |  | T | 0.14 | -0.21 | 0.05 | 4.677E-06 | -------- | N/A | T | 0.38 | 0.03 | 0.03 | 0.26 | +-++- |
| rs7586540 | 2 | 6052751 | ERBB4 |  | A | 0.94 | 0.43 | 0.09 | 5.248E-06 | ++?++-++ | N/A | A | 0.85 | -0.07 | 0.05 | 0.13 | -+--- |
| rs7097515 | 10 | 3539690 | KLF6 |  | A | 0.29 | 0.18 | 0.04 | 6.918E-06 | +++-++++ | N/A | A | 0.21 | -0.03 | 0.04 | 0.41 | +---+ |
| rs2332267 | 1 | 181426910 | CACNA1E |  | T | 0.05 | -0.42 | 0.09 | 7.586E-06 | --?+--+- | N/A | T | 0.10 | 0.02 | 0.05 | 0.65 | +++-- |
| rs4293540 | 2 | 6060989 | ERBB4 |  | T | 0.05 | -0.50 | 0.11 | 7.943E-06 | --?--?+- | N/A | T | 0.07 | 0.04 | 0.06 | 0.52 | +-+-+ |
| rs11079321 | 17 | 55747416 | MSI2 | intron | A | 0.28 | -0.16 | 0.04 | 8.128E-06 | -------+ | N/A | A | 0.29 | 0.01 | 0.04 | 0.80 | ++-++ |
| rs6782380 | 3 | 55184213 | CACNA2D3 |  | C | 0.80 | 0.18 | 0.04 | 8.128E-06 | +++-++++ | N/A | C | 0.80 | -0.01 | 0.04 | 0.79 | -+-++ |
| rs10057565 | 5 | 26928066 | CDH9 | intron | T | 0.83 | 0.20 | 0.04 | 8.318E-06 | +++++-+- | N/A | T | 0.88 | -0.04 | 0.06 | 0.57 | +++-- |
| rs7856675 | 9 | 4555305 | SLC1A1 | intron | A | 0.82 | 0.22 | 0.05 | 8.318E-06 | +-+++++- | N/A | A | 0.92 | -0.06 | 0.07 | 0.35 | ----+ |
| rs9303509 | 17 | 64530887 | PRKCA | intron | A | 0.27 | -0.17 | 0.04 | 8.913E-06 | ------+- | N/A | A | 0.36 | 0.05 | 0.03 | 0.12 | -+++- |
| rs4820834 | 22 | 30751627 | CCDC157 | near-gene-5 | A | 0.02 | -0.71 | 0.16 | 9.12E-06 | --?--?-? | N/A | A | 0.18 | 0.02 | 0.05 | 0.66 | -+++- |
| rs899435 | 12 | 13452780 | EMP1 |  | A | 0.56 | -0.15 | 0.03 | 9.12E-06 | ---+---- | N/A | A | 0.57 | 0.01 | 0.03 | 0.75 | ++--+ |
| rs1602300 | 3 | 14914699 | FGD5 | intron | A | 0.86 | -0.32 | 0.07 | 9.55E-06 | ?-?---?- | rs1627256 | A | 0.17 | 0.01 | 0.05 | 0.77 | +-+++ |

†If no role indicated, then is outside known gene boundaries.

‡SE=standard error; p=p-value.

§ Order of studies: for AA Meta-analysis: FamHS, JHS, CARDIA, JHS-ARIC, MESA, MESA Family/Air, GeneSTAR, GENOA; and for EA CHARGE: Age, Gene/Environment Susceptibility Study—Reykjavik (AGES), Erasmus Rotterdam Study-II, Framingham Heart Study, GENOA, Rotterdam Study-I. GWAS results from each study were completed independently, thus data availability varied by study depending on study specific imputation quality and genotyping quality control for each SNP. Therefore not all studies had results for all SNPs, and when a study did not have a specific SNP a ‘?’ is given for direction.

**Supplemental Table S3. Assessment in African-Americans of SNPs previously associated with CAC in the CHARGe EA CAC Meta-Analysis[**[**12**](#_ENREF_12)**].**

|  |  |  |  |  | **EA CHARGE CAC Meta-Analysis Results (n=9,992)** | | | | | | **AA CAC Meta-Analysis Assessment (n=5,823)** | | | | | | | |
| --- | --- | --- | --- | --- | --- | --- | --- | --- | --- | --- | --- | --- | --- | --- | --- | --- | --- | --- |
| **SNP** | **Chrom** | **Position** | **Closest Gene** | **Role†** | **Coded Allele** | **Coded Allele Freq** | **Effect** | **SE‡** | **p‡** | **Direction of point estimate for the association§** | **Coded Allele** | **Coded Allele Freq** | **Effect** | **SE** | **p** | **Direction of point estimate for the association §** | **HetChiSq‡** | **Het p‡** |
| rs1333049$\parallel$ | 9 | 22125503 | CDKN2B |  | C | 0.48 | 0.27 | 0.03 | 7.58E-19 | +++++ | C | 0.25 | 0.04 | 0.04 | 0.34 | ++++---+ | 5.95 | 0.55 |
| rs4977575$\parallel$ | 9 | 22124744 | CDKN2B |  | C | 0.52 | -0.27 | 0.03 | 9.93E-19 | ----- | C | 0.12 | -0.09 | 0.05 | 0.08 | ---+---+ | 3.95 | 0.79 |
| rs10757278 | 9 | 22124477 | CDKN2B |  | A | 0.52 | -0.27 | 0.03 | 1.19E-18 | ----- | A | 0.80 | -0.05 | 0.04 | 0.27 | -----+++ | 4.15 | 0.76 |
| rs1333047 | 9 | 22124504 | CDKN2B |  | A | 0.52 | -0.27 | 0.03 | 1.20E-18 | ----- | A | 0.12 | -0.07 | 0.05 | 0.18 | ---+--++ | 5.40 | 0.61 |
| rs1333048 | 9 | 22125347 | CDKN2B |  | A | 0.51 | -0.25 | 0.03 | 4.63E-17 | ----- | A | 0.70 | -0.03 | 0.04 | 0.49 | -+--++-- | 8.82 | 0.27 |
| rs10738610 | 9 | 22123766 | CDKN2B |  | A | 0.50 | -0.25 | 0.03 | 1.04E-16 | ----- | A | 0.79 | -0.04 | 0.04 | 0.3 | -----+++ | 4.75 | 0.69 |
| rs1333046 | 9 | 22124123 | CDKN2B |  | A | 0.50 | 0.25 | 0.03 | 1.04E-16 | +++++ | A | 0.26 | 0.02 | 0.04 | 0.66 | +++-+--- | 11.41 | 0.12 |
| rs10116277 | 9 | 22081397 | CDKN2B |  | T | 0.48 | 0.22 | 0.03 | 1.54E-16 | +++-+ | T | 0.88 | 0.02 | 0.05 | 0.76 | -++-+-+- | 7.75 | 0.35 |
| rs1333043 | 9 | 22106731 | CDKN2B |  | A | 0.50 | 0.25 | 0.03 | 2.36E-16 | +++++ | A | 0.88 | 0.08 | 0.05 | 0.14 | ++++-++- | 4.83 | 0.68 |
| rs1537373 | 9 | 22103341 | CDKN2B |  | T | 0.52 | -0.25 | 0.03 | 2.70E-16 | ----- | T | 0.12 | -0.07 | 0.05 | 0.16 | +---+--+ | 5.10 | 0.65 |
| rs7859727 | 9 | 22102165 | CDKN2B |  | T | 0.48 | 0.25 | 0.03 | 2.71E-16 | +++++ | T | 0.74 | 0.02 | 0.04 | 0.63 | ++----+- | 5.98 | 0.54 |
| rs1556516 | 9 | 22100176 | CDKN2B |  | C | 0.48 | 0.24 | 0.03 | 2.72E-16 | +++++ | C | 0.88 | 0.07 | 0.05 | 0.18 | -+++-++- | 5.25 | 0.63 |
| rs1537371 | 9 | 22099568 | CDKN2B |  | A | 0.48 | 0.24 | 0.03 | 2.73E-16 | +++++ | A | 0.88 | 0.05 | 0.05 | 0.29 | -+++-+-- | 5.46 | 0.60 |
| rs2891168 | 9 | 22098619 | CDKN2B |  | A | 0.52 | -0.24 | 0.03 | 2.75E-16 | ----- | A | 0.80 | -0.02 | 0.04 | 0.62 | --+--+++ | 4.95 | 0.67 |
| rs4977574 | 9 | 22098574 | CDKN2B |  | A | 0.52 | -0.24 | 0.03 | 3.21E-16 | ----- | A | 0.81 | -0.02 | 0.04 | 0.65 | -----+++ | 5.72 | 0.57 |
| rs7859362 | 9 | 22105927 | CDKN2B |  | T | 0.50 | -0.24 | 0.03 | 4.06E-16 | ----- | T | 0.12 | -0.08 | 0.05 | 0.13 | ----+--+ | 4.99 | 0.66 |
| rs1333045 | 9 | 22119195 | CDKN2B |  | T | 0.47 | -0.25 | 0.03 | 4.21E-16 | ----- | T | 0.52 | -0.005 | 0.03 | 0.87 | ---+-+-+ | 3.84 | 0.80 |
| rs1412834 | 9 | 22110131 | CDKN2B |  | T | 0.50 | -0.24 | 0.03 | 4.31E-16 | ----- | T | 0.12 | -0.08 | 0.05 | 0.14 | ----+--+ | 4.84 | 0.68 |
| rs7341786 | 9 | 22112241 | CDKN2B |  | A | 0.49 | -0.24 | 0.03 | 4.48E-16 | ----- | A | 0.11 | -0.07 | 0.05 | 0.17 | ---+---+ | 4.38 | 0.73 |
| rs10511701 | 9 | 22112599 | CDKN2B |  | T | 0.49 | -0.24 | 0.03 | 4.48E-16 | ----- | T | 0.28 | -0.07 | 0.04 | 0.09 | +---+--+ | 17.09 | *0.02* |
| rs10738609 | 9 | 22114495 | CDKN2B |  | A | 0.50 | -0.24 | 0.03 | 4.50E-16 | ----- | A | 0.79 | -0.05 | 0.04 | 0.26 | -----+?+ | 3.77 | 0.71 |
| rs10733376 | 9 | 22114469 | CDKN2B |  | C | 0.50 | 0.24 | 0.03 | 4.50E-16 | +++++ | C | 0.88 | 0.08 | 0.05 | 0.13 | ++++-++- | 4.73 | 0.69 |
| rs1333042 | 9 | 22103813 | CDKN2B |  | A | 0.51 | -0.24 | 0.03 | 4.54E-16 | ----- | A | 0.12 | -0.09 | 0.05 | 0.09 | +---+--- | 3.86 | 0.80 |
| rs1537375 | 9 | 22116071 | CDKN2B |  | T | 0.50 | -0.24 | 0.03 | 5.06E-16 | ----- | T | 0.33 | -0.08 | 0.04 | 0.03 | +------+ | 10.39 | 0.17 |
| rs1004638 | 9 | 22115589 | CDKN2B |  | A | 0.50 | -0.24 | 0.03 | 5.28E-16 | ----- | A | 0.12 | -0.06 | 0.05 | 0.28 | ---+--++ | 5.9 | 0.55 |
| rs2383207 | 9 | 22115959 | CDKN2B |  | A | 0.50 | -0.24 | 0.03 | 5.62E-16 | ----- | A | 0.12 | -0.08 | 0.05 | 0.13 | ---+---+ | 4.8 | 0.68 |
| rs1537374 | 9 | 22116046 | CDKN2B |  | A | 0.50 | -0.24 | 0.03 | 6.03E-16 | ----- | A | 0.12 | -0.08 | 0.05 | 0.13 | ---+---+ | 4.8 | 0.68 |
| rs10757272 | 9 | 22088260 | CDKN2B |  | T | 0.48 | 0.24 | 0.03 | 1.80E-15 | +++++ | T | 0.22 | 0.003 | 0.04 | 0.95 | --+++--+ | 6.7 | 0.46 |
| rs10738607 | 9 | 22088094 | CDKN2B |  | A | 0.52 | -0.24 | 0.03 | 4.12E-15 | ----- | A | 0.76 | -0.005 | 0.04 | 0.91 | -+---+++ | 5.25 | 0.63 |
| rs6475606$\parallel$ | 9 | 22081850 | CDKN2B |  | T | 0.48 | 0.22 | 0.03 | 1.64E-13 | +++-+ | T | 0.88 | 0.03 | 0.05 | 0.52 | -+++--+- | 6.28 | 0.51 |
| rs1333050 | 9 | 22125913 | CDKN2B |  | T | 0.67 | 0.25 | 0.03 | 2.82E-13 | +++++ | T | 0.20 | 0.02 | 0.05 | 0.69 | +++++--- | 4.75 | 0.69 |
| rs10757269 | 9 | 22072264 | CDKN2B |  | A | 0.52 | -0.22 | 0.03 | 5.53E-13 | ----- | A | 0.16 | -0.05 | 0.05 | 0.28 | +--+---+ | 16.13 | *0.02* |
| rs9632884 | 9 | 22072301 | CDKN2B |  | C | 0.48 | 0.22 | 0.03 | 5.92E-13 | +++++ | C | 0.90 | 0.005 | 0.05 | 0.93 | -+++-+-- | 5.78 | 0.57 |
| rs10811647 | 9 | 22065002 | CDKN2B |  | C | 0.58 | -0.21 | 0.03 | 6.98E-12 | ----- | C | 0.81 | -0.03 | 0.05 | 0.52 | ----+-++ | 15.27 | *0.03* |
| rs10811650 | 9 | 22067593 | CDKN2B |  | A | 0.58 | -0.21 | 0.03 | 7.30E-12 | ----- | A | 0.80 | -0.01 | 0.04 | 0.85 | ----+-++ | 15.36 | *0.03* |
| rs9349379 | 6 | 12903957 | PHACTR1 | intron | A | 0.59 | -0.21 | 0.03 | 2.65E-11 | ---+- | A | 0.90 | -0.14 | 0.08 | 0.07 | -+?+--++ | 11.61 | 0.07 |
| rs6475608 | 9 | 22101702 | CDKN2B |  | T | 0.31 | -0.20 | 0.03 | 1.11E-09 | ----- | T | 0.06 | -0.03 | 0.11 | 0.81 | ?-?-++?? | 3.39 | 0.34 |
| rs3218020 | 9 | 21997872 | CDKN2A |  | A | 0.34 | 0.19 | 0.03 | 2.53E-09 | +++++ | A | 0.15 | 0.15 | 0.05 | 0 | +++-++-- | 7.23 | 0.41 |
| rs10738604 | 9 | 22025493 | CDKN2B |  | A | 0.36 | 0.18 | 0.03 | 7.40E-09 | +++++ | A | 0.09 | 0.12 | 0.08 | 0.13 | ++?-+--- | 11.94 | 0.06 |
| rs7857345 | 9 | 22087473 | CDKN2B |  | T | 0.31 | -0.18 | 0.03 | 2.64E-08 | ----- | T | 0.07 | -0.05 | 0.09 | 0.54 | +-?--+-+ | 9.68 | 0.14 |
| rs7865618 | 9 | 22031005 | CDKN2B |  | A | 0.58 | 0.17 | 0.03 | 3.11E-08 | +++++ | A | 0.92 | 0.02 | 0.09 | 0.85 | ?+?--+?- | 4.89 | 0.30 |
| rs1008878 | 9 | 22036112 | CDKN2B |  | T | 0.59 | 0.17 | 0.03 | 3.39E-08 | +++++ | T | 0.90 | 0.03 | 0.06 | 0.56 | -++-++-- | 10.22 | 0.18 |
| rs1556515 | 9 | 22036367 | CDKN2B |  | T | 0.59 | 0.17 | 0.03 | 4.02E-08 | +++++ | T | 0.90 | 0.05 | 0.06 | 0.43 | -++-++-- | 9.39 | 0.23 |
| rs2157719 | 9 | 22033366 | CDKN2B |  | T | 0.58 | 0.17 | 0.03 | 4.30E-08 | +++++ | T | 0.92 | 0.09 | 0.09 | 0.3 | -+?-++-? | 5.24 | 0.39 |
| rs1333037 | 9 | 22040765 | CDKN2B |  | T | 0.58 | 0.17 | 0.03 | 5.38E-08 | +++++ | T | 0.92 | 0.15 | 0.09 | 0.12 | ?+?-++?? | 1.65 | 0.65 |
| rs634537 | 9 | 22032152 | CDKN2B |  | T | 0.59 | 0.17 | 0.03 | 5.46E-08 | +++++ | T | 0.92 | 0.04 | 0.08 | 0.65 | -+?+++-- | 6.17 | 0.40 |
| rs1360589 | 9 | 22045317 | CDKN2B |  | T | 0.58 | 0.17 | 0.03 | 5.54E-08 | +++++ | T | 0.92 | 0.15 | 0.09 | 0.11 | ?+?-++?? | 1.22 | 0.75 |
| rs944801 | 9 | 22051670 | CDKN2B |  | C | 0.58 | 0.16 | 0.03 | 5.78E-08 | +++++ | C | 0.92 | 0.03 | 0.09 | 0.74 | ?+?+-+?? | 3.71 | 0.30 |
| rs3217992 | 9 | 22003223 | CDKN2B | utr-3 | T | 0.38 | 0.17 | 0.03 | 5.82E-08 | +++++ | T | 0.14 | 0.03 | 0.05 | 0.58 | +++--+-+ | 20.12 | *0.01* |
| rs679038 | 9 | 22029080 | CDKN2B |  | A | 0.41 | -0.17 | 0.03 | 5.94E-08 | ----- | A | 0.08 | -0.15 | 0.09 | 0.11 | ?-?+-+?? | 2.04 | 0.56 |
| rs7030641 | 9 | 22054040 | CDKN2B |  | T | 0.58 | 0.16 | 0.03 | 6.08E-08 | +++++ | T | 0.92 | 0.15 | 0.09 | 0.1 | ?+?-++?? | 1.16 | 0.76 |
| rs2026458 | 6 | 12825874 | PHACTR1 | intron | T | 0.46 | 0.16 | 0.03 | 1.78E-07 | +++-+ | T | 0.10 | 0.04 | 0.07 | 0.62 | +-?-++-+ | 6.06 | 0.42 |
| rs2184061 | 9 | 22061562 | CDKN2B |  | A | 0.61 | 0.16 | 0.03 | 2.66E-07 | +++++ | A | 0.61 | 0.01 | 0.04 | 0.83 | -+++---- | 7.45 | 0.38 |
| rs1537378 | 9 | 22061614 | CDKN2B |  | A | 0.39 | -0.16 | 0.03 | 2.68E-07 | ----- | A | 0.07 | -0.13 | 0.10 | 0.19 | ?-?+-+?? | 1.88 | 0.60 |
| rs615552 | 9 | 22026077 | CDKN2B |  | T | 0.58 | 0.16 | 0.03 | 2.75E-07 | +++++ | T | 0.92 | 0.15 | 0.09 | 0.1 | ?+?+++?? | 1.62 | 0.66 |
| rs1333039 | 9 | 22065657 | CDKN2B |  | C | 0.61 | 0.16 | 0.03 | 2.92E-07 | +++++ | C | 0.66 | 0.01 | 0.04 | 0.83 | -+++-+-- | 6.69 | 0.46 |
| rs10965224 | 9 | 22067276 | CDKN2B |  | A | 0.61 | 0.16 | 0.03 | 2.99E-07 | +++++ | A | 0.64 | 0.02 | 0.04 | 0.65 | -+++-+-+ | 5.30 | 0.62 |
| rs10811651 | 9 | 22067830 | CDKN2B |  | A | 0.61 | 0.16 | 0.03 | 3.07E-07 | +++++ | A | 0.64 | 0.0001 | 0.04 | 1 | -+++-+-- | 6.42 | 0.49 |
| rs10807323 | 6 | 12795031 | PHACTR1 | intron | A | 0.44 | 0.16 | 0.03 | 3.54E-07 | +++-+ | A | 0.11 | 0.10 | 0.06 | 0.06 | +-+-++++ | 6.73 | 0.46 |
| rs4977756 | 9 | 22068652 | CDKN2B |  | A | 0.61 | 0.16 | 0.03 | 4.02E-07 | +++++ | A | 0.64 | 0.004 | 0.04 | 0.91 | -+++-+-- | 5.54 | 0.59 |
| rs10811641 | 9 | 22014137 | CDKN2B |  | C | 0.64 | -0.16 | 0.03 | 4.12E-07 | ----- | C | 0.85 | -0.01 | 0.05 | 0.85 | ---++-++ | 18.36 | *0.01* |
| rs2069416 | 9 | 22010004 | CDKN2B | near-gene-5 | A | 0.35 | 0.16 | 0.03 | 5.19E-07 | +++++ | A | 0.17 | 0.13 | 0.05 | 0.01 | +++++??- | 3.95 | 0.56 |
| rs2069418 | 9 | 22009698 | CDKN2B | near-gene-5 | C | 0.55 | 0.15 | 0.03 | 7.39E-07 | +++++ | C | 0.91 | 0.10 | 0.09 | 0.26 | ?+?--+?? | 4.08 | 0.25 |
| rs523096 | 9 | 22019129 | CDKN2B |  | A | 0.57 | 0.15 | 0.03 | 7.65E-07 | +++++ | A | 0.92 | 0.06 | 0.09 | 0.49 | ?+?--+?- | 4.74 | 0.32 |
| rs4711863 | 6 | 12915417 | PHACTR1 | intron | C | 0.36 | -0.16 | 0.03 | 9.31E-07 | ---+- | C | 0.20 | -0.01 | 0.04 | 0.87 | -+-+-+-- | 0.39 | 1.00 |
| rs2327620 | 6 | 12907591 | PHACTR1 | intron | A | 0.36 | -0.15 | 0.03 | 1.16E-06 | ---+- | A | 0.73 | -0.03 | 0.04 | 0.5 | -++--+++ | 5.84 | 0.56 |
| rs3809346 | 13 | 110960943 | COL4A1 | near-gene-5 | A | 0.43 | 0.15 | 0.03 | 1.25E-06 | +-+++ | A | 0.38 | 0.004 | 0.04 | 0.93 | -++----+ | 0.75 | 1.00 |
| rs4773144 | 13 | 110960712 | COL4A1 | near-gene-5 | A | 0.57 | -0.15 | 0.03 | 1.65E-06 | -+--- | A | 0.62 | -0.004 | 0.04 | 0.91 | +--+-++- | 0.56 | 1.00 |
| rs3731239 | 9 | 21974218 | CDKN2A | intron | A | 0.60 | 0.16 | 0.03 | 2.12E-06 | +++++ | A | 0.94 | 0.06 | 0.10 | 0.56 | -+?-++-? | 5.70 | 0.34 |
| rs7750679 | 6 | 12891301 | PHACTR1 | intron | T | 0.36 | -0.15 | 0.03 | 2.34E-06 | ---+- | T | 0.49 | -0.06 | 0.03 | 0.08 | ---+--+- | 4.16 | 0.76 |
| rs10120688 | 9 | 22056499 | CDKN2B |  | A | 0.49 | 0.14 | 0.03 | 2.41E-06 | +++++ | A | 0.42 | 0.07 | 0.03 | 0.05 | -+++-+-+ | 10.29 | 0.17 |
| rs1332844 | 6 | 12889004 | PHACTR1 | intron | T | 0.64 | 0.15 | 0.03 | 2.61E-06 | +++-+ | T | 0.54 | 0.03 | 0.03 | 0.32 | -+++++-- | 6.85 | 0.45 |
| rs9395214 | 6 | 12898884 | PHACTR1 | intron | C | 0.36 | -0.15 | 0.03 | 2.73E-06 | ---+- | C | 0.80 | -0.03 | 0.04 | 0.47 | -+-+-+-? | 16.80 | *0.01* |
| rs7751826 | 6 | 12900977 | PHACTR1 | intron | T | 0.64 | 0.15 | 0.03 | 2.75E-06 | +++-+ | T | 0.35 | 0.02 | 0.04 | 0.62 | -++-+--+ | 3.26 | 0.86 |
| rs9369640 | 6 | 12901441 | PHACTR1 | intron | A | 0.64 | 0.15 | 0.03 | 2.84E-06 | +++-+ | A | 0.35 | 0.02 | 0.04 | 0.66 | -++-+--+ | 3.79 | 0.80 |
| rs9296512 | 6 | 12894904 | PHACTR1 | intron | C | 0.64 | 0.15 | 0.03 | 2.97E-06 | +++-+ | C | 0.49 | 0.03 | 0.03 | 0.4 | --+-+++- | 6.02 | 0.54 |
| rs3218009 | 9 | 21998757 | CDKN2A |  | C | 0.82 | 0.20 | 0.04 | 3.27E-06 | +++++ | C | 0.98 | 0.15 | 0.20 | 0.46 | ?+?--??? | 4.48 | 0.11 |
| rs518394 | 9 | 22019673 | CDKN2B |  | C | 0.41 | -0.15 | 0.03 | 3.37E-06 | ----+ | C | 0.08 | -0.04 | 0.09 | 0.66 | +-?++-?+ | 7.03 | 0.22 |
| rs6783981 | 3 | 167528129 | SERPINI1 | intron | T | 0.50 | -0.14 | 0.03 | 3.94E-06 | ----- | T | 0.25 | -0.03 | 0.04 | 0.46 | +-----++ | 2.54 | 0.92 |
| rs17676451 | 12 | 96375785 | HAL | intron | A | 0.22 | -0.17 | 0.04 | 4.08E-06 | ----- | A | 0.06 | -0.09 | 0.09 | 0.35 | --?-+--- | 10.67 | 0.10 |
| rs6604023 | 1 | 91944897 | CDC7 |  | C | 0.18 | 0.18 | 0.04 | 4.29E-06 | +++++ | C | 0.41 | 0.03 | 0.04 | 0.42 | ++++-?-+ | 2.52 | 0.87 |
| rs1014342 | 6 | 12923157 | PHACTR1 | intron | T | 0.37 | -0.14 | 0.03 | 4.71E-06 | ---+- | T | 0.80 | -0.03 | 0.04 | 0.43 | -+-+-+++ | 15.03 | *0.04* |
| rs8180558 | 6 | 12919989 | PHACTR1 | intron | T | 0.37 | -0.14 | 0.03 | 4.81E-06 | ---+- | T | 0.29 | -0.004 | 0.04 | 0.91 | ++---++- | 1.11 | 0.99 |
| rs2383206 | 9 | 22115026 | CDKN2B |  | A | 0.50 | -0.24 | 0.03 | 4.51E-16 | ----- | A | 0.57 | 0.02 | 0.04 | 0.62 | -++-+++- | 10.86 | 0.14 |
| rs944797 | 9 | 22115286 | CDKN2B |  | T | 0.50 | -0.24 | 0.03 | 5.44E-16 | ----- | T | 0.57 | 0.03 | 0.04 | 0.46 | -+++-++- | 9.78 | 0.20 |
| rs1537370 | 9 | 22084310 | CDKN2B |  | T | 0.48 | 0.23 | 0.03 | 1.26E-14 | +++++ | T | 0.68 | -0.02 | 0.04 | 0.6 | -++----+ | 4.65 | 0.70 |
| rs1333040 | 9 | 22083404 | CDKN2B |  | T | 0.56 | 0.21 | 0.03 | 1.67E-12 | +++++ | T | 0.62 | -0.01 | 0.04 | 0.83 | -++-+--- | 9.56 | 0.21 |
| rs564398 | 9 | 22029547 | CDKN2B |  | T | 0.59 | 0.17 | 0.03 | 4.38E-08 | +++++ | T | 0.93 | -0.02 | 0.09 | 0.81 | -+?----- | 6.35 | 0.38 |
| rs543830 | 9 | 22026639 | CDKN2B |  | A | 0.59 | 0.17 | 0.03 | 6.34E-08 | +++++ | A | 0.92 | -0.002 | 0.20 | 1 | ?????-?? | 0 | 1 |
| rs1412829 | 9 | 22043926 | CDKN2B |  | A | 0.59 | 0.16 | 0.03 | 6.63E-08 | +++++ | A | 0.93 | -0.01 | 0.09 | 0.89 | -+?+-+-? | 5.89 | 0.32 |
| rs1063192 | 9 | 22003367 | CDKN2B | utr-3 | A | 0.58 | 0.16 | 0.03 | 7.89E-08 | +++++ | A | 0.92 | -0.01 | 0.09 | 0.89 | -+?+---? | 7.33 | 0.20 |
| rs8181050 | 9 | 22064391 | CDKN2B |  | A | 0.61 | 0.16 | 0.03 | 2.87E-07 | +++++ | A | 0.93 | -0.03 | 0.22 | 0.87 | ?????-?? | 0 | 1 |
| rs7454157 | 6 | 12909874 | PHACTR1 | intron | A | 0.36 | -0.16 | 0.03 | 1.14E-06 | ---+- | A | 0.58 | 0.01 | 0.04 | 0.69 | +-+---++ | 4.86 | 0.68 |
| rs8181047 | 9 | 22064465 | CDKN2B |  | A | 0.31 | -0.15 | 0.03 | 2.15E-06 | ----- | A | 0.05 | 0.01 | 0.11 | 0.95 | ?-?+++?- | 2.81 | 0.59 |
| rs4714955 | 6 | 12903435 | PHACTR1 | intron | T | 0.36 | -0.15 | 0.03 | 2.73E-06 | ---+- | T | 0.14 | 0.02 | 0.05 | 0.65 | +++++-+- | 6.11 | 0.53 |
| rs2327621 | 6 | 12922689 | PHACTR1 | intron | A | 0.63 | 0.14 | 0.03 | 3.95E-06 | +++-+ | A | 0.62 | -0.003 | 0.03 | 0.93 | +--+---+ | 2.08 | 0.96 |
| rs8001186 | 13 | 110376855 | IRS2 |  | T | 0.67 | -0.15 | 0.03 | 4.51E-06 | ---+- | T | 0.60 | 0.03 | 0.04 | 0.46 | +--++-+- | 23.78 | *0.001* |
| rs2876303 | 6 | 12919867 | PHACTR1 | intron | A | 0.63 | 0.14 | 0.03 | 4.78E-06 | +++-+ | A | 0.27 | -0.03 | 0.04 | 0.37 | --++---+ | 4.50 | 0.72 |
| rs9381500 | 6 | 12922535 | PHACTR1 | intron | A | 0.63 | 0.14 | 0.03 | 4.81E-06 | +++-+ | A | 0.65 | -0.0006 | 0.04 | 0.98 | +-+++--- | 1.92 | 0.96 |
| rs1953088 | 6 | 12925936 | PHACTR1 | intron | A | 0.63 | 0.14 | 0.03 | 4.97E-06 | +++-+ | A | 0.22 | -0.08 | 0.04 | 0.05 | +-++---- | 10.60 | 0.16 |

†If no role indicated, then is outside known gene boundaries.

‡SE=standard error; p=p-value; HetChiSq=Heterozygosity Chi-Square statistic; Hetp=Heterozygosity p-value.

§ Order of studies: for AA Meta-analysis: FamHS, JHS, CARDIA, JHS-ARIC, MESA, MESA Family/Air, GeneSTAR, GENOA; and for EA CHARGE: Age, Gene/Environment Susceptibility Study—Reykjavik (AGES), Erasmus Rotterdam Study-II, Framingham Heart Study, GENOA, Rotterdam Study-I. GWAS results from each study were completed independently, thus data availability varied by study depending on study specific imputation quality and genotyping quality control for each SNP. Therefore not all studies had results for all SNPs, and when a study did not have a specific SNP a ‘?’ is given for direction.

$\parallel$ Top SNPs from prior studies for EA CAC (rs1333049)[[12](#_ENREF_12)], EA CAD/MI (rs4977574)[[26](#_ENREF_26)], and AA CHD (rs6475606)[[19](#_ENREF_19)].

SNPs below horizontal lines have effects in opposite directions between the EA CAC meta-analysis and the AA CAC meta-analysis.

**Supplemental Table S4. Assessment in African-Americans of loci previously associated with coronary artery disease.**

|  |  |  | **Prior CAD loci in EA Studies** | | | | | **AA CAC Meta-Analysis Results (n=5,823)** | | | | | | | |
| --- | --- | --- | --- | --- | --- | --- | --- | --- | --- | --- | --- | --- | --- | --- | --- |
| **SNP** | **Chrom†** | **Closest Gene** | **Coded Allele** | **Coded Allele Freq** | **beta or OR (95% CI)†** | **p†** | **Source** | **Coded Allele** | **Coded Allele Freq** | **Effect** | | **SE†** | **Direction of point estimate for the association ‡** | **p** | **hetp†** |
| rs10953541 | 7q22 |  | C | 0.8 | 1.10 (1.05-1.15) | 1.33E-05 | C4D novel loci[[27](#_ENREF_27)] | C | 0.94 | 0.05 | | 0.10 | +-?-+-+? | 0.65 | 0.47 |
| rs1122608 | 19p13.2 | LDLR | G | 0.77 | 1.14 (1.09; 1.18) | 9.73E-10 | CARDIoGRAM[[26](#_ENREF_26)];  Established up to 2010 | G | 0.94 | 0.12 | | 0.10 | ?-?--+?+ | 0.25 | 0.50 |
| rs11556924 | 7q32.2 | ZC3HC1 | C | 0.62 | 1.09 (1.07; 1.12) | 9.18E-18 | CARDIoGRAM novel loci[[26](#_ENREF_26)] | C | 0.92 | 0.04 | | 0.08 | -+?--++- | 0.59 | 0.49 |
| rs12413409 | 10q24.32 | CYP17A1, CNNM2, NT5C2 | G | 0.89 | 1.12 (1.08; 1.16) | 1.03E-09 | CARDIoGRAM novel loci[[26](#_ENREF_26)] | G | 0.93 | 0.14 | | 0.09 | +-?----+ | 0.12 | 0.84 |
| rs12936587 | 17p11.2 | RASD1, SMCR3, PEMT | G | 0.56 | 1.07 (1.05; 1.09) | 4.45E-10 | CARDIoGRAM novel loci[[26](#_ENREF_26)] | G | 0.71 | 0.06 | | 0.04 | ++--+--- | 0.09 | 0.29 |
| rs1412444 | 10 | LIPA | T | 0.42 | 1.08 (1.05-1.12) | 1.0E-05 | C4D novel loci[[27](#_ENREF_27)] | T | 0.40 | 0.04 | | 0.04 | -++-+-+- | 0.30 | 0.98 |
| rs17114036 | 1p32.2 | PPAP2B | A | 0.91 | 1.17 (1.13; 1.22) | 3.81E-19 | CARDIoGRAM novel loci[[26](#_ENREF_26)] | A | 0.85 | 0.01 | | 0.05 | -++-+++- | 0.85 | 0.48 |
| rs17321515 | 8q24.13 | TRIB1 | A | 0.52 |  | 6.5E-07 | IBC 50K CAD | A | 0.45 | -0.03 | | 0.03 | -++----+ | 0.39 | 0.20 |
| rs1746048 | 10q11.21 | CXCL12 | C | 0.87 | 1.09 (1.07; 1.13) | 2.12E-10 | CARDIoGRAM[[26](#_ENREF_26)];  Established up to 2010 | C | 0.55 | 0.06 | | 0.03 | +----+++ | 0.08 | 0.25 |
| rs17609940 | 6p21.31 | ANKS1A | G | 0.75 | 1.07 (1.05; 1.10) | 1.36E-08 | CARDIoGRAM novel loci[[26](#_ENREF_26)] | G | 0.95 | 0.12 | | 0.12 | ?-?---?+ | 0.31 | 0.41 |
| rs216172 | 17p13.3 | SMG6, SRR | C | 0.37 | 1.07 (1.05; 1.09) | 1.15E-09 | CARDIoGRAM novel loci[[26](#_ENREF_26)] | C | 0.36 | 0.01 | | 0.04 | ?-++--?+ | 0.76 | 0.51 |
| rs2246942 | 10q23.31 | LIPA | G | 0.36 |  | 4.3E-09 | IBC 50K CAD | G | 0.41 | 0.05 | | 0.03 | ---+-+-+ | 0.19 | 0.75 |
| rs2306374 | 3q22.3 | MRAS | C | 0.18 | 1.12 (1.07; 1.16) | 3.34E-08 | CARDIoGRAM[[26](#_ENREF_26)];  Established up to 2010 | C | 0.06 | 0.04 | | 0.09 | --?-+-++ | 0.63 | 0.93 |
| rs2706399 | 5q31.1 | IL5 | G | 0.51 |  | 2.1E-06 | IBC 50K CAD | G | 0.21 | -0.04 | | 0.04 | ++++++-+ | 0.31 | 0.57 |
| rs2895811 | 14q32.2 | HHIPL1 | C | 0.43 | 1.07 (1.05; 1.10) | 1.14E-10 | CARDIoGRAM novel loci[[26](#_ENREF_26)] | C | 0.21 | 0.004 | | 0.05 | ++-?++-? | 0.93 | 0.13 |
| rs3825807 | 15q25.1 | ADAMTS7 | A | 0.57 | 1.08 (1.06; 1.10) | 1.07E-12 | CARDIoGRAM novel loci[[26](#_ENREF_26)] | A | 0.85 | 0.05 | | 0.05 | ++++-+?- | 0.32 | 0.19 |
| rs4299376 | 2p21 | ABCG8 | G | 0.29 |  | 1.4E-06 | IBC 50K CAD | G | 0.16 | 0.07 | | 0.05 | ---++-+- | 0.13 | 0.64 |
| rs4380028 | 15 | ADAMTS7-MORF4L1 | C | 0.65 | 1.07 (1.03-1.11) | 3.32E-04 | C4D novel loci[[27](#_ENREF_27)] | C | 0.78 | 0.01 | | 0.04 | +-+--++- | 0.79 | 0.18 |
| rs4773144 | 13q34 | COL4A1, COL4A2 | G | 0.44 | 1.07 (1.05; 1.09) | 3.84E-09 | CARDIoGRAM novel loci[[26](#_ENREF_26)] | G | 0.38 | 0.004 | | 0.04 | +--+-++- | 0.91 | 1.00 |
| rs4977574 | 9p21.3 | CDKN2A/B, ANRIL | G | 0.46 | 1.29 (1.23; 1.36) | 1.35E-22 | CARDIoGRAM[[26](#_ENREF_26)];  Established up to 2010 | G | 0.19 | 0.02 | | 0.04 | -----+++ | 0.65 | 0.57 |
| rs579459 | 9q34.2 | ABO | C | 0.21 | 1.10 (1.07; 1.13) | 4.08E-14 | CARDIoGRAM novel loci[[26](#_ENREF_26)] | C | 0.13 | 0.03 | | 0.05 | +---+-+? | 0.54 | 0.65 |
| rs6725887 | 2q33.1 | WDR12 | C | 0.15 | 1.14 (1.09; 1.19) | 1.12E-09 | CARDIoGRAM[[26](#_ENREF_26)];  Established up to 2010 | C | 0.03 | 0.02 | | 0.13 | +-?--?++ | 0.89 | 0.88 |
| rs974819 | 11 | PDGFD | T | 0.32 | 1.09 (1.05-1.13) | 1.5E-05 | C4D novel loci[[27](#_ENREF_27)] | T | 0.49 | 0.03 | | 0.04 | ++-+-+++ | 0.46 | 0.52 |
| rs17465637 | 1q41 | MIA3 | C | 0.74 | 1.14 (1.09; 1.20) | 1.36E-08 | CARDIoGRAM[[26](#_ENREF_26)];  Established up to 2010 | C | 0.28 | 0.06 | | 0.05 | ?+?---?- | 0.25 | 0.48 |
| rs599839 | 1p13.3 | SORT1 | A | 0.78 | 1.11 (1.08; 1.15) | 2.89E-10 | CARDIoGRAM[[26](#_ENREF_26)];  Established up to 2010 | A | 0.28 | 0.07 | | 0.04 | ++++++-- | 0.06 | 0.92 |
| rs11206510 | 1p32.3 | PCSK9 | T | 0.82 | 1.08 (1.05; 1.11) | 9.10E-08 | CARDIoGRAM[[26](#_ENREF_26)];  Established up to 2010 | T | 0.87 | -0.09 | | 0.05 | -+---+-- | 0.07 | 0.71 |
| rs12190287 | 6q23.2 | TCF21 | C | 0.62 | 1.08 (1.06; 1.10) | 1.07E-12 | CARDIoGRAM novel loci[[26](#_ENREF_26)] | C | 0.90 | -0.01 | | 0.08 | ++?-+--- | 0.91 | 0.28 |
| rs12526453 | 6p24.1 | PHACTR1 | C | 0.67 | 1.10 (1.06; 1.13) | 1.15E-09 | CARDIoGRAM[[26](#_ENREF_26)];  Established up to 2010 | C | 0.84 | -0.005 | | 0.04 | +--+++-- | 0.91 | 0.16 |
| rs2505083 | 10 | KIAA1462 | C | 0.38 | 1.08 (1.05-1.12) | 8.78E-06 | C4D novel loci[[27](#_ENREF_27)] | C | 0.11 | -0.03 | | 0.05 | +-++-+-+ | 0.63 | 0.11 |
| rs3184504 | 12q24.12 | SH2B3 | T | 0.44 | 1.07 (1.04;1.10) | 6.35E-06 | CARDIoGRAM[[26](#_ENREF_26)];  Established up to 2010 | T | 0.09 | -0.02 | | 0.09 | ++?----? | 0.79 | *0.009* |
| rs3798220 | 6q25.3 | LPA | C | 0.02 | 1.51 (1.33; 1.70) | 3E-11 | CARDIoGRAM[[26](#_ENREF_26)];  Established up to 2010 |  |  | |  |  |  |  |  |
| rs46522 | 17q21.32 | UBE2Z, GIP, ATP5G1, SNF8 | T | 0.53 | 1.06 (1.04; 1.08) | 1.81E-08 | CARDIoGRAM novel loci[[26](#_ENREF_26)] | T | 0.14 | -0.02 | | 0.05 | +++-+--+ | 0.69 | *0.0003* |
| rs964184 | 11q23.3 | ZNF259, APOA5-A4-C3-A1 | G | 0.13 | 1.13 (1.10; 1.16) | 1.02E-17 | CARDIoGRAM novel loci[[26](#_ENREF_26)] | G | 0.21 | -0.08 | | 0.04 | ++++++++ | 0.07 | 0.88 |
| rs9982601 | 21q22.11 | MRPS6/gene-desert/KCNE2 | T | 0.15 | 1.18 (1.12; 1.24) | 4.22E-10 | CARDIoGRAM[[26](#_ENREF_26)];  Established up to 2010 | T | 0.21 | -0.04 | | 0.05 | -+----+- | 0.38 | 0.80 |

†Chrom=chromosome; OR= odds ratio; CI=confidence interval; p=p-value; SE=standard error; hetp=heterozygosity p-value.

‡Order of studies: FamHS, JHS, CARDIA, JHS-ARIC, MESA, MESA Family/Air, GeneSTAR, GENOA. GWAS results from each study were completed independently, thus data availability varied by study depending on study specific imputation quality and genotyping quality control for each SNP. Therefore not all studies had results for all SNPs, and when a study did not have a specific SNP a ‘?’ is given for direction.

SNPs below horizontal line have effect in the opposite direction.

**Supplemental Table S5. SNP signals within EA and AA LD blocks at the 9p21 region as defined by CARe AA CHD GWAS*.**

| **SNP** | **Chrom†** | **Position** | **Closest Gene** | **Role‡** | **Coded Allele** | **Coded Allele Freq** | **Effect** | **SE†** | **Direction of point estimate for the association §** | **p†** | **het p†** |
| --- | --- | --- | --- | --- | --- | --- | --- | --- | --- | --- | --- |
| rs3218020 | 9 | 21997872 | CDKN2A |  | A | 0.15 | 0.15 | 0.05 | +++-++-- | ***0.003*** | 0.41 |
| rs2811712 | 9 | 21998035 | CDKN2A |  | A | 0.78 | 0.03 | 0.04 | ++++-+-- | 0.49 | 0.39 |
| rs3218018 | 9 | 21998139 | CDKN2A |  | T | 0.90 | -0.001 | 0.07 | -+?-++-- | 0.98 | 0.81 |
| rs3218009 | 9 | 21998757 | CDKN2A |  | C | 0.98 | 0.15 | 0.20 | ?+?--??? | 0.46 | 0.11 |
| rs3218005 | 9 | 22000247 | CDKN2B |  | T | 0.78 | 0.06 | 0.04 | +++-++-- | 0.15 | 0.62 |
| rs3218003 | 9 | 22000770 | CDKN2B |  | C | 0.87 | -0.01 | 0.07 | ?+?--+?- | 0.89 | 0.68 |
| rs3218002 | 9 | 22000841 | CDKN2B |  | A | 0.22 | -0.06 | 0.04 | ---+--++ | 0.15 | 0.62 |
| rs3217999 | 9 | 22001572 | CDKN2B |  | A | 0.88 | 0.08 | 0.05 | ?++--+?? | 0.12 | 0.61 |
| rs3217997 | 9 | 22002316 | CDKN2B |  | T | 0.88 | 0.11 | 0.05 | ?+++++?? | **0.03** | 0.98 |
| rs3217994 | 9 | 22002864 | CDKN2B | near-gene-3 | T | 0.88 | 0.10 | 0.05 | ++++++-? | *0.05* | 0.65 |
| rs3217992 | 9 | 22003223 | CDKN2B | utr-3 | T | 0.14 | 0.03 | 0.05 | +++--+-+ | 0.58 | *0.005* |
| rs1063192 | 9 | 22003367 | CDKN2B | utr-3 | A | 0.92 | -0.01 | 0.09 | -+?+---? | 0.89 | 0.20 |
| rs3217989$\parallel$ | 9 | 22003790 | CDKN2B | utr-3 | T | 0.76 | -0.01 | 0.04 | --+--++? | 0.87 | 0.81 |
| rs3217986 | 9 | 22005330 | CDKN2B | utr-3 | T | 0.98 | 0.08 | 0.15 | ++?--?+- | 0.62 | 0.73 |
| rs2069426 | 9 | 22006273 | CDKN2B | intron | T | 0.10 | 0.01 | 0.07 | --?-+-++ | 0.89 | 0.86 |
| rs974336 | 9 | 22006348 | CDKN2B | intron | T | 0.25 | 0.12 | 0.12 | +?????+? | 0.32 | 0.35 |
| rs3217980 | 9 | 22006607 | CDKN2B | intron | A | 0.12 | -0.10 | 0.05 | ------+? | **0.049** | 0.65 |
| rs2069423 | 9 | 22007771 | CDKN2B | intron | A | 0.98 | -0.29 | 0.40 | ??????-? | 0.47 | 1 |
| rs2069422 | 9 | 22008026 | CDKN2B | intron | T | 0.90 | 0.01 | 0.07 | -+?-++-- | 0.89 | 0.81 |
| rs2069418 | 9 | 22009698 | CDKN2B | near-gene-5 | C | 0.91 | 0.10 | 0.09 | ?+?--+?? | 0.26 | 0.25 |
| rs3217973 | 9 | 22009960 | CDKN2B | near-gene-5 | A | 0.05 | -0.07 | 0.11 | --?+-?-- | 0.55 | 0.60 |
| rs2069416 | 9 | 22010004 | CDKN2B | near-gene-5 | A | 0.17 | 0.13 | 0.05 | +++++??- | ***0.009*** | 0.56 |
| rs495490 | 9 | 22010412 | CDKN2B | near-gene-5 | A | 0.98 | 0.12 | 0.20 | ?+?+-??? | 0.55 | 0.47 |
| rs575427 | 9 | 22011477 | CDKN2B |  | A | 0.98 | 0.10 | 0.20 | ?+?+-??- | 0.62 | 0.53 |
| rs573687 | 9 | 22011642 | CDKN2B |  | A | 0.07 | -0.14 | 0.09 | +-?+-++? | 0.14 | 0.25 |
| rs13298881 | 9 | 22012051 | CDKN2B |  | T | 0.97 | -0.17 | 0.13 | --?--?+- | 0.22 | 0.32 |
| rs16935753 | 9 | 22012229 | CDKN2B |  | A | 0.12 | -0.11 | 0.05 | ?-----?? | **0.03** | 0.96 |
| rs545226 | 9 | 22012422 | CDKN2B |  | A | 0.90 | -0.02 | 0.06 | ----+-++ | 0.71 | *0.02* |
| rs7032979 | 9 | 22012457 | CDKN2B |  | T | 0.20 | -0.02 | 0.05 | ?+----?? | 0.63 | 0.65 |
| rs10811640 | 9 | 22013411 | CDKN2B |  | T | 0.70 | 0.08 | 0.03 | -++-++-- | **0.03** | 0.24 |
| rs10811641 | 9 | 22014137 | CDKN2B |  | C | 0.85 | -0.009 | 0.05 | ---++-++ | 0.85 | *0.01* |
| rs2106120 | 9 | 22017101 | CDKN2B |  | T | 0.70 | 0.07 | 0.03 | -++-++-- | **0.03** | 0.18 |
| rs2106119 | 9 | 22017550 | CDKN2B |  | A | 0.30 | -0.07 | 0.03 | +--+--++ | **0.05** | 0.12 |
| rs643319 | 9 | 22017836 | CDKN2B |  | A | 0.27 | -0.05 | 0.04 | +---+-+- | 0.18 | 0.09 |
| rs7044859 | 9 | 22018781 | CDKN2B |  | A | 0.70 | 0.05 | 0.03 | -+++-+-- | 0.15 | 0.20 |
| rs523096 | 9 | 22019129 | CDKN2B |  | A | 0.92 | 0.06 | 0.09 | ?+?--+?- | 0.49 | 0.32 |
| rs518394 | 9 | 22019673 | CDKN2B |  | C | 0.08 | -0.04 | 0.09 | +-?++-?+ | 0.66 | 0.22 |
| rs10757264 | 9 | 22019732 | CDKN2B |  | A | 0.30 | -0.05 | 0.03 | +---+-++ | 0.17 | 0.11 |
| rs10965212 | 9 | 22023795 | CDKN2B |  | A | 0.70 | 0.04 | 0.03 | -+++-+-- | 0.23 | 0.10 |
| AFFX-SNP_6867479__rs496892 | 9 | 22014351 |  |  | T |  | -0.02 |  | -????-+? | 0.80 | 0.06 |
| rs1292136 | 9 | 22024351 |  |  | T | 0.26 | 0.02 | 0.15 | +??????? | 0.89 | 1 |
| rs496892 | 9 | 22024351 | CDKN2B |  | T | 0.29 | -0.04 | 0.05 | ?-?-+-?- | 0.41 | 0.12 |
| rs10811644 | 9 | 22025067 | CDKN2B |  | A | 0.30 | -0.07 | 0.03 | +--+--++ | **0.03** | 0.26 |
| rs7035484 | 9 | 22025240 | CDKN2B |  | C | 0.30 | -0.07 | 0.03 | +--+--++ | **0.04** | 0.16 |
| rs10738604 | 9 | 22025493 | CDKN2B |  | A | 0.09 | 0.12 | 0.08 | ++?-+--- | 0.13 | 0.06 |
| rs615552 | 9 | 22026077 | CDKN2B |  | T | 0.92 | 0.15 | 0.09 | ?+?+++?? | 0.10 | 0.66 |
| rs1591137 | 9 | 22026483 | CDKN2B |  | A | 0.65 | -0.05 | 0.04 | ?-++--?? | 0.26 | 0.59 |
| rs543830 | 9 | 22026639 | CDKN2B |  | A | 0.92 | -0.002 | 0.20 | ?????-?? | 1 | 1 |
| rs1591136 | 9 | 22026834 | CDKN2B |  | C | 0.71 | 0.03 | 0.04 | -+++-+-- | 0.35 | *0.02* |
| rs598664 | 9 | 22027551 | CDKN2B |  | T | 0.90 | -0.02 | 0.07 | -+?-++-- | 0.72 | 0.58 |
| rs7049105 | 9 | 22028801 | CDKN2B |  | A | 0.29 | -0.03 | 0.03 | +---+-++ | 0.37 | 0.07 |
| rs679038 | 9 | 22029080 | CDKN2B |  | A | 0.08 | -0.15 | 0.09 | ?-?+-+?? | 0.11 | 0.56 |
| rs10965215 | 9 | 22029445 | CDKN2B |  | A | 0.33 | 0.02 | 0.04 | +-++-+-+ | 0.62 | 0.10 |
| rs564398 | 9 | 22029547 | CDKN2B |  | T | 0.93 | -0.02 | 0.09 | -+?----- | 0.81 | 0.38 |
| rs662463 | 9 | 22030438 | CDKN2B |  | A | 0.13 | -0.03 | 0.05 | -----+++ | 0.54 | 0.80 |
| rs7865618 | 9 | 22031005 | CDKN2B |  | A | 0.92 | 0.02 | 0.09 | ?+?--+?- | 0.85 | 0.30 |
| rs10115049 | 9 | 22032119 | CDKN2B |  | A | 0.71 | -0.01 | 0.04 | +---++++ | 0.72 | *0.05* |
| rs634537 | 9 | 22032152 | CDKN2B |  | T | 0.92 | 0.04 | 0.08 | -+?+++-- | 0.65 | 0.40 |
| rs2157719 | 9 | 22033366 | CDKN2B |  | T | 0.92 | 0.09 | 0.09 | -+?-++-? | 0.30 | 0.39 |
| rs1759417 | 9 | 22033389 | CDKN2B |  | T | 0.02 | -0.10 | 0.20 | +-?-+??? | 0.62 | 0.85 |
| rs2151280 | 9 | 22034719 | CDKN2B |  | A | 0.29 | 0.07 | 0.04 | -++-+--? | *0.06* | 0.17 |
| rs1008878 | 9 | 22036112 | CDKN2B |  | T | 0.90 | 0.03 | 0.06 | -++-++-- | 0.56 | 0.18 |
| rs1556515 | 9 | 22036367 | CDKN2B |  | T | 0.90 | 0.05 | 0.06 | -++-++-- | 0.43 | 0.23 |
| rs1333037 | 9 | 22040765 | CDKN2B |  | T | 0.92 | 0.15 | 0.09 | ?+?-++?? | 0.12 | 0.65 |
| rs1360590 | 9 | 22041443 | CDKN2B |  | T | 0.26 | -0.03 | 0.04 | +--++-++ | 0.46 | 0.25 |
| rs17694493 | 9 | 22041998 | CDKN2B |  | C | 0.89 | 0.01 | 0.05 | -+++-+-+ | 0.89 | 0.57 |
| rs12352425 | 9 | 22042086 | CDKN2B |  | A | 0.28 | -0.001 | 0.04 | --+++++- | 0.98 | 0.23 |
| rs1412829 | 9 | 22043926 | CDKN2B |  | A | 0.93 | -0.01 | 0.09 | -+?+-+-? | 0.89 | 0.32 |
| rs1333035 | 9 | 22044059 | CDKN2B |  | A | 0.78 | 0.04 | 0.04 | -+++-+-- | 0.32 | 0.63 |
| rs1333034 | 9 | 22044122 | CDKN2B |  | T | 0.89 | 0.03 | 0.06 | -++-++-- | 0.63 | 0.91 |
| rs1360589 | 9 | 22045317 | CDKN2B |  | T | 0.92 | 0.15 | 0.09 | ?+?-++?? | 0.11 | 0.75 |
| rs1333033 | 9 | 22045653 | CDKN2B |  | T | 0.26 | -0.02 | 0.05 | ?+----?? | 0.65 | 0.82 |
| rs7851706 | 9 | 22047437 | CDKN2B |  | T | 0.12 | 0.04 | 0.07 | ?+?-+-?+ | 0.62 | 0.38 |
| rs7028570 | 9 | 22048683 | CDKN2B |  | A | 0.42 | 0.08 | 0.04 | -++-++-- | **0.03** | 0.24 |
| rs2151279 | 9 | 22049845 | CDKN2B |  | A | 0.88 | 0.07 | 0.05 | ?++--+?? | 0.20 | 0.62 |
| rs944801 | 9 | 22051670 | CDKN2B |  | C | 0.92 | 0.03 | 0.09 | ?+?+-+?? | 0.74 | 0.30 |
| rs11790231 | 9 | 22053591 | CDKN2B |  | A | 0.03 | 0.24 | 0.11 | +-?++?++ | **0.04** | 0.93 |
| rs10965219 | 9 | 22053687 | CDKN2B |  | A | 0.30 | -0.05 | 0.04 | +---+--+ | 0.13 | 0.07 |
| rs17756311 | 9 | 22053895 | CDKN2B |  | A | 0.10 | 0.01 | 0.05 | +---+-++ | 0.89 | 0.83 |
| rs7030641 | 9 | 22054040 | CDKN2B |  | T | 0.92 | 0.15 | 0.09 | ?+?-++?? | 0.10 | 0.76 |
| rs17694572 | 9 | 22054356 | CDKN2B |  | A | 0.10 | -0.01 | 0.06 | +---+-++ | 0.89 | 0.91 |
| rs10120688 | 9 | 22056499 | CDKN2B |  | A | 0.42 | 0.07 | 0.03 | -+++-+-+ | *0.05* | 0.17 |
| rs2184061 | 9 | 22061562 | CDKN2B |  | A | 0.61 | 0.01 | 0.04 | -+++---- | 0.83 | 0.38 |
| rs1537378 | 9 | 22061614 | CDKN2B |  | A | 0.07 | -0.13 | 0.10 | ?-?+-+?? | 0.19 | 0.60 |
| rs1011970 | 9 | 22062134 | CDKN2B |  | T | 0.32 | 0.03 | 0.04 | -++++-+- | 0.36 | 0.48 |
| rs8181050 | 9 | 22064391 | CDKN2B |  | A | 0.93 | -0.03 | 0.22 | ?????-?? | 0.87 | 1 |
| rs8181047 | 9 | 22064465 | CDKN2B |  | A | 0.05 | 0.01 | 0.11 | ?-?+++?- | 0.95 | 0.59 |
| rs10811647 | 9 | 22065002 | CDKN2B |  | C | 0.81 | -0.03 | 0.05 | ----+-++ | 0.52 | *0.03* |
| rs1333039 | 9 | 22065657 | CDKN2B |  | C | 0.66 | 0.01 | 0.04 | -+++-+-- | 0.83 | 0.46 |
| rs10965224 | 9 | 22067276 | CDKN2B |  | A | 0.64 | 0.02 | 0.04 | -+++-+-+ | 0.65 | 0.62 |
| rs10811650 | 9 | 22067593 | CDKN2B |  | A | 0.80 | -0.01 | 0.04 | ----+-++ | 0.85 | *0.03* |
| rs10811651 | 9 | 22067830 | CDKN2B |  | A | 0.64 | 0.0001 | 0.04 | -+++-+-- | 1 | 0.49 |
| rs16905597 | 9 | 22068074 | CDKN2B |  | A | 0.10 | -0.09 | 0.06 | ------+? | *0.10* | 0.89 |
| rs1412831 | 9 | 22068646 | CDKN2B |  | A | 0.77 | 0.01 | 0.04 | --++++-? | 0.89 | 0.71 |
| rs4977756 | 9 | 22068652 | CDKN2B |  | A | 0.64 | 0.004 | 0.04 | -+++-+-- | 0.91 | 0.59 |
| rs16905599 | 9 | 22069144 | CDKN2B |  | A | 0.22 | 0.03 | 0.04 | -+++++-- | 0.39 | 0.13 |
| rs10757269 | 9 | 22072264 | CDKN2B |  | A | 0.16 | -0.05 | 0.05 | +--+---+ | 0.28 | *0.02* |
| rs9632884 | 9 | 22072301 | CDKN2B |  | C | 0.90 | 0.01 | 0.05 | -+++-+-- | 0.93 | 0.57 |
| rs10757270 | 9 | 22072719 | CDKN2B |  | A | 0.67 | -0.05 | 0.05 | ?-?-+-?+ | 0.35 | *0.01* |
| rs17761197 | 9 | 22072730 | CDKN2B |  | T | 0.03 | 0.37 | 0.16 | ?+?-+??- | **0.02** | 0.16 |
| rs16923583 | 9 | 22073334 | CDKN2B |  | A | 0.16 | -0.13 | 0.07 | ?-?+--?- | *0.05* | 0.93 |
| rs7855162 | 9 | 22074793 | CDKN2B |  | T | 0.64 | 0.001 | 0.04 | +++--++? | 0.98 | 0.17 |
| rs1412832 | 9 | 22077543 | CDKN2B |  | T | 0.95 | -0.01 | 0.10 | -+?-+--? | 0.95 | 0.17 |
| rs7855660 | 9 | 22078305 | CDKN2B |  | T | 0.23 | 0.10 | 0.04 | -++++++? | **0.02** | *0.02* |
| rs6475605 | 9 | 22079020 | CDKN2B |  | C | 0.35 | 0.001 | 0.04 | ?--++-?? | 0.98 | 0.06 |
| rs1333048 | 9 | 22125347 | CDKN2B |  | A | 0.70 | -0.03 | 0.04 | -+--++-- | 0.49 | 0.27 |
| rs7858034 | 9 | 22080791 | CDKN2B |  | A | 0.02 | 0.04 | 0.21 | ?-?++??? | 0.85 | 0.60 |
| rs10116277 | 9 | 22081397 | CDKN2B |  | T | 0.88 | 0.02 | 0.05 | -++-+-+- | 0.76 | 0.35 |
| rs6475606$\parallel$ | 9 | 22081850 | CDKN2B |  | T | 0.88 | 0.03 | 0.05 | -+++--+- | 0.52 | 0.51 |
| rs1547704 | 9 | 22082340 | CDKN2B |  | A | 0.16 | -0.15 | 0.07 | ?-?--+?+ | **0.03** | 0.44 |
| rs1547705 | 9 | 22082375 | CDKN2B |  | A | 0.77 | 0.09 | 0.04 | +++++-+- | **0.04** | 0.46 |
| rs10965228 | 9 | 22082380 | CDKN2B |  | A | 0.98 | -0.03 | 0.18 | -+?+-??+ | 0.85 | 0.98 |
| rs7853953 | 9 | 22083017 | CDKN2B |  | A | 0.82 | 0.02 | 0.05 | ?-+++-?? | 0.72 | 0.67 |
| rs1333040 | 9 | 22083404 | CDKN2B |  | T | 0.62 | -0.01 | 0.04 | -++-+--- | 0.83 | 0.21 |
| rs1537370 | 9 | 22084310 | CDKN2B |  | T | 0.68 | -0.02 | 0.04 | -++----+ | 0.60 | 0.7 |
| rs10120722 | 9 | 22086840 | CDKN2B |  | A | 0.27 | 0.13 | 0.04 | ?+++++?+ | ***0.002*** | 0.64 |
| rs7857345 | 9 | 22087473 | CDKN2B |  | T | 0.07 | -0.05 | 0.09 | +-?--+-+ | 0.54 | 0.14 |
| rs10738607 | 9 | 22088094 | CDKN2B |  | A | 0.76 | -0.004 | 0.04 | -+---+++ | 0.91 | 0.63 |
| rs10757272 | 9 | 22088260 | CDKN2B |  | T | 0.25 | 0.003 | 0.04 | --+++--+ | 0.95 | 0.46 |
| rs16905640 | 9 | 22088556 | CDKN2B |  | A | 0.04 | -0.18 | 0.27 | ?-?+???? | 0.49 | 0.12 |
| rs10757274 | 9 | 22096055 | CDKN2B |  | A | 0.76 | 0.06 | 0.15 | +??????? | 0.66 | 1 |
| rs16905644 | 9 | 22097022 | CDKN2B |  | T | 0.11 | -0.25 | 0.06 | ?-----?? | ***4.07E-05*** | 0.34 |
| rs6475607 | 9 | 22097693 | CDKN2B |  | A | 0.29 | 0.05 | 0.04 | -++--+-- | 0.24 | 0.10 |
| rs4977574$\parallel$ | 9 | 22098574 | CDKN2B |  | A | 0.81 | -0.02 | 0.04 | -----+++ | 0.65 | 0.57 |
| rs2891168 | 9 | 22098619 | CDKN2B |  | A | 0.80 | -0.02 | 0.04 | --+--+++ | 0.62 | 0.67 |
| rs1537371 | 9 | 22099568 | CDKN2B |  | A | 0.88 | 0.05 | 0.05 | -+++-+-- | 0.29 | 0.60 |
| rs7856476 | 9 | 22099940 | CDKN2B |  | A | 0.30 | 0.06 | 0.04 | ?+++-+?? | 0.14 | 0.10 |
| rs1556516 | 9 | 22100176 | CDKN2B |  | C | 0.88 | 0.07 | 0.05 | -+++-++- | 0.18 | 0.63 |
| rs10965232 | 9 | 22101120 | CDKN2B |  | T | 0.31 | 0.08 | 0.04 | -++-+++? | **0.04** | 0.13 |
| rs6475608 | 9 | 22101702 | CDKN2B |  | T | 0.06 | -0.03 | 0.11 | ?-?-++?? | 0.81 | 0.34 |
| rs7859727 | 9 | 22102165 | CDKN2B |  | T | 0.74 | 0.02 | 0.04 | ++----+- | 0.63 | 0.54 |
| rs1537373 | 9 | 22103341 | CDKN2B |  | T | 0.12 | -0.07 | 0.05 | +---+--+ | 0.16 | 0.65 |
| rs7022719 | 9 | 22103748 | CDKN2B |  | T | 0.86 | -0.05 | 0.16 | ?????-?? | 0.76 | 1 |
| rs1333042 | 9 | 22103813 | CDKN2B |  | A | 0.12 | -0.09 | 0.05 | +---+--- | *0.09* | 0.80 |
| rs7859362 | 9 | 22105927 | CDKN2B |  | T | 0.12 | -0.08 | 0.05 | ----+--+ | 0.13 | 0.66 |
| rs1333043 | 9 | 22106731 | CDKN2B |  | A | 0.88 | 0.08 | 0.05 | ++++-++- | 0.14 | 0.68 |
| rs10217426 | 9 | 22109387 | CDKN2B |  | C | 0.47 | 0.26 | 0.11 | ?????+?? | **0.02** | 1 |
| rs1412834 | 9 | 22110131 | CDKN2B |  | T | 0.12 | -0.08 | 0.05 | ----+--+ | 0.14 | 0.68 |
| rs7341786 | 9 | 22112241 | CDKN2B |  | A | 0.11 | -0.07 | 0.05 | ---+---+ | 0.17 | 0.73 |
| rs10511701 | 9 | 22112599 | CDKN2B |  | T | 0.28 | -0.07 | 0.04 | +---+--+ | *0.09* | *0.02* |
| rs7032115 | 9 | 22112943 | CDKN2B |  | A | 0.92 | 0.04 | 0.23 | ?????+?? | 0.87 | 1 |
| rs16905652 | 9 | 22113924 | CDKN2B |  | A | 0.13 | 0.07 | 0.06 | ?-++++?? | 0.26 | 0.96 |
| rs10733376 | 9 | 22114469 | CDKN2B |  | C | 0.88 | 0.08 | 0.05 | ++++-++- | 0.13 | 0.69 |
| rs10738609 | 9 | 22114495 | CDKN2B |  | A | 0.79 | -0.05 | 0.04 | -----+?+ | 0.26 | 0.71 |
| rs2383206 | 9 | 22115026 | CDKN2B |  | A | 0.57 | 0.02 | 0.04 | -++-+++- | 0.62 | 0.14 |
| rs10965234 | 9 | 22115078 | CDKN2B |  | T | 0.45 | 0.08 | 0.04 | ?++--+?? | **0.04** | 0.21 |
| rs10965235 | 9 | 22115105 | CDKN2B |  | A | 0.45 | 0.07 | 0.04 | -++++++? | *0.06* | *0.05* |
| rs4990722 | 9 | 22115217 | CDKN2B |  | T | 0.14 | -0.02 | 0.05 | --+--+-? | 0.72 | 0.67 |
| rs944796 | 9 | 22115285 | CDKN2B |  | C | 0.55 | -0.08 | 0.04 | ?--++-?? | **0.05** | 0.21 |
| rs944797 | 9 | 22115286 | CDKN2B |  | T | 0.57 | 0.03 | 0.04 | -+++-++- | 0.46 | 0.20 |
| rs1004638 | 9 | 22115589 | CDKN2B |  | A | 0.12 | -0.06 | 0.05 | ---+--++ | 0.28 | 0.55 |
| rs2383207 | 9 | 22115959 | CDKN2B |  | A | 0.12 | -0.08 | 0.05 | ---+---+ | 0.13 | 0.68 |
| rs1537374 | 9 | 22116046 | CDKN2B |  | A | 0.12 | -0.08 | 0.05 | ---+---+ | 0.13 | 0.68 |
| rs1537375 | 9 | 22116071 | CDKN2B |  | T | 0.33 | -0.08 | 0.04 | +------+ | **0.03** | 0.17 |
| rs17761446 | 9 | 22118102 | CDKN2B |  | T | 0.96 | -0.22 | 0.12 | --?+-?-+ | *0.05* | 0.55 |
| rs7854631 | 9 | 22118378 | CDKN2B |  | A | 0.06 | -0.17 | 0.25 | ?????-?? | 0.50 | 1 |
| rs1333044 | 9 | 22119128 | CDKN2B |  | A | 0.26 | -0.01 | 0.04 | ?-++--?- | 0.85 | 0.97 |
| rs1333045 | 9 | 22119195 | CDKN2B |  | T | 0.52 | -0.01 | 0.04 | ---+-+-+ | 0.87 | 0.80 |
| rs7869069 | 9 | 22123590 | CDKN2B |  | T | 0.83 | -0.04 | 0.05 | ?+-+--?? | 0.40 | 0.56 |
| rs10738610 | 9 | 22123766 | CDKN2B |  | A | 0.79 | -0.04 | 0.04 | -----+++ | 0.30 | 0.69 |
| rs7854016 | 9 | 22123967 | CDKN2B |  | A | 0.82 | -0.11 | 0.05 | ?-----?? | **0.03** | 0.45 |
| rs1333046 | 9 | 22124123 | CDKN2B |  | A | 0.26 | 0.02 | 0.04 | +++-+--- | 0.66 | 0.12 |
| rs10757278 | 9 | 22124477 | CDKN2B |  | A | 0.80 | -0.05 | 0.04 | -----+++ | 0.27 | 0.76 |
| rs1333047 | 9 | 22124504 | CDKN2B |  | A | 0.12 | -0.07 | 0.05 | ---+--++ | 0.18 | 0.61 |
| rs4977575 | 9 | 22124744 | CDKN2B |  | C | 0.12 | -0.09 | 0.05 | ---+---+ | *0.08* | 0.79 |
| rs1333049$\parallel$ | 9 | 22125503 | CDKN2B |  | C | 0.25 | 0.04 | 0.04 | ++++---+ | 0.34 | 0.55 |

*As defined by CARe AA CHD GWAS[[19](#_ENREF_19)]; SNPs below blank row are in the smaller AA fine mapping region; bolded are p-values<0.05; bold italics are p-values<0.01; and bold, italic, and underlined are most significant.

†Chrom=chromosome; SE=Standard error; p=p-value; het p=heterozygosity p-value.

‡If no role indicated, then is outside known gene boundaries.

§Order of studies: FamHS, JHS, CARDIA, JHS-ARIC, MESA, MESA Family/Air, GeneSTAR, GENOA. GWAS results from each study were completed independently, thus data availability varied by study depending on study specific imputation quality and genotyping quality control for each SNP. Therefore not all studies had results for all SNPs, and when a study did not have a specific SNP a ‘?’ is given for direction.

$\parallel$Top SNPs from prior studies for AA CAD (rs3217989)[[28](#_ENREF_28)], AA CHD (rs6475606)[[19](#_ENREF_19)], EA CAD (rs4977574)[[26](#_ENREF_26)], and EA CAC (rs1333049)[[12](#_ENREF_12)].

**Supplemental Table S6. Participant characteristics of the CHARGE EA CAC sample[**[**12**](#_ENREF_12)**].**

| **Characteristic** | **AGES-Reykjavik* (n=3177)** | **Rotterdam Study-II (n=1228)** | **Framingham Heart Study (n=3207)** | **GENOA**  **(n=629)** | **Rotterdam Study-I (n=1720)** |
| --- | --- | --- | --- | --- | --- |
| Age, y | 76.4 (5.5) | 67.2 (6.7) | 52.2 (11.6) | 58.0 (9.8) | 70.7 (5.5) |
| Women, % | 58 | 53 | 49 | 58 | 54 |
| Mean CAC score | 686 (1011) | 312 (712) | 131 (432) | 191 (487) | 505 (969) |
| Maximum CAC score | 8673 | 8636 | 5016 | 4867 | 12,611 |
| Detectable CAC, % | 88.2 | 80.0 | 41.6 | 68.2 | 91.0 |
| CAC score >100, % | 66.7 | 40.5 | 19.0 | 28.3 | 54.4 |
| CAC score >300, % | 48.8 | 25.7 | 10.3 | 14.3 | 36.3 |
| Hypertension, % | 80.1 | 63.8 | 28.0 | 71.2 | 61.9 |
| Diabetes Mellitus, % | 11.5 | 10.2 | 5.2 | 13.4 | 13.7 |
| Current smoker, % | 12.7 | 14.9 | 12.9 | 9.5 | 17.3 |
| Former smoker, % | 45.3 | 54.1 | 34.5 | 34.2 | 54.7 |
| Total Cholesterol, mmol/L | 5.70 (1.17) | 5.70 (0.98) | 5.08 (0.91) | 5.18 (0.88) | 5.83 (0.96) |
| HDL cholesterol, mmol/L | 1.61 (0.47) | 1.45 (0.38) | 1.40 (0.44) | 1.35 (0.41) | 1.40 (0.39) |
| Triglycerides, mmol/L | 1.22 (0.67) | NA | 1.44 (1.01) | 1.80 (1.19) | 1.54 (0.79) |
| Body mass index, kg/m^2^ | 27.1 (4.4) | 27.8 (4.9) | 27.7 (5.3) | 30.7 (6.3) | 27.0 (3.9) |
| Waist Circumference, cm | 101 (12) | 94 (12) | 97 (16) | 101 (16) | 94 (11) |
| Prevalent MI, % | 7.5 | 4.2 | 1.2 | 0 | 7.7 |

* AGES=Age, Gene/Environment Susceptibility Study—Reykjavik, GENOA= Genetic Epidemiology Network of Arteriopathy.

**Supplemental Table S7. Assessment of SNP associations in *PHACTR1* region in the AA CAC Meta-Analysis.**

|  |  |  |  |  | **EA CHARGE CAC Meta-Analysis Results (n=9,992)** | | | | | | **AA CAC Meta-Analysis Assessment (n=5,823)** | | | | | | | |
| --- | --- | --- | --- | --- | --- | --- | --- | --- | --- | --- | --- | --- | --- | --- | --- | --- | --- | --- |
| **SNP** | **Chrom** | **Position** | **Closest Gene** | **Role†** | **Coded Allele** | **Coded Allele Freq** | **Effect** | **SE‡** | **p‡** | **Direction of point estimate for the association§** | **Coded Allele** | **Coded Allele Freq** | **Effect** | **SE** | **p** | **Direction of point estimate for the association §** | **HetISq‡** | **Het p‡** |
| rs9349379 | 6 | 12903957 | PHACTR1 | intron | A | 0.59 | -0.21 | 0.03 | 2.65E-11 | ---+- | A | 0.90 | -0.14 | 0.08 | 0.07 | -+?+--++ | 44.5 | 0.0946 |
| rs2026458 | 6 | 12825874 | PHACTR1 | intron | T | 0.46 | 0.16 | 0.03 | 1.78E-07 | +++-+ | T | 0.10 | 0.04 | 0.07 | 0.62 | +-?-++-+ | 0.3 | 0.4211 |
| rs10807323 | 6 | 12795031 | PHACTR1 | intron | A | 0.44 | 0.16 | 0.03 | 3.54E-07 | +++-+ | A | 0.11 | 0.10 | 0.06 | 0.06 | +-+-++++ | 0 | 0.466 |
| rs4711863 | 6 | 12915417 | PHACTR1 | intron | C | 0.36 | -0.16 | 0.03 | 9.31E-07 | ---+- | C | 0.20 | -0.01 | 0.04 | 0.87 | -+-+-+-- | 0 | 0.9997 |
| rs2327620 | 6 | 12907591 | PHACTR1 | intron | A | 0.36 | -0.15 | 0.03 | 1.16E-06 | ---+- | A | 0.73 | -0.03 | 0.04 | 0.5 | -++--+++ | 0 | 0.5626 |
| rs7750679 | 6 | 12891301 | PHACTR1 | intron | T | 0.36 | -0.15 | 0.03 | 2.34E-06 | ---+- | T | 0.49 | -0.06 | 0.03 | 0.08 | ---+--+- | 0 | 0.7435 |
| rs1332844 | 6 | 12889004 | PHACTR1 | intron | T | 0.64 | 0.15 | 0.03 | 2.61E-06 | +++-+ | T | 0.54 | 0.03 | 0.03 | 0.32 | -+++++-- | 7.1 | 0.375319 |
| rs9395214 | 6 | 12898884 | PHACTR1 | intron | C | 0.36 | -0.15 | 0.03 | 2.73E-06 | ---+- | C | 0.80 | -0.03 | 0.04 | 0.47 | -+-+-+-? | 64.1 | 0.0103 |
| rs7751826 | 6 | 12900977 | PHACTR1 | intron | T | 0.64 | 0.15 | 0.03 | 2.75E-06 | +++-+ | T | 0.35 | 0.02 | 0.04 | 0.62 | -++-+--+ | 0 | 0.77499 |
| rs9369640 | 6 | 12901441 | PHACTR1 | intron | A | 0.64 | 0.15 | 0.03 | 2.84E-06 | +++-+ | A | 0.35 | 0.02 | 0.04 | 0.66 | -++-+--+ | 0 | 0.71367 |
| rs9296512 | 6 | 12894904 | PHACTR1 | intron | C | 0.64 | 0.15 | 0.03 | 2.97E-06 | +++-+ | C | 0.49 | 0.03 | 0.03 | 0.4 | --+-+++- | 0 | 0.4856 |
| rs1014342 | 6 | 12923157 | PHACTR1 | intron | T | 0.37 | -0.14 | 0.03 | 4.71E-06 | ---+- | T | 0.80 | -0.03 | 0.04 | 0.43 | -+-+-+++ | 52.7 | 0.0385 |
| rs8180558 | 6 | 12919989 | PHACTR1 | intron | T | 0.37 | -0.14 | 0.03 | 4.81E-06 | ---+- | T | 0.29 | -0.004 | 0.04 | 0.91 | ++---++- | 0 | 0.99295 |
| rs7454157 | 6 | 12909874 | PHACTR1 | intron | A | 0.36 | -0.16 | 0.03 | 1.14E-06 | ---+- | A | 0.58 | 0.01 | 0.04 | 0.69 | +-+---++ | 0 | 0.6608 |
| rs4714955 | 6 | 12903435 | PHACTR1 | intron | T | 0.36 | -0.15 | 0.03 | 2.73E-06 | ---+- | T | 0.14 | 0.02 | 0.05 | 0.65 | +++++-+- | 0 | 0.51677 |
| rs2327621 | 6 | 12922689 | PHACTR1 | intron | A | 0.63 | 0.14 | 0.03 | 3.95E-06 | +++-+ | A | 0.62 | -0.003 | 0.03 | 0.93 | +--+---+ | 0 | 0.91487 |
| rs2876303 | 6 | 12919867 | PHACTR1 | intron | A | 0.63 | 0.14 | 0.03 | 4.78E-06 | +++-+ | A | 0.27 | -0.03 | 0.04 | 0.37 | --++---+ | 0 | 0.7081 |
| rs9381500 | 6 | 12922535 | PHACTR1 | intron | A | 0.63 | 0.14 | 0.03 | 4.81E-06 | +++-+ | A | 0.65 | -0.0006 | 0.04 | 0.98 | +-+++--- | 0 | 0.9598 |
| rs1953088 | 6 | 12925936 | PHACTR1 | intron | A | 0.63 | 0.14 | 0.03 | 4.97E-06 | +++-+ | A | 0.22 | -0.08 | 0.04 | 0.05 | +-++---- | 31.4 | 0.1773 |
| rs12526453¥ | 6 | 13035530 | PHACTR1 |  | C | 0.65 | 0.11 |  | 1.30E-09 |  | C | 0.84 | -0.007 | 0.04 | 0.87 | +--+++-- | 32.3 | 0.1699 |
|  |  |  |  |  | C | 0.66 | 0.14 | 0.03 | 2.22E-05 | +++-+ |  |  |  |  |  |  |  |  |
| rs7768030 | 6 | 12822973 | PHACTR1 | Intron | A | 0.91 | 0.05 | 0.06 | 0.337 | +++-- | A | 0.82 | -0.13 | 0.05 | 0.004365 | ---+---- | 0 | 0.99 |
| rs3823445 | 6 | 13182995 | PHACTR1 | Intron | A | 0.30 | -0.01 | 0.03 | 0.756 | -+-++ | A | 0.08 | -0.22 | 0.08 | 0.007943 | +-?+---- | 0 | 0.52 |
| rs4714931 | 6 | 12857408 | PHACTR1 | Intron | A | 0.89 | 0.05 | 0.05 | 0.3277 | +++-+ | A | 0.87 | -0.14 | 0.05 | 0.008128 | ---+---+ | 0 | 0.92 |
| rs9381810 | 6 | 13183121 | PHACTR1 | Intron | T | 0.70 | 0.01 | 0.03 | 0.7529 | +-+-- | T | 0.93 | 0.22 | 0.09 | 0.010233 | -+?-++++ | 0 | 0.55 |
| rs1889062 | 6 | 12747362 | PHACTR1 | Intron | NOT AVAILABLE | | | | | | A | 0.13 | -0.13 | 0.05 | 0.010965 | ?----+?? | 61.7 | 0.03 |
| rs9367373€ | 6 | 13193331 | PHACTR1 | Intron | T | 0.96 | -0.03 | 0.09 | 0.7681 | ++--+ | T | 0.86 | -0.18 | 0.07 | 0.010965 | ?-?---?+ | 30.7 | 0.22 |
| rs436268€ | 6 | 13257222 | PHACTR1 | Intron | T | 0.06 | -0.04 | 0.06 | 0.5301 | +---- | T | 0.05 | -0.28 | 0.11 | 0.01122 | ?-?---?- | 0 | 0.65 |
| rs1232382 | 6 | 13258128 | PHACTR1 | Intron | NOT AVAILABLE | | | | | | T | 0.13 | -0.14 | 0.05 | 0.011749 | ?--+---? | 0 | 0.81 |
| rs9369918 | 6 | 13193172 | PHACTR1 | Intron | C | 0.03 | 0.03 | 0.10 | 0.7901 | -+++- | C | 0.08 | 0.21 | 0.08 | 0.013183 | -+?+++++ | 73.6 | 0.0009 |
| rs521960 | 6 | 13251953 | PHACTR1 | Intron | C | 0.09 | -0.05 | 0.05 | 0.3414 | ++--+ | C | 0.24 | 0.10 | 0.04 | 0.014791 | ++++++++ | 0 | 0.9 |
| rs6934634 | 6 | 12885179 | PHACTR1 | Intron | NOT AVAILABLE | | | | | | C | 0.97 | 0.39 | 0.16 | 0.015488 | ?+?++??? | 0 | 0.72 |
| rs1232380 | 6 | 13216619 | PHACTR1 | Intron | NOT AVAILABLE | | | | | | C | 0.97 | 0.42 | 0.18 | 0.016218 | ?+?-+??? | 49.2 | 0.14 |
| rs13191496 | 6 | 12851625 | PHACTR1 | Intron | A | 0.08 | -0.10 | 0.06 | 0.08293 | ----- | A | 0.1 | -0.17 | 0.07 | 0.017378 | --?--+-+ | 0 | 0.70 |
| rs1150622 | 6 | 13248665 | PHACTR1 | Intron | NOT AVAILABLE | | | | | | A | 0.87 | 0.13 | 0.06 | 0.020417 | ?++-++?? | 0 | 0.75 |
| rs13201878 | 6 | 12943066 | PHACTR1 | Intron | A | 0.73 | 0.13 | 0.04 | 0.0002 | +++++ | A | 0.79 | 0.09 | 0.04 | 0.020893 | +++-++-+ | 24.8 | 0.23 |
| rs6916268 | 6 | 12982529 | PHACTR1 | Intron | T | 0.41 | 0.01 | 0.03 | 0.7423 | +-+-+ | T | 0.77 | 0.09 | 0.04 | 0.021878 | -++-+++- | 28.2 | 0.20 |
| rs4380742 | 6 | 12976244 | PHACTR1 | Intron | A | 0.59 | -0.01 | 0.03 | 0.717 | -+-+- | A | 0.22 | -0.09 | 0.04 | 0.022387 | +--+---+ | 25.9 | 0.22 |
| rs9463363 | 6 | 13002629 | PHACTR1 | Intron | A | 0.41 | -0.006 | 0.03 | 0.8353 | +---+ | A | 0.78 | 0.09 | 0.04 | 0.022387 | -+++-++- | 57.1 | 0.02 |
| rs4349811 | 6 | 13007933 | PHACTR1 | Intron | T | 0.41 | -0.008 | 0.03 | 0.7986 | +---+ | T | 0.78 | 0.09 | 0.04 | 0.022387 | -++-+++- | 43.6 | 0.09 |
| rs7774775 | 6 | 12977548 | PHACTR1 | Intron | A | 0.59 | -0.01 | 0.03 | 0.7179 | -+-+- | A | 0.21 | -0.09 | 0.04 | 0.025704 | +--+---+ | 34.8 | 0.15 |
| rs4715048 | 6 | 13006410 | PHACTR1 | Intron | C | 0.59 | 0.006 | 0.03 | 0.8354 | -+++- | C | 0.22 | -0.09 | 0.04 | 0.026303 | ?--+--?+ | 50.7 | 0.07 |
| rs11757262 | 6 | 13210479 | PHACTR1 | Intron | NOT AVAILABLE | | | | | | T | 0.97 | -0.32 | 0.14 | 0.026303 | ?-?+-??? | 50.4 | 0.13 |
| rs3846903 | 6 | 13281412 | PHACTR1 | Intron | A | 0.41 | 0.008 | 0.03 | 0.8013 | +-+-- | A | 0.64 | -0.08 | 0.04 | 0.026303 | ----+--- | 0 | 0.53 |
| rs6902050 | 6 | 12892236 | PHACTR1 | Intron | A | 0.92 | 0.08 | 0.05 | 0.1265 | +++-+ | A | 0.72 | 0.08 | 0.04 | 0.026915 | -+++++-+ | 30.7 | 0.18 |
| rs1332841 | 6 | 12875054 | PHACTR1 | Intron | T | 0.24 | -0.03 | 0.04 | 0.4266 | --++- | T | 0.36 | -0.08 | 0.04 | 0.028184 | +--+---+ | 0 | 0.44 |
| rs47711857 | 6 | 12880109 | PHACTR1 | Intron | NOT AVAILABLE | | | | | | T | 0.36 | -0.08 | 0.04 | 0.0302 | +--+---+ | 15 | 0.31 |
| rs944767 | 6 | 12788029 | PHACTR1 | Intron | C | 0.75 | 0.09 | 0.04 | 0.0084 | ++-++ | C | 0.86 | -0.10 | 0.05 | 0.032359 | ---+-+-+ | 38.6 | 0.12 |
| rs1237440 | 6 | 13232933 | PHACTR1 | Intron | NOT AVAILABLE | | | | | | T | 0.07 | -0.21 | 0.10 | 0.032359 | ?-?+--?? | 14.5 | 0.32 |
| rs595864 | 6 | 13251072 | PHACTR1 | Intron | T | 0.91 | 0.05 | 0.05 | 0.3464 | --++- | T | 0.75 | -0.09 | 0.04 | 0.032359 | ------+- | 0 | 0.84 |
| rs6913397 | 6 | 12809646 | PHACTR1 | Intron | NOT AVAILABLE | | | | | | A | 0.05 | -0.24 | 0.12 | 0.033113 | ?-?---?? | 22.5 | 0.28 |
| rs6920554 | 6 | 13183895 | PHACTR1 | Intron | NOT AVAILABLE | | | | | | C | 0.03 | 0.35 | 0.16 | 0.033884 | ?-?++??? | 34.7 | 0.22 |
| rs3817735 | 6 | 13278648 | PHACTR1 | Intron | A | 0.22 | 0.05 | 0.04 | 0.18 | +-+-+ | A | 0.48 | -0.08 | 0.04 | 0.033884 | ---+---+ | 0 | 0.95 |
| rs202055 | 6 | 13245617 | PHACTR1 | Intron | T | 0.06 | -0.04 | 0.06 | 0.5032 | +---- | T | 0.04 | -0.35 | 0.17 | 0.034674 | ???+--?? | 0 | 0.46 |
| rs9395425 | 6 | 13111743 | PHACTR1 | Intron | NOT AVAILABLE | | | | | | A | 0.94 | 0.20 | 0.10 | 0.035481 | ?+?+++?+ | 0 | 0.79 |
| rs1512414 | 6 | 13276415 | PHACTR1 | Intron | A | 0.22 | 0.05 | 0.04 | 0.1765 | +-+-+ | A | 0.56 | -0.07 | 0.03 | 0.035481 | ----+--+ | 0 | 0.75 |
| rs9381809 | 6 | 13182117 | PHACTR1 | Intron | C | 0.30 | -0.01 | 0.03 | 0.758 | -+-++ | C | 0.07 | -0.17 | 0.08 | 0.036308 | +-?+--+- | 15.8 | 0.31 |
| rs6936147 | 6 | 12739711 | PHACTR1 | Intron | NOT AVAILABLE | | | | | | T | 0.12 | 0.12 | 0.06 | 0.037154 | ?++++-+- | 39 | 0.13 |
| rs9473486 | 6 | 13109528 | PHACTR1 | Intron | A | 0.44 | -0.02 | 0.03 | 0.4574 | +---- | A | 0.75 | 0.08 | 0.04 | 0.037154 | -+++-+++ | 64.6 | 0.006 |
| rs6930015€ | 6 | 13179961 | PHACTR1 | Intron | A | 0.30 | -0.02 | 0.03 | 0.6141 | -+-++ | T | 0.18 | -0.10 | 0.05 | 0.037154 | ?----??? | 0 | 0.92 |
| rs202051 | 6 | 13274123 | PHACTR1 | Intron | T | 0.07 | 0.02 | 0.06 | 0.7143 | +--++ | T | 0.09 | 0.18 | 0.09 | 0.037154 | ?+?-++-+ | 49.5 | 0.08 |
| rs7748124 | 6 | 13054501 | PHACTR1 | Intron | T | 0.44 | -0.03 | 0.03 | 0.3232 | +---- | T | 0.81 | 0.09 | 0.04 | 0.039811 | -++-++-- | 48.6 | 0.06 |
| rs175729 | 6 | 13241144 | PHACTR1 | Intron | T | 0.94 | 0.04 | 0.06 | 0.5106 | -++++ | T | 0.71 | -0.08 | 0.04 | 0.039811 | -+-+---- | 11.1 | 0.34 |
| rs9349494 | 6 | 13173147 | PHACTR1 | Intron | A | 0.30 | -0.02 | 0.03 | 0.5876 | -+-++ | A | 0.07 | -0.18 | 0.09 | 0.040738 | +-?+---- | 11.6 | 0.34 |
| rs9473689 | 6 | 13207373 | PHACTR1 | Intron | NOT AVAILABLE | | | | | | A | 0.09 | 0.17 | 0.08 | 0.040738 | ?-?++++? | 19.7 | 0.29 |
| rs13215271 | 6 | 12817748 | PHACTR1 | Intron | A | 0.91 | 0.08 | 0.05 | 0.1305 | +-+++ | A | 0.90 | 0.11 | 0.05 | 0.041687 | +++++--- | 0 | 0.88 |
| rs9473524 | 6 | 13144838 | PHACTR1 | Intron | A | 0.42 | 0.005 | 0.03 | 0.8782 | +---+ | A | 0.63 | 0.07 | 0.04 | 0.043652 | -++++-++ | 37.5 | 0.13 |
| rs9369612 | 6 | 12853192 | PHACTR1 | Intron | T | 0.91 | 0.05 | 0.05 | 0.3771 | +++-- | T | 0.85 | -0.10 | 0.05 | 0.044668 | -+------ | 0 | 0.94 |
| rs6915585 | 6 | 12831273 | PHACTR1 | Intron | A | 0.09 | -0.05 | 0.05 | 0.2964 | ---++ | A | 0.16 | 0.10 | 0.05 | 0.045709 | ++++-++- | 0 | 0.59 |
| rs1953088 | 6 | 12925936 | PHACTR1 | Intron | A | 0.63 | 0.14 | 0.03 | 4.97E-06 | +++-+ | A | 0.22 | -0.08 | 0.04 | 0.046774 | +-++---- | 31.4 | 0.18 |
| rs1398299€ | 6 | 13260672 | PHACTR1 | Intron | A | 0.94 | 0.04 | 0.06 | 0.5064 | -++++ | A | 0.11 | 0.34 | 0.17 | 0.047863 | ?????+?? | 0 | 1 |

*First block of SNPs are from O’Donnell et al [[12](#_ENREF_12)] and have AA CAC effect in same direction; second block of SNPs are from O’Donnell et al and have AA CAC effect in opposite direction; Third block of SNPs are from MIGEN[[29](#_ENREF_29)] and CARDIoGRAM[[27](#_ENREF_27)]; Fourth block are the top SNPs (p<0.05) associated with AA CAC in *PHACTR1*.

†Chrom=chromosome; SE=Standard error; p=p-value; het p=heterozygosity p-value.

‡If no role indicated, then is outside known gene boundaries.

§Order of studies: FamHS, JHS, CARDIA, JHS-ARIC, MESA, MESA Family/Air, GeneSTAR, GENOA. GWAS results from each study were completed independently, thus data availability varied by study depending on study specific imputation quality and genotyping quality control for each SNP. Therefore not all studies had results for all SNPs, and when a study did not have a specific SNP a ‘?’ is given for direction.

$\parallel$Top SNPs from prior studies for AA CAD (rs3217989)[[28](#_ENREF_28)], AA CHD (rs6475606)[[19](#_ENREF_19)], EA CAD (rs4977574)[[26](#_ENREF_26)], and EA CAC (rs1333049)[[12](#_ENREF_12)].

¥From MIGEN [[29](#_ENREF_29)], second row is from EA CAC[[12](#_ENREF_12)]

€ SNPs where proxies were used in EA CAC look-up: AA SNP rs9367373=proxy EA SNP rs3864314; AA SNP rs436268=proxy EA SNP rs4711949; AA SNP 6930015=proxy EA SNP rs9463511; AA SNP rs1398299=proxy EA SNP rs15694118

**Supplemental Table S8. Attributes of top *PHACTR1* SNPs from O’Donnell^†^, CARDIoGRAM^¥^, MIGEN^§^, and AA CAC^‡^ obtained from HapMap.**

|  |  |  | **EA** | | **AA** | |
| --- | --- | --- | --- | --- | --- | --- |
| **SNP1** | **SNP2** | **Distance** | **R^2^** | **D’** | **R^2^** | **D’** |
| rs9349379^†,¥^ | rs2026458^†,¥^ | 78.1kb | 0.374 | 0.715 | 0.559 | 0.851 |
|  | rs12526453^¥,§^ | 23.5kb | 0.317 | 0.968 | 0.003 | 1 |
|  | rs7768030^‡^ | 80.9kb | 0.03 | 0.735 | 0.007 | 1 |
| rs2026458^†, ¥^ | rs12526453^¥,§^ | 101.6kb | 0.197 | 0.711 | 0.005 | 1 |
|  | rs7768030^‡^ | 2.9kb | 0.064 | 1 | 0.009 | 1 |
| rs12526453^¥,§^ | rs7768030^‡^ | 104.5kb | 0.048 | 0.542 | 0.007 | 0.524 |

^†^SNPs identified in O’Donnell et al.[[12](#_ENREF_12)] for CAC in EA.

^¥^SNPs identified in CARDIoGRAM [[27](#_ENREF_27)] for CAD/MI in EA.

^§^SNPs identified in MIGEN[[29](#_ENREF_29)] for MI in EA.

^‡^top SNP in *PHACTR1* in our AA CAC meta-analysis

**SUPPLEMENTAL FIGURE LEGENDS.**

**Supplemental Figure S1**. **Quantile-quantile plots of AA CAC GWAS results from each study.**

**Supplemental Figure S2. Linkage disequilibrium plots from HapMap.** A) For the CEPH population and B) For the YRI population, both for the region of *PHACTR1*  from 12800 kb-13100 kb. Blue arrow points to lead EA CAC SNP from O’Donnell et al, rs9349379; purple arrow points to AA CAC meta-analysis lead SNP in *PHACTR1*, rs7768030; green arrow is rs2026458 from O’Donnell et al; orange arrow is rs12526453 from MIGEN and CardioGRAM. As depicted, there is vastly different LD structure between these populations and these SNPs are in different LD blocks.

**Supplemental Figure S3. Regional plots of association results for the region from 12.7 Mb – 13.3 Mb in *PHACTR1.*** This uses A) EA CAC data from FamHS (in house data) and B) AA CAC meta-analysis results. There is little LD between the two top hits, rs9349379 in EA and rs7768030 in AA (purple diamonds in figure); however they may be tagging some common underlying functional variant that is not genotyped.

**SUPPLEMENTAL REFERENCES**

1. Higgins M, Province M, Heiss G, Eckfeldt J, Ellison RC, Folsom AR, Rao DC, Sprafka JM, Williams R: **NHLBI Family Heart Study: objectives and design**. *American journal of epidemiology* 1996, **143**(12):1219-1228.

2. Zhang Q, Lewis CE, Wagenknecht LE, Myers RH, Pankow JS, Hunt SC, North KE, Hixson JE, Jeffrey Carr J, Shimmin LC *et al*: **Genome-wide admixture mapping for coronary artery calcification in African Americans: the NHLBI Family Heart Study**. *Genetic epidemiology* 2008, **32**(3):264-272.

3. Taylor HA, Jr., Wilson JG, Jones DW, Sarpong DF, Srinivasan A, Garrison RJ, Nelson C, Wyatt SB: **Toward resolution of cardiovascular health disparities in African Americans: design and methods of the Jackson Heart Study**. *Ethnicity & disease* 2005, **15**(4 Suppl 6):S6-4-17.

4. Friedman GD, Cutter GR, Donahue RP, Hughes GH, Hulley SB, Jacobs DR, Jr., Liu K, Savage PJ: **CARDIA: study design, recruitment, and some characteristics of the examined subjects**. *Journal of clinical epidemiology* 1988, **41**(11):1105-1116.

5. Bild DE, Bluemke DA, Burke GL, Detrano R, Diez Roux AV, Folsom AR, Greenland P, Jacob DR, Jr., Kronmal R, Liu K *et al*: **Multi-ethnic study of atherosclerosis: objectives and design**. *Am J Epidemiol* 2002, **156**(9):871-881.

6. **Multi-center genetic study of hypertension: The Family Blood Pressure Program (FBPP)**. *Hypertension* 2002, **39**(1):3-9.

7. Irvin MR, Wineinger NE, Rice TK, Pajewski NM, Kabagambe EK, Gu CC, Pankow J, North KE, Wilk JB, Freedman BI *et al*: **Genome-wide detection of allele specific copy number variation associated with insulin resistance in African Americans from the HyperGEN study**. *PloS one* 2011, **6**(8):e24052.

8. Carr JJ, Nelson JC, Wong ND, McNitt-Gray M, Arad Y, Jacobs DR, Jr., Sidney S, Bild DE, Williams OD, Detrano RC: **Calcified coronary artery plaque measurement with cardiac CT in population-based studies: standardized protocol of Multi-Ethnic Study of Atherosclerosis (MESA) and Coronary Artery Risk Development in Young Adults (CARDIA) study**. *Radiology* 2005, **234**(1):35-43.

9. Budoff MJ, Katz R, Wong ND, Nasir K, Mao SS, Takasu J, Kronmal R, Detrano RC, Shavelle DM, Blumenthal RS *et al*: **Effect of scanner type on the reproducibility of extracoronary measures of calcification: the multi-ethnic study of atherosclerosis**. *Academic radiology* 2007, **14**(9):1043-1049.

10. Carr JJ, Danitschek JA, Goff DC, Crouse JR, 3rd, D'Agostino R, Chen MY, Burke GL: **Coronary artery calcium quantification with retrospectively gated helical CT: protocols and techniques**. *The international journal of cardiovascular imaging* 2001, **17**(3):213-220.

11. Agatston AS, Janowitz WR, Hildner FJ, Zusmer NR, Viamonte M, Jr., Detrano R: **Quantification of coronary artery calcium using ultrafast computed tomography**. *Journal of the American College of Cardiology* 1990, **15**(4):827-832.

12. O'Donnell CJ, Kavousi M, Smith AV, Kardia SL, Feitosa MF, Hwang SJ, Sun YV, Province MA, Aspelund T, Dehghan A *et al*: **Genome-wide association study for coronary artery calcification with follow-up in myocardial infarction**. *Circulation* 2011, **124**(25):2855-2864.

13. Djousse L, Arnett DK, Carr JJ, Eckfeldt JH, Hopkins PN, Province MA, Ellison RC: **Dietary linolenic acid is inversely associated with calcified atherosclerotic plaque in the coronary arteries: the National Heart, Lung, and Blood Institute Family Heart Study**. *Circulation* 2005, **111**(22):2921-2926.

14. Ellison RC, Zhang Y, Wagenknecht LE, Eckfeldt JH, Hopkins PN, Pankow JS, Djousse L, Carr JJ: **Relation of the metabolic syndrome to calcified atherosclerotic plaque in the coronary arteries and aorta**. *The American journal of cardiology* 2005, **95**(10):1180-1186.

15. Bild DE, Detrano R, Peterson D, Guerci A, Liu K, Shahar E, Ouyang P, Jackson S, Saad MF: **Ethnic differences in coronary calcification: the Multi-Ethnic Study of Atherosclerosis (MESA)**. *Circulation* 2005, **111**(10):1313-1320.

16. Budoff MJ, Takasu J, Katz R, Mao S, Shavelle DM, O'Brien KD, Blumenthal RS, Carr JJ, Kronmal R: **Reproducibility of CT measurements of aortic valve calcification, mitral annulus calcification, and aortic wall calcification in the multi-ethnic study of atherosclerosis**. *Academic radiology* 2006, **13**(2):166-172.

17. Heath SC: **Markov chain Monte Carlo segregation and linkage analysis for oligogenic models**. *American journal of human genetics* 1997, **61**(3):748-760.

18. Abecasis GR, Cherny SS, Cookson WO, Cardon LR: **GRR: graphical representation of relationship errors**. *Bioinformatics* 2001, **17**(8):742-743.

19. Lettre G, Palmer CD, Young T, Ejebe KG, Allayee H, Benjamin EJ, Bennett F, Bowden DW, Chakravarti A, Dreisbach A *et al*: **Genome-wide association study of coronary heart disease and its risk factors in 8,090 African Americans: the NHLBI CARe Project**. *PLoS genetics* 2011, **7**(2):e1001300.

20. Purcell S, Neale B, Todd-Brown K, Thomas L, Ferreira MA, Bender D, Maller J, Sklar P, de Bakker PI, Daly MJ *et al*: **PLINK: a tool set for whole-genome association and population-based linkage analyses**. *American journal of human genetics* 2007, **81**(3):559-575.

21. Li Y, Willer CJ, Ding J, Scheet P, Abecasis GR: **MaCH: using sequence and genotype data to estimate haplotypes and unobserved genotypes**. *Genetic epidemiology* 2010, **34**(8):816-834.

22. Huang L, Li Y, Singleton AB, Hardy JA, Abecasis G, Rosenberg NA, Scheet P: **Genotype-imputation accuracy across worldwide human populations**. *American journal of human genetics* 2009, **84**(2):235-250.

23. Price AL, Patterson NJ, Plenge RM, Weinblatt ME, Shadick NA, Reich D: **Principal components analysis corrects for stratification in genome-wide association studies**. *Nature genetics* 2006, **38**(8):904-909.

24. Patterson N, Price AL, Reich D: **Population structure and eigenanalysis**. *PLoS genetics* 2006, **2**(12):e190.

25. Psaty BM, O'Donnell CJ, Gudnason V, Lunetta KL, Folsom AR, Rotter JI, Uitterlinden AG, Harris TB, Witteman JC, Boerwinkle E: **Cohorts for Heart and Aging Research in Genomic Epidemiology (CHARGE) Consortium: Design of prospective meta-analyses of genome-wide association studies from 5 cohorts**. *Circulation Cardiovascular genetics* 2009, **2**(1):73-80.

26. **A genome-wide association study in Europeans and South Asians identifies five new loci for coronary artery disease**. *Nature genetics* 2011, **43**(4):339-344.

27. Schunkert H, Konig IR, Kathiresan S, Reilly MP, Assimes TL, Holm H, Preuss M, Stewart AF, Barbalic M, Gieger C *et al*: **Large-scale association analysis identifies 13 new susceptibility loci for coronary artery disease**. *Nature genetics* 2011, **43**(4):333-338.

28. Kral BG, Mathias RA, Suktitipat B, Ruczinski I, Vaidya D, Yanek LR, Quyyumi AA, Patel RS, Zafari AM, Vaccarino V *et al*: **A common variant in the CDKN2B gene on chromosome 9p21 protects against coronary artery disease in Americans of African ancestry**. *Journal of human genetics* 2011, **56**(3):224-229.

29. Kathiresan S, Voight BF, Purcell S, Musunuru K, Ardissino D, Mannucci PM, Anand S, Engert JC, Samani NJ, Schunkert H *et al*: **Genome-wide association of early-onset myocardial infarction with single nucleotide polymorphisms and copy number variants**. *Nature genetics* 2009, **41**(3):334-341.
