# Supplementary material for: Genetics of coronary artery calcification among African Americans, a meta-analysis
Source: BMC Med Genet. 2013 Jul 19;14:75. doi: 10.1186/1471-2350-14-75 (PMC3733595; doi:10.1186/1471-2350-14-75)
Supplement: Additional file 2: Figure S1 — Quantile-quantile plots of AA CAC GWAS results from each study. [file 1471-2350-14-75-S2.pptx]

## Slide 1
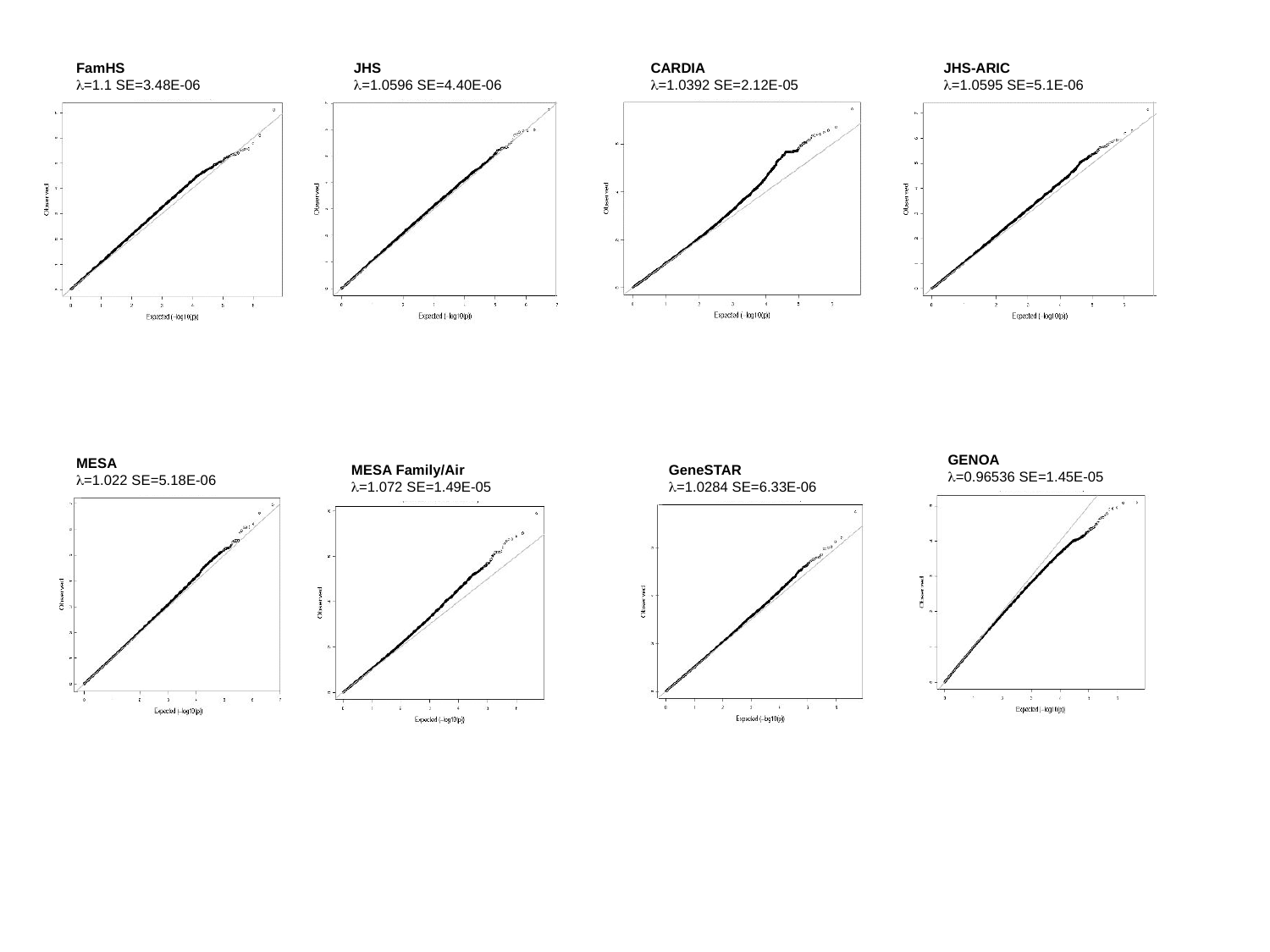

FamHS
l=1.1 SE=3.48E-06
JHS
l=1.0596 SE=4.40E-06
CARDIA
l=1.0392 SE=2.12E-05
JHS-ARIC
l=1.0595 SE=5.1E-06
GENOA
l=0.96536 SE=1.45E-05
MESA
l=1.022 SE=5.18E-06
MESA Family/Air
l=1.072 SE=1.49E-05
GeneSTAR
l=1.0284 SE=6.33E-06
