# Supplementary figures and images for: Genetics of coronary artery calcification among African Americans, a meta-analysis
Source: BMC Med Genet. 2013 Jul 19;14:75. doi: 10.1186/1471-2350-14-75 (PMC3733595; doi:10.1186/1471-2350-14-75)

## Slide 1
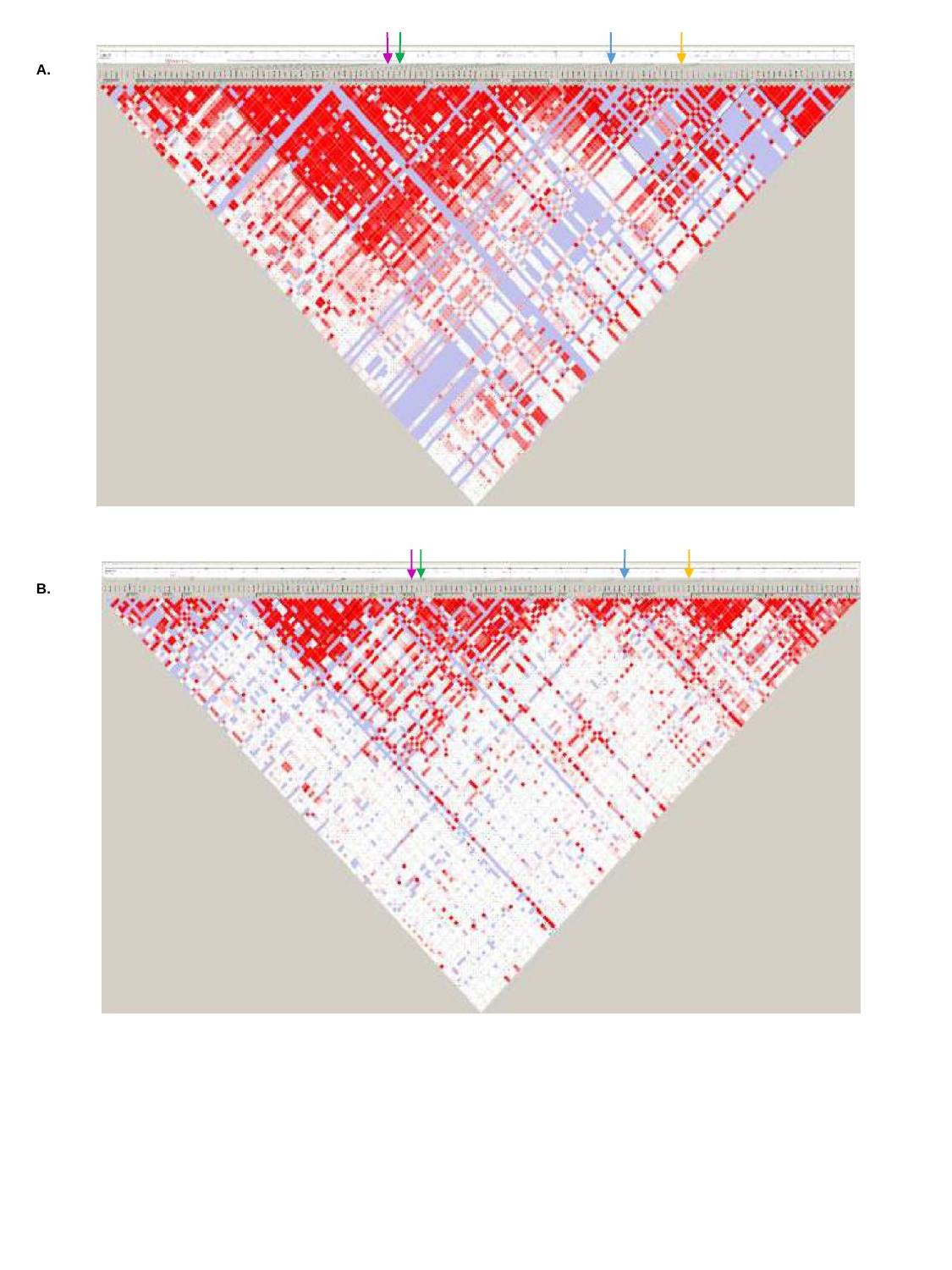

A.
B.

Supplement: Additional file 3: Figure S2 — Linkage disequilibrium plots from HapMap. A) For the CEPH population and B) For the YRI population, both for the region of PHACTR1 from 12800 kb-13100 kb. Blue arrow points to lead EA CAC SNP from O’Donnell et al, rs9349379; purple arrow points to AA CAC meta-analysis lead SNP in PHACTR1, rs7768030; green arrow is rs2026458 from O’Donnell et al; orange arrow is rs12526453 from MIGEN and CardioGRAM. As depicted, there is vastly different LD structure between these populations and these SNPs are in different LD blocks. [file 1471-2350-14-75-S3.pptx]

## Slide 1
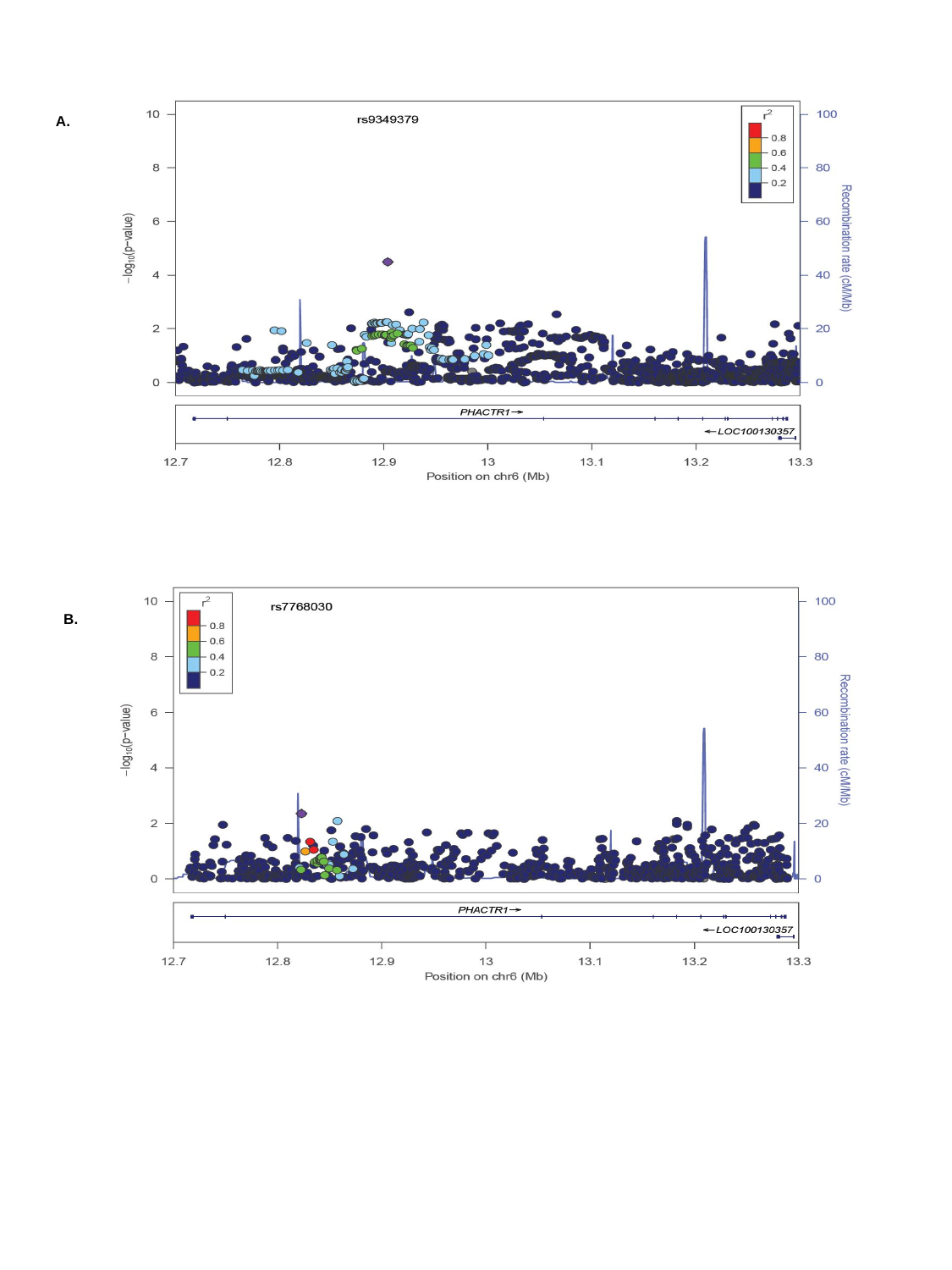

A.
B.

Supplement: Additional file 4: Figure S3 — Regional plots of association results for the region from 12.7 Mb – 13.3 Mb in PHACTR1. This uses A) EA CAC data from FamHS (in house data) and B) AA CAC meta-analysis results. There is little LD between the two top hits, rs9349379 in EA and rs7768030 in AA (purple diamonds in figure); however they may be tagging some common underlying functional variant that is not genotyped. [file 1471-2350-14-75-S4.pptx]
